# Long Non-Coding RNA CD27-AS1-208 Facilitates Melanoma Progression by Activating STAT3 Pathway

**DOI:** 10.3389/fonc.2021.818178

**Published:** 2022-01-13

**Authors:** Jingjing Ma, Qiong Shi, Sen Guo, Peng Xu, Xiuli Yi, Yuqi Yang, Weigang Zhang, Yu Liu, Lin Liu, Qiao Yue, Tao Zhao, Tianwen Gao, Weinan Guo, Chunying Li

**Affiliations:** Department of Dermatology, Xijing Hospital, Fourth Military Medical University, Xi’an, China

**Keywords:** melanoma, LncRNAs, CD27-AS1-208, STAT3, therapeutic targets

## Abstract

Melanoma is the most lethal skin cancer that originates from epidermal melanocytes. Recently, long non-coding RNAs (lncRNAs) are emerging as critical regulators of cancer pathogenesis and potential therapeutic targets. However, the expression profile of lncRNAs and their role in melanoma progression have not been thoroughly investigated. Herein, we firstly obtained the expression profile of lncRNAs in primary melanomas using microarray analysis and unveiled the differentially-expressed lncRNAs compared with nevus. Subsequently, a series of bioinformatics analysis showed the great involvement of dysregulated lncRNAs in melanoma biology and immune response. Further, we identified lncRNA CD27-AS1-208 as a novel nuclear-localized factor with prominent facilitative role in melanoma cell proliferation, invasion and migration. Mechanistically, CD27-AS1-208 could directly interact with STAT3 and contribute to melanoma progression in a STAT3-dependent manner. Ultimately, the role of CD27-AS1-208 in melanoma progression *in vivo* was also investigated. Collectively, the present study offers us a new horizon to better understand the role of lncRNAs in melanoma pathogenesis and demonstrates that CD27-AS1-208 up-regulation contributes to melanoma progression by activating STAT3 pathway. Targeting CD27-AS1-208 in melanoma cells can be exploited as a potential therapeutic approach that needs forward validation in clinical trials in the future.

## Introduction

Cutaneous melanoma, which results from the malignant transformation of epidermal melanocytes, is the most aggressive type of skin cancer and accounts for the vast majority of the death of patients with skin cancers (1). Although MAPK-targeting therapies and immune checkpoint blockades are emerging as effective therapeutic approaches, low response rate and the occurrence of treatment resistance prominently hinder the efficacy (2, 3). Further understanding of the mechanisms underlying melanoma progression will facilitate the development of alternative therapeutic strategies and help the management of melanoma patients.

Long non-coding RNA (lncRNA) refers to a transcript >200 nt in length that is not translated to proteins (4). According to their genome location and context, lncRNAs are classified into four groups: intergenic, intronic, sense and antisense lncRNAs (4). LncRNAs can serve as scaffolds or guides to regulate protein-protein or protein-DNA interactions, as decoys to bind proteins or microRNAs (miRNAs), and as transcriptional regulators to modulate the transcription of neighboring genes by cis-acting mechanisms or in- trans without affecting neighboring genes (4). Recent reports have identified several lncRNAs that can function as critical oncogenes or tumor suppressors in melanoma and these lncRNAs may represent a new class of clinically relevant therapeutic targets for melanoma treatment (5, 6). Specifically, the lncRNA SAMMSON is required for the growth of melanoma cells and has the potential to be used both as a new biomarker and as a highly selective therapeutic target for treatment (7). In addition, lncRNA GAS5 has been proved to be down-regulated in melanoma tissues and inhibits growth, migration and invasion of melanoma cells (8). Though these reports have brought some insights into the role of lncRNAs in melanoma pathogenesis, the expression profile of lncRNAs in melanoma has not been thoroughly investigated and their pathogenic effects remain far from understood.

Signal transducer and activator of transcription (STAT3) is a nuclear transcription factor of STAT family. After being phosphorylated at Tyr705, it trans-locates into nucleus to induce the expression of target genes, including genes related to proliferation (*Mcl-1*, *cyclind1*, *Myc*), metastasis (*Vegf, hif1α*) and apoptosis (*Bcl2*, *survivin*) (9). Accumulative evidence has revealed that STAT3 is hyper-activated in melanoma and contributes to tumor initiation and progression through the regulation of cell proliferation, metastasis and immune evasion (10–18). The phosphorylation of STAT3 at Tyr705 is positively associated with melanoma development and is a negative prognostic factor for overall survival of melanoma patients (12, 18). In addition, the activation of STAT3 signaling facilitates the growth and survival of melanoma cells by promoting the expression of the anti-apoptotic molecules Bcl-xL and Mcl-1 (13). Therefore, targeting STAT3 is a promising strategy for melanoma treatment (19). Of note, as an intracellular transcriptional factor that lacks enzymatic activity, STAT3 has often been considered as an undruggable target, and the development of potential inhibitors is is rather difficult (20). Hence, to further explore the upstream mechanism that regulates STAT3 activation will facilitate the development of alternative approach for melanoma therapy. Previous reports have shed some lights into the regulation of STAT3 by lncRNAs in other cancers or other types of cell. To be specific, lncRNA LNRRIL6 physically binds to the IL-6 promoter, thereby increasing IL-6 transcription and autocrine, and the activating IL-6/STAT3 pathway in colorectal cancer cells (21). In addition, lncRNA ITIH4-AS1 forms a complex with FUS and STAT3, and helped STAT3 nuclear translocation to activate JAK/STAT3 signaling, exerting pro-tumor functions in colorectal cancer (22). What’s more, TSLNC8 competitively interacts with transketolase (TKT) and STAT3, and modulates the STAT3-Tyr705 and STAT3-Ser727 phosphorylation levels and STAT3 transcriptional activity, thus resulting in the inactivation of the IL-6/STAT3 signaling pathway and suppressive growth of hepatocellular carcinoma (23). These reports indicate that these lncRNAs are critical upstream-regulators of STAT3 signaling. Targeting STAT3 *via* intervening lncRNAs may be of great therapeutic value for melanoma therapy.

Herein, we firstly obtained the expression profile of lncRNAs in primary melanoma using microarray analysis and unveiled the differentially-expressed lncRNAs in melanoma compared with nevus. Subsequently, a series of bioinformatics analysis showed the great implication of dys-regulated lncRNAs in melanoma biology and immune response. Further, we identified lncRNA CD27-AS1-208 as a novel nuclear-localized factor with prominent facilitative effect on melanoma cell proliferation, migration and invasion. Mechanistically, CD27-AS1-208 could directly interact with STAT3 and contribute to melanoma progression in a STAT3-dependent manner. Ultimately, the role of CD27-AS1-208 in melanoma progression *in vivo* was also investigated.

## Material And Methods

### Clinical Specimens

The clinical specimens of nevus, primary melanomas and metastasis melanomas were collected from Xijing Hospital, Fourth Military Medical University (Xi’an, China). The main clinical and basic information of the patients and clinical specimens are summarized in **Table 1** and **Table S1**. All subjects were Chinese Han people with no previous systemic treatment. All tissues samples were frozen in liquid nitrogen immediately after resection and stored at liquid nitrogen. The study was approved by the ethics review board of Fourth Military Medical University (Xi’an, China). Written informed consent was obtained from all patients, according to the principles of the Declaration of Helsinki. Both tumor and nevus samples were confirmed by pathological examination.

**Table 1:** Sample information of primary MM and Nevus used in lncRNAs microarray analysis.

Primary MM						Nevus		
Number	Gender	Age(y)	Clinical stage	Breslow thickness (mm)	Ki67 (%)	Numer	Gender	Age(y)
1	Female	50	IIB	4.0	60	1	Female	51
2	Female	30	IIB	3.8	20	2	Female	31
3	Male	41	IIC	4.8	20	3	Male	45

MM, melanoma.

### LncRNAs and mRNAs Microarray

Total RNA was extracted from 3 pairs of primary melanoma and nevus tissues using Trizol reagent (Life Technologies, Carlsbad, CA) according to manufacturer’s protocol. Agilent human lncRNA+mRNA Array v4.0 (Agilent Technologies, Santa Clara, CA) was used for microarray hybridization performed by Capital Biotech (Beijing, China). The array data were analyzed for summarization, normalization and quality control by using the GeneSpring software V13.0 (Agilent Technologies, Santa Clara, CA). Genes having a fold change ≥ 2 or ≤ −2 and a *P* < 0.05 were considered as differentially expressed.

Hierarchical clustering analysis was conducted to elucidate the lncRNAs and mRNAs expression pattern. Differentially-expressed lncRNAs and mRNAs with statistical significance were displayed through volcano plot. The microarray data were deposited in Gene Expression Omnibus (GEO) database (accession No.: GSE183878) in the NCBI database.

### LncRNAs-mRNAs Co-Expression Network Construction

The lncRNAs-mRNAs co-expression network was constructed based on the correlation analysis between the differentially-expressed lncRNAs and mRNAs. For each pair of genes, the Pearson correlation was calculated and the significant correlation pairs with *P*<0.05 and Pearson correlation coefficients not less than 0.99 were selected to construct the network though the open source bioinformatics software Cytoscape. In a network analysis, a degree centrality is defined as the link numbers one node has to the other. A degree is the simplest and most important measures of a gene centrality within a network that determining the relative importance (24).

### GO Enrichment and KEGG Pathway Analysis

Gene Ontology (GO) analysis and Kyoto Encyclopedia of Genes (KEGG) pathway analysis were performed as described previously (25). Briefly, GO analysis was performed based on Gene Ontology (www.geneontology.org), which provides three structured networks of defined terms that describe gene product functions: biological processes, molecular functions, and cellular components. KEGG (KEGG, http://www.genome.jp/kegg/) database was used for pathway analysis of the differentially-expressed genes.

### Quantitative Real-Time PCR

Total RNA was isolated using Trizol reagent (Life Technologies, Carlsbad, CA) according to the manufacturer’s instruction. Quantitative Real-Time PCR (qRT-PCR) was carried out by using One Step SYBR^®^ PrimeScript™ RT-PCR Kit (Takara Bio, Kusatsu, Japan) on a Chromo4 continuous fluorescence detector with a PTC-200 DNA Engine Cycler (Bio-Rad, Hercules, CA) in accordance with the manufacturer’s protocols. Primers used for each gene were listed in **Table S2**. Relative expression of lncRNAs and mRNAs was calculated *via* the 2^-ΔΔCT^ method.

### Cell Lines and Culture Conditions

Normal human melanocytes NHEM and Hermes were provided by David Schrama (University Hospital Würzburg, Würzburg, Germany). The immortalized normal human epidermal melanocyte cell line PIG1 was a gift from Dr. Caroline Le Poole, Loyola University Chicago, Maywood, IL (26).WM793B, WM35, A375, WM-266, A2058, 451Lu, Hs294T, 1205Lu, SK-MEL-1 and SK-MEL-5 were purchased from American Type Culture Collection (ATCC) in 2014. All these cell lines were authenticated by short-tandem repeat (STR) fingerprinting by Beijing Microread Genetics Company Limited (Beijing, China) in 2016 and tested for mycoplasma contamination (27). NHEM and Hermes were grown in HAM’s F10 media supplemented with ITS premix (Becton Dickinson, Franklin Lakes, NJ), 12-Otetradecanoylphorbol-13-acetate and 3- isobutyl- 1- methylxanthine (Sigma-Aldrich, St Louis, MO), cholera toxin (List Biological Laboratories, Campbell, CA), 20% fetal bovine serum (FBS, Gibco, CA), and glutamine (Gibco, CA). PIG1 was cultured in Medium 254 (Invitrogen, Carlsbad, CA) supplemented with human melanocyte growth supplement, and 5% FBS (Gibco, CA). WM793B, WM35, 451Lu and 1205Lu were cultured in RPMI-1640 (Hyclone, Logan, UT). A375, WM- 266, Hs294T, SK-MEL-1 and SK-MEL-5 were maintained in DMEM (Hyclone, Logan, UT). A2058 was cultured in DMEM-F12 (Hyclone, Logan, UT). All the basic mediums for melanoma cell lines were supplemented with 2 mM glutamine and 10% FBS. All the cells were grown in a humidified culture incubator at 37°C and 5% CO_2_ atmosphere.

### Transfection With siRNAs

A2058 and A375 cell lines were transiently transfected with small interfering RNA (siRNA) targeting lncRNA CD27-AS1-208 or negative control siRNA (GenePharma, Shanghai, China) using Lipofectamine 3000 regent (Invitrogen, Carlsbad, CA) according to the manufacturer’s protocol. The two siRNAs sequences were as follows: siCD27-AS1-208-1 (5’ GACAAGGAGAGGGACAAAUTT 3’), siCD27-AS1-208-2 (5’ CCUCGUGUAUGCAUUCUCUTT 3’). The negative control siRNA (siNC) sequence was (5’UUCUCCGAACGUGUCACGUTT 3’). Knockdown efficiency was measured by qRT-PCR at 48h after transfection.

### Cell Proliferation Analysis

After transfection for different time points (24h, 48h, 72h, 96h), cell proliferation was assessed by a CCK-8 regent (7sabiotech, Shanghai, China) according to the manufacturer’s protocol. The absorbance at 450 nm was measured with a micro-plate reader (Bio-Rad, CA).

### Colony Formation Assay

Cells were harvested at 24h after transfection and seeded in 6-well plates with 3000 cells for each well. The plates were incubated at 37°C with 5% CO_2_ for 7-10 days. Following incubation, cell colonies were stained with crystal violet (Sigma-Aldrich, St Louis, MO) and counted.

### Cell Migration Assay

Cell migration detection using a wound healing assay was performed as previously described (27). In brief, at 24h after transfection, melanoma cell lines A2058 and A375 were scraped with a sterile 200μl pipette tip to generate a clear line in the wells, and wound closure was monitored at 24h after scratch with a phase contrast microscope at 100 × magnifications (Olympus, Tokyo, Japan).

### Invasion Assays

After 24h of transfection, cells in serum-free medium were seeded in the top chambers of 24-well transwell culture inserts (pore size, 8μm; diameter, 6.5mm, Corning, NY) coated with matrigel (BD Biosciences, NJ). After 24h, the non-invasive cells on the upper side of the chamber were removed with cotton buds, while the invasive cells on the lower side were stained with crystal violet (Sigma-Aldrich, St Louis, MO). Then the stained cells were photographed and counted under a phase contrast microscope at × 200 magnifications (Olympus, Tokyo, Japan).

### Cytoplasmic/Nuclear RNA Isolation

Melanoma cells were subjected to cytoplasmic and nuclear RNA isolation using a PARIS™ kit (ThermoFisher, Waltham, MA) following the recommended protocol.

### Semi-Quantitative Reverse Transcription With PCR

Total RNA was isolated using Trizol regent (Life Technologies, Carlsbad, CA) according to the manufacturer’s instruction. RNA (1 μg) from each sample was reversely transcribed into single- stranded cDNA by using PrimeScript RT reagent Kit (Takara, Ohtsu, Japan). Semi-quantitative analyses by reverse transcription with PCR (RT-PCR) were performed using Premix Taq™ (Takara, Ohtsu, Japan). The primers used were listed in **Table S2**. The products were electrophoresed in 1.2% agarose gel.

### Fluorescence *In Situ* Hybridization (FISH)

Paraffin-embedded tissues section (5 μm thick) derived from primary melanoma and nevus tissues were used along with a FAM-labeled CD27-AS1-208 oligonucleotide probe (Servicebio, Wuhan, China) in order to determine the lncRNA location. The sections were deparaffinized followed by treatment with 20 μg/ml proteinase-K at 37°C for 20 minutes, prehybridization in hybridization buffer at 37°C for 1h, and hybridization with CD27-AS1-208 probe (5’-FAM-CCCTATGGGGTCCCCTGCTGCT ACTCATTCTG- FAM-3’) at 37°C overnight. After washing with 2× SSC (Gibco, CA) for 10 min, 1× SSC for 10 min and 0.5× SSC at 25°C for 10 min each wash, DAPI (Dako, Denmark) was used to stain cell nucleus. Tissue sections were analyzed by confocal laser scanning microscopy (FV-1000, Olympus, Tokyo, Japan).

### Immunofluorescence Staining Analysis

For deparaffinized tissue sections (5 μm thick), antigen retrieval was performed in Tris-EDTA Buffer (10 mM Tris Base, 1 mM EDTA Solution, 0.05% Tween 20, pH 9.0). Subsequently, the tissue sections were blocked with goat serum for 1h and incubated with primary antibodies (mouse monoclonal anti-MelanA, Abcam; rabbit monoclonal anti-Ki67, Abcam; 1:200) overnight at 4°C. After washed with PBS, they were incubated with secondary antibodies (FITC or Cy3-tagged goat anti-rabbit, 1:200) for 1h, and then washed with PBS and further incubated with DAPI (1:1000, Dako, Glostrup, Denmark) for 15 min. Fluorescent images were obtained by an FV-1000 confocal microscope (Olympus, Tokyo, Japan).

### 5’- Rapid Amplification of cDNA Ends (RACE) and 3’-RACE

The 5’-RACE and 3’-RACE assays were performed to determine the transcriptional initiation and termination sites of lncRNA CD27-AS1-208 using GeneRacer™ Kit (Invitrogen, Carlsbad, CA) following the manufacturer’s instructions (28). The primers used for PCR of the RACE analysis were as follows: 5’-ATCTTTGGGCTGTAATAGGAGGGGACACAC-3’ (5’-RACE) and 5’-GGGACCCCATAGGGCACATCTGAAGG-3’ (3’-RACE).

### RNA Pull-Down

RNA pull-down assays were performed as previously described (29). In brief, LncRNA CD27-AS1-208 and negative control lncRNA CD27-AS1-208 antisense were transcribed *in vitro* using MAXIscript™ Kit (ThermoFisher, Waltham, MA) according to manufacturer’s instructions. Then, approximately 50 pmol biotinylated RNA from the previous step was added to 200 μg whole-cell lysate from A2058 cells to examine the protein binding to CD27-AS1-208 using Pierce™ Magnetic RNA-Protein Pull-Down Kit (ThermoFisher, Waltham, MA). The RNA-binding protein complexes were washed, eluted and analyzed by Mass spectrometry (MS) (5600-plus, AB SCIEX, MA).

### Western Blot Analysis

Equal amounts of protein samples were separated by denaturing SDS-polyacrylamide gel electrophoresis and transferred electrophoretically to nitrocellulose membranes (Millipore, Billerica, MA). Additional experiments were carried out as previously described (30).

The primary antibodies against phospho-STAT3 (Y705), STAT3, phospho-p44/42 MAPK (ERK1/2) and p44/42 MAPK (ERK1/2) were obtained from Cell Signaling Technology, Inc. (Danvers, MA). Primary antibody against prohibitin was purchased from Proteintech Group (Rosemont, IL). Primary antibody against β-Actin (CW0096M) was obtained from CWBIO (Beijing, China).

### Rac1 Activation Assay

Rac1 activation of cell samples was analyzed by the G-LISA^®^ Rac1 Activation Assay Biochem kit (Cytoskeleton, Inc, Denver) according to the manufacturer’s protocol.

### PP2A Activation Assay

The activity of PP2A in melanoma cells was measured by PP2A Immunoprecipitation Phosphatase Assay Kit (Millipore, Massachusetts) following the manufacturer’s protocol.

### Xenograft Mice Model

For subcutaneous injection, 2×10^6^ A2058 cells (in PBS) with the stably-silenced CD27-AS1-208 (shCD27-AS1-208) or negative control (shNC) were injected into the lower back region of 6-week-old female BALB/C-Nu nude mice, with eight mice randomly distributed into per group. Tumor volumes were measured at indicated time points. After 50 days, the viable mice were sacrificed and xenograft tumors were isolated and weighed. Tumors were fixed in 4% paraformaldehyde overnight. Paraffin-embedded sections were processed to immunofluorescence staining analysis. All animal experiments complied with ethical regulations and were approved by the Subcommittee on Research Animal Care of the Fourth Military Medical University.

### Statistical Analysis

Correlation analysis was performed using Spearman’s rank correlation coefficient analysis with GraphPad Prism 5 (San Diego, CA). Each experiment was performed at least for three times and statistical analyses were performed by Prism 5 (GraphPad software, San Diego, CA). One-way analysis of variance and the Bonferroni method were used to detect differences among multiple groups. The unpaired two-tailed Student’s t test was used for comparisons between two groups. Data represent mean ± S.D. for at least 3 independent experiments. ^*^ *P* < 0.05, ^**^ *P* < 0.01 and ^***^ *P* < 0.001 were considered to be statistically significant.

## Results

### The Expression Profiles of lncRNAs in Primary Melanoma

**Figure 1:**
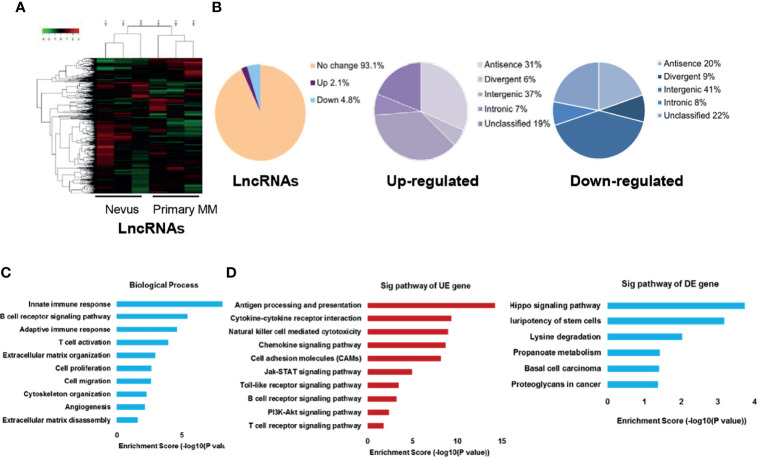
Characterization of lncRNAs expression profile in primary melanoma compared with nevus. **(A)** Hierarchical clustering results of differentially-expressed lncRNAs in primary MM and Nevus. “Green” indicates relative low expression while “red” indicates relative high expression. **(B)** Percentage of differentially-expressed lncRNAs in the subgroups, sorted by their genome location and context. **(C)** GO analysis of differentially-expressed coding genes co-expressed with differentially-expressed lncRNAs was performed in biological processes. **(D)** KEGG pathway analysis of differentially-expressed coding genes co-expressed with differentially-expressed lncRNAs was performed. The vertical and horizontal axis represents the biological processes/pathways and −log10 (*P* value) of the corresponding biological process/pathways, respectively. GO, gene ontology; KEGG, Kyoto Encyclopedia of Genes and Genomes; Sig, significant; UE, up-regulated expression; DE, down-regulated expression.

We employed microarray analysis in three paired primary melanomas and nevus tissues to reveal the specific expression profile of lncRNAs in melanoma (basal information and relevant clinical characteristics shown in **Table 1**). The hierarchical clustering analysis and the volcano plots showed the distinct signatures of lncRNAs between primary melanomas and nevus tissues (**Figures 1A** and **S1A**). In total, 1646 differentially-expressed lncRNAs were discovered, with 495 and 1151 lncRNAs displaying significant up- or down-regulation, respectively, in primary melanoma compared with nevus (*P* < 0.05; fold change ≥ 2). According to their genome location and context, the differentially-expressed lncRNAs were classified into four subgroups: divergent, intronic, sense and antisense with different percentages (**Figure 1B**). Meanwhile, according to chromosome location, the distribution of the differentially-expressed lncRNAs on each chromosome was also revealed (**Figure S1C**), suggesting the universality and complexity of dys-regulated lncRNAs related to genomic location and alteration.

LncRNAs generally exert their function *via* affecting the expression or activity of downstream target genes, and genes with the same biological function or implicated in the same biological pathway way usually have similar expression patterns (31, 32). Thus, the potential functions of lncRNAs in tumor biology might be predicted from the functions or pathways regulated by their co-expressed mRNAs. Thereafter, we obtained the mRNA expression profile from the same panel of primary melanomas and nevus tissues, and the hierarchical clustering analysis and volcano plots showed the distinct signatures of mRNAs between these two groups (**Figures S1A, B**). Then, we constructed lncRNA-mRNA co-expression network and conducted gene ontology (GO) and kyoto encyclopedia of genes and genomes (KEGG) pathway analysis on their co-expressed mRNAs. GO analysis revealed that biological process related to immune response, cell proliferation and cell migration were enriched (**Figure 1C**). Besides, KEGG pathway analysis showed that the enriched pathways of up-regulated lncRNAs were related to immunity, cell adhesion molecules, JAK-STAT, Toll-like receptor and PI3K-Akt signaling while the enriched pathways of down-regulated lncRNAs were associated with Hippo signaling, pluripotency of stem cells, lysine degradation and propanoate metabolism in cancer (**Figure 1D**). In addition, we also analyzed the co-expression status of dysregulated lncRNAs and critical immune-related mRNAs in our microarray data and found that the expressions of several lncRNAs were significantly correlated with the expressions of key immune regulators like LAG3, PRF1, GZMB and PD-1 (**Table S3**). We also conducted GO and KEGG pathway analysis of differentially-expressed lncRNAs and the results were similar to those of differentially-expressed mRNAs (**Figures S2A, B**).

### LncRNA CD27-AS1-208 Is Significantly Up-Regulated in Melanoma

**Figure 2:**
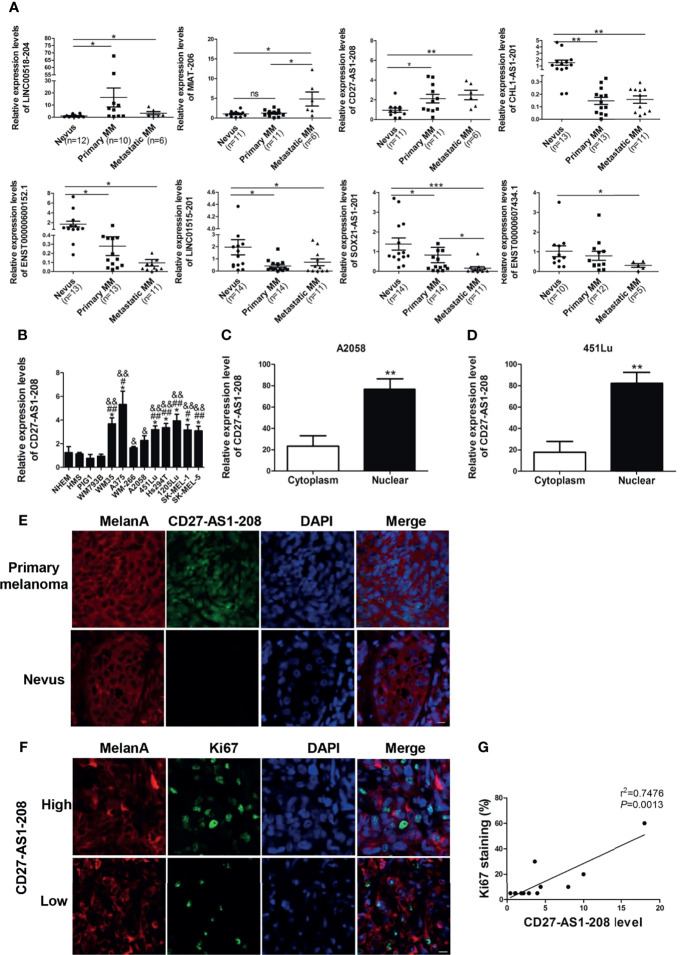
The expression of lncRNA CD27-AS1-208, which locates in the nucleus, is increased during melanoma progression and significantly correlation with Ki-67 level. **(A)** Validation of differentially-expressed lncRNAs with a new cohort of samples. **(B)** The expression level of CD27-AS1-208 in melanocytes and melanoma cell lines. **(C, D)** The expression level of CD27-AS1-208 in the cytoplasm and nucleus of melanoma cells. **(E)** The location of CD27-AS1-208 in primary melanoma and nevus. **(F)** Immunofluorescence staining of Ki-67 in indicated melanoma tissues. **(G)** Correlation between CD27-AS1-208 level and Ki67 staining in melanoma tissue. ^*^ *P* < 0.05, ^**^ *P* < 0.01, ^***^ *P* < 0.001, NHEM or cytoplasm as control; ^#^HMS as control, ^#^ *P* < 0.05, ^##^ *P* < 0.01; ^&^PIG1 as control, ^&^ *P* < 0.05, ^&&^ *P* < 0.01. Scale bar = 10μm. ns., not significant.

To verify the differentially-expressed lncRNAs obtained from microarray analysis, the most significantly up- or down-regulated lncRNAs (**Table 2**) were selected for validation in another cohort of samples (**Table S1**). As was shown, the expressions of lncRNAs LINC00518-204 and CD27-AS1-208 were significantly increased in primary and metastasis melanoma as compared with nevus, which was consistent with the microarray data. Besides, the expression of lncRNA MIAT-206 was elevated more significantly in metastasis melanoma than that in primary melanoma and nevus, while its expression in primary melanoma and nevus was comparable. What’s more, the expressions of lncRNAs CHL1-AS1-201, ENST00000600152.1, LINC01515-201 and SOX21-AS1-201 were remarkably reduced in primary melanoma as compared with nevus, which was also in consistent with the results of microarray analysis. Moreover, SOX21-AS1-201 and ENST00000607434.1 displayed the lowest expression level in metastasis melanoma (**Figure 2A**).

**Table 2:** Eight up- and down-regulated lncRNAs selected for validation.

Name	Transcript ID	Chr.	*P* value	Fold change	Up or down
LINC00518-204	ENST00000491317.1	6	0.016	37.963	up
MIAT-206	ENST00000425476.2	22	0.002	28.483	up
CD27-AS1-208	ENST00000538616.1	12	0.030	15.366	up
CHL1-AS1-201	ENST00000417612.1	3	0.031	32.047	down
Novel transcript	ENST00000600152.1	19	0.002	28.871	down
LINC01515-201	ENST00000433152.2	10	0.001	25.644	down
SOX21-AS1-201	ENST00000438290.2	13	0.018	25.035	down
Novel transcript	ENST00000607434.1	6	0.029	2.683	down

Chr., chromosome.

Among the above-mentioned three up-regulated lncRNAs, we selected CD27-AS1-208 for further investigation due to its increased expression during melanoma progression (**Figure 2A**). Meanwhile, we found that the expression levels of CD27-AS1-208 was significantly higher in melanoma cell lines than that in melanocytes (**Figure 2B**), which was in line with the results in tissues samples (**Figure 2A**). CD27-AS1-208 is one of the transcripts of CD27-AS1. Additionally, we explored the expression level of CD27-AS1 in other types of cancers by analyzing TCGA (The Cancer Genome Atlas) database and found that CD27-AS1 was also up-regulated in other types of tumors (**Figure S3**), indicating that the up-regulation of CD27-AS1 cluster is not specifically for melanoma.

CD27-AS1-208, located in chromosome 12, is an anti-sence RNA of CD27. As was shown, it was mainly localized in the nucleus, whereas was of only weak expression in the cytoplasm (**Figures 2C, D** and **S4**). In consistent, fluorescence *in situ* hybridization (FISH) analysis revealed that CD27-AS1-208 was mainly localized in the nucleus of melanoma cells (which specifically express MelanA) in primary melanoma tissues whereas almost absent in melanocytes of nevus tissues (**Figure 2E**).

In order to figure out the clinical implication of CD27-AS1-208 in melanoma progression, we went on to analyze the correlation between CD27-AS1-208 level and Ki-67 expression that has been well documented as a potent indicator of melanoma development (33). The representative images of high and low CD27-AS1-208 expression in primary melanoma were presented in **Figure S5**. It showed that CD27-AS1-208 level was in positive correlation with Ki-67 (**Figures 2F, G**), suggesting the potential of tumorous CD27-AS1-208 level as a valuable indicator of melanoma progression. Meanwhile, we also turned to TCGA Skin Cutaneous Melanoma (SKCM) database to analyze the correlation between CD27-AS1 level and the expressions of MITF, TYR and PMEL in melanoma. The results showed that CD27-AS1 level was not significantly correlated with either the expressions of MITF, TYR or PMEL, suggesting that the expression status of CD27-AS1 might not be associated with melanocytic lineage (**Figure S6**).

### CD27-AS1-208 Promotes Melanoma Cell Proliferation, Migration and Invasion

**Figure 3:**
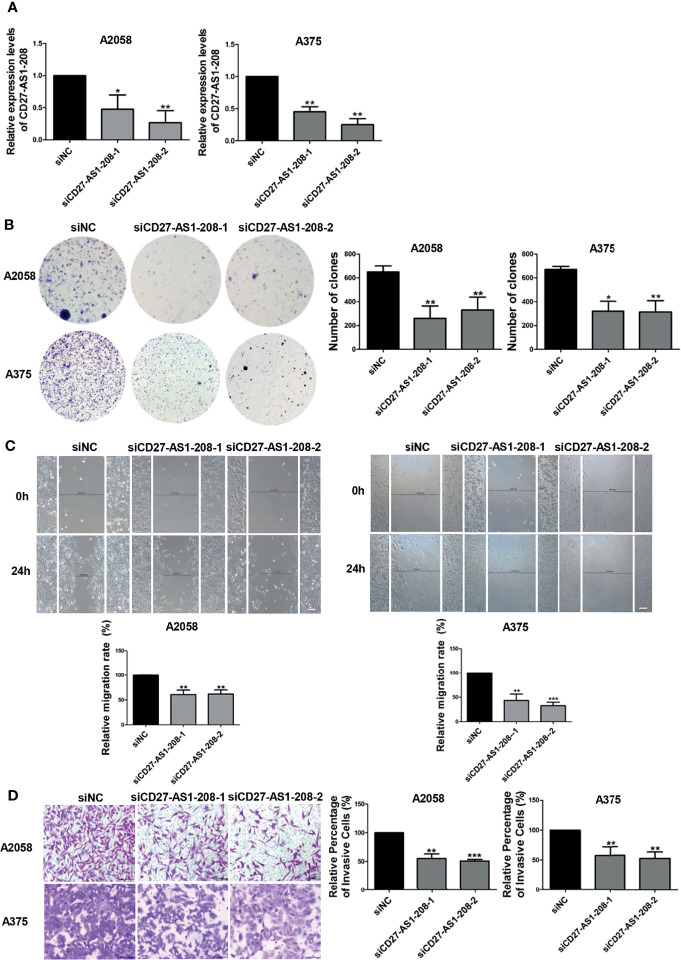
Knockdown of lncRNA CD27-AS1-208 inhibits melanoma cell proliferation, migration and invasion. **(A)** The knockdown efficiency of CD27-AS1-208 after 48h of transfection. **(B)** Clone formation assays of A2058 and A375 cells transfected with the indicated siRNA. **(C)** The migratory ability of A2058 and A375 cells transfected with the indicated siRNA was assessed by the wound healing assay. **(D)** A2058 and A375 cells transfected with siCD27-AS1-208 or NC siRNA were subjected to the matrigel invasion assay. Scale bar = 100μm. Data represent the mean ± SD of triplicates. ^*^ *P* < 0.05, ^**^ *P* < 0.01, ^***^ *P* < 0.001. NC, negative control.

We then went to investigate the role of CD27-AS1-208 in melanoma progression. To this end, the knockdown of CD27-AS1-208 was obtained using small interfering RNA (siRNA) in both A2058 and A375 melanoma cell lines, which was determined by qRT-PCR after 48 hours of siRNA transfection (**Figure 3A**). As was revealed by CCK8 assay, the knockdown of CD27-AS1-208 had no significant influence on short-term cell proliferation as compared to control (**Figure S7**), whereas the capacity of colony formation was markedly suppressed (**Figure 3B**). In addition, wound healing and matrigel invasion assays also revealed that the knockdown of CD27-AS1-208 significantly inhibited the migratory and invasive capacity of melanoma cells in both A2058 and A375 cell lines (**Figures 3C, D**). Taken together, these data implies that CD27-AS1-208 promotes cell proliferation, migration and invasion in melanoma.

### CD27-AS1-208 Regulates STAT3 Signaling and PP2A Activity in Melanoma Cells

**Figure 4:**
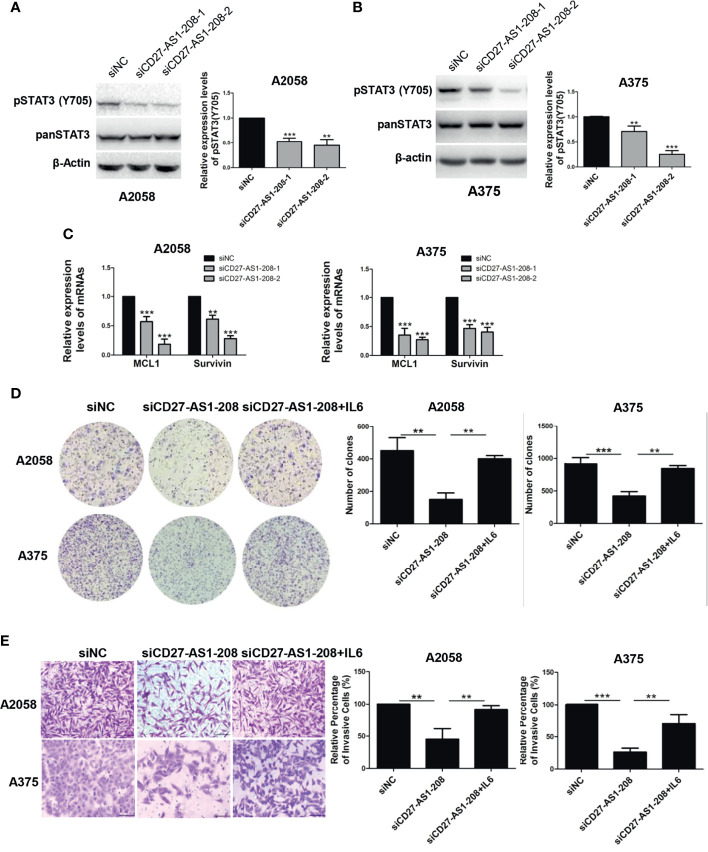
The facilitative role of CD27-AS1-208 depends on the activation of STAT3 pathway. **(A, B)** The expression levels of pSTAT3(Y705) in A2058 and A375 cell lines after 48h of transfection. **(C)** The mRNA levels of the target genes of the STAT3 pathway were detected by qRT-PCR after 48h of transfection. **(D)** Colony formation assays of A2058 and A375 cells transfected with the indicated siRNA alone or in combination with IL-6 (20ng/ml). **(E)** The invasive capacity of A2058 and A375cells transfected with the indicated siRNA alone or in combination with IL-6 (20ng/ml) was assessed by matrigel invasion assay. Scale bar = 100μm. Data represent the mean ± SD of triplicates. ^**^ *P* < 0.01, ^**^ *P* < 0.001, ***P < 0.001. NC, negative control.

Subsequently, we aimed to figure out the mechanism underlying the role of CD27-AS1-208 in melanoma progression. Regarding its nuclear localization, CD27-AS1-208 might play a role in transcriptional regulation or chromatin interactions (34). We performed RNA pull-down accompanied with mass spectrometry assay to identify CD27-AS1-208-interacting proteins in melanoma cells. The full length of CD27-AS1-208 was required to conduct RNA pull-down assay. Using the 5’ and 3’ RACE (Rapid amplification of cDNA ends) assay, we discovered that CD27-AS1-208 was a 1400-nt transcript and was poly (A) positive. The sequence of full-length CD27-AS1-208 was presented in **Table S4** and has been deposited in GenBank (GenBank accession number: MZ869025). **Figure S8A** showed a protein band in the A2058 pull-down samples. Mass spectrometry assays displayed that 854 and 128 potential interacting proteins were obtained based on confidence score >95 by CD27-AS1-208 or the antisense (AS) RNA of CD27-AS1-208 (CD27-AS1-208-AS was used as a negative control), respectively (**Figure S8B**). There were 745 proteins identified in the protein complexes pulled down by CD27-AS1-208 but not in those pulled down by CD27-AS1-208-AS (**Figure S8B**). Among them, 369 proteins located in the nucleus and they were more likely to be the candidates interacting with CD27-AS1-208 duo to their nuclear localization (**Figure S8C**). Among the candidate proteins, we selected those proteins that have been reported to regulate melanoma growth and metastasis previously for further validation and functional studies (10–18, 35–37) (**Table S5**). The relevant coverage information of these candidates in mass spectrometry analysis was listed in Table S6. Western blot results showed that CD27-AS1-208 knockdown significantly attenuated the phosphorylation of STAT3 (Tyr 705) while there were no obvious effects on the expression or activation of other candidate proteins (**Figures 4A, B** and **S8D**). Additionally, the mRNA levels of the target genes of the STAT3 pathway were also decreased by CD27-AS1-208 knockdown (**Figure 4C**). Meanwhile, we found that PP2A activity was remarkably enhanced after CD27-AS1-208 knockdown while RAC1 activity was unchanged (**Figures S8E, F**). Collectively, these results proved the regulatory role of CD27-AS1-208 in STAT3 signaling and PP2A activity in melanoma cells, implying that CD27-AS1-208 might exert its facilitative role through STAT3 pathway or/and PP2A activity.

### The Facilitative Role of CD27-AS1-208 Depends on the Activation of STAT3 Pathway

To test the involvement of PP2A, melanoma cells with or without the knockdown of CD27-AS1-208 were treated with or without okadaic acid (OA). OA is a serine/threonine protein phosphatase inhibitor that specifically blocks PP1 and PP2A activity and it is more potent against the latter, completely inhibiting PP2A at 1 nM compared with PP1 at 1 μM (37). As was shown, OA at the concentration of 1 nM had no influence on the cell vitality of A2058 and A375 cells (**Figures S9A, B**), so we chose this concentration in subsequent rescue experiments. OA treatment didn’t reverse the suppression of colony formation and tumor cell invasion caused by the knockdown of CD27-AS1-208 in both A2058 and A375 cells (**Figures S9C, D**). These results indicated that PP2A was not responsible for the effect of CD27-AS1-208 on melanoma progression.

To test whether the activation of STAT3 mediated the role of CD27-AS1-208 in melanoma progression, melanoma cells with or without the knockdown of CD27-AS1-208 were treated with or without interleukin-6 (IL-6) that is among the most crucial STAT3 activator (9). We observed that IL-6 at the concentrations from 10 to 100 ng/ml obviously enhanced pSTAT3(Y705) phosphorylation in both A2058 and A375 cell lines (**Figure S10**). As expected, IL-6 treatment could significantly rescue CD27-AS1-208 knockdown-induced decrease of colony formation and invasion in melanoma cells (**Figures 4D, E**). Altogether, these results demonstrated that CD27-AS1-208 exerted its facilitative role in melanoma progression by activating STAT3.

### CD27-AS1-208 Promotes Melanoma Growth *In Vivo*

**Figure 5:**
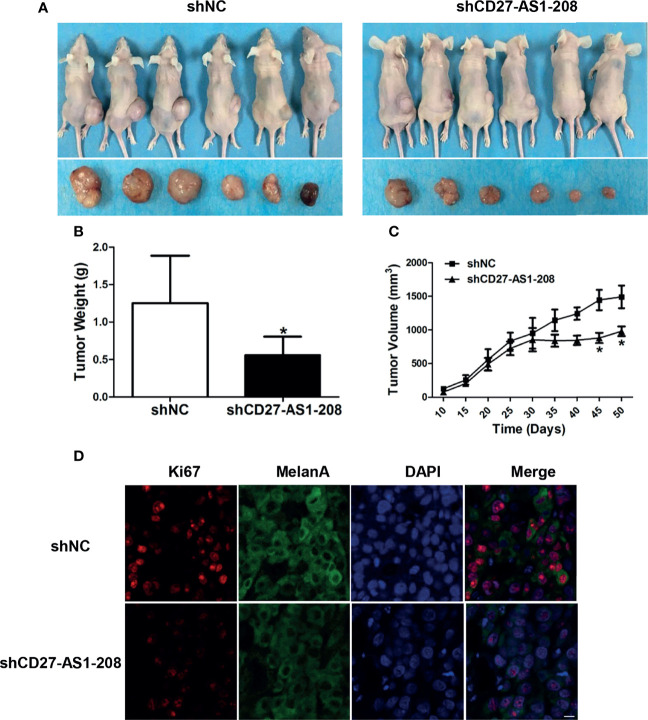
Knockdown of CD27-AS1-208 suppresses the growth of melanoma xenografts. **(A)** Photographs of mice and xenograft tumors of A2058 cells with indicated treatment after 50 days of implantation. **(B)** Tumor weight of mice was calculated 50 days after implantation. **(C)** Growth curves of xenograft tumors with indicated treatment. **(D)** Immunofluorescence staining analysis of Ki-67 in isolated tumors with indicated treatment. ^*^ *P* < 0.05; Scale bar = 10μm.

Ultimately, pre-clinical xenograft tumor model showed that the knockdown of CD27-AS1-208 expression resulted in significant suppression of tumor growth compared with the control, as revealed by impaired volume and weight of CD27-AS1-208- knockdown tumors (**Figures 5A–C**). Concurrent Ki-67 staining also showed deficient staining intensity in CD27-AS1-208- knockdown tumors compared with control (**Figure 5D**). Therefore, CD27-AS1-208 plays a prominent role in contributing to melanoma growth *in vivo*.

## Discussion

In the present study, we found that the expression profile of lncRNAs was dys-regulated in primary melanoma compared with nevus. Then CD27-AS1-208 was revealed to be up-regulated in melanoma and could be considered as a valuable indicator of melanoma progression. Subsequent *in vitro* functional and mechanistic studies showed that CD27-AS1-208 promoted melanoma cell proliferation, migration and invasion by directly interacting and activating STAT3. Furthermore, our data proved that CD27-AS1-208 contributed to melanoma growth *in vivo*. In aggregate, CD27-AS1-208 is a novel facilitator in melanoma progression *via* the activation of STAT3 pathway. Targeting CD27-AS1-208 in melanoma cells could be employed as a potential therapeutic approach.

Growing evidence has revealed that lncRNAs play crucial roles in the regulation of melanoma pathogenesis. To be specific, XIST, which is a lncRNA located on the X chromosome, is highly expressed and contributes to the progression of melanoma by sponging miRNA-23a-3p and indirectly targeting GINS2 (38). In addition, FUT8-AS1 functions as a tumor suppressor and inhibits proliferation, migration, and invasion of melanoma cells *via* binding to NF90, resulting in down-regulation of NRAS (39). Moreover, TTN-AS1 is also highly expressed in melanoma tissues. Its high expression is correlated with poor overall survival (OS) of melanoma patients and promotes melanoma cell proliferation, migration and tumor progression *in vivo* by increasing the transcription of TTN (40). Combined with these previous reports, our data forwardly highlights the great implication of lncRNAs in melanoma pathogenesis, suggesting that targeting lncRNAs is promising for treating melanoma.

Numbers of melanoma-associated lncRNAs were uncovered by multiple high-throughput analysis (41–44). Lessard et al. assessed the copy number status of intergenic domains in metastatic melanoma sample and then uncovered CASC15 was up-regulated and highly correlated with melanoma progression (43). Through analyzing the Cancer Genome Atlas (TCGA) dataset and Gene Expression Omnibus (GEO) dataset, Chen et al. identified a four-lncRNAs prognostic signature with the ability of risk stratification for melanoma patients (41). Nevertheless, the difference of lncRNAs expression profile between primary melanoma and nevus was unknown. Herein, we showed that lncRNAs were dys-regulated in primary melanoma compared with nevus, further substantiating the pathogenic role of lncRNAs in melanoma development.

Among these dys-regulated lncRNAs in our microarray data, several have been proved to be vital for melanoma initiation and progression. ANRIL and CASC15, which were documented to be overexpressed and had oncogenic role in melanoma (43, 45), were also up-regulated in primary melanoma in our microarray data. LncRNA PTENP1 was a crucial negative mediator of PTEN and was frequently deleted in 14-21% of melanoma (46). In parallel, we observed decreased expression of PTENP1 in primary melanoma compared with nevus. These results forwardly supported the relatively high credibility and repeatability of previous investigations and our microarray data, demonstrating the stability of detecting dys-regulated lncRNAs in different systems. Whether the above-mentioned lncRNAs could be employed as potential biomarker for predicting melanoma progression and prognosis needs further validation in a larger cohort.

Eradication of immune destruction is a hallmark of cancer including melanoma. Of note, some lncRNAs have been found to be the key regulators in cancer immunity (47). Lnc-CHOP has an important role in controlling the immunosuppressive function of myeloid-derived suppressor cells (MDSCs) in tumor microenvironment (48). LINK-A enhances the degradation of the antigen peptide-loading complex (PLC) to suppress tumor immunosurveillance in breast cancer (49). Previously, Ping et al. has identified 17 pairs of co-expressed and immune-related lncRNAs that could divide melanoma cohort into high-risk and low-risk groups. For instance, the co-expression of MIR205HG and U62631.1 lncRNA pair is correlated with high risk and the co-expression of HLA-DQB1-AS1 and UBA6-AS1 is linked to the protective effects in melanoma (50). In addition, Wang et al. have identified eight immune-related lncRNAs with prognostic value in melanoma dataset. Among them, MIR205HG expression is associated with the poor outcome of patient and its expression is positively correlated with the expressions of immune checkpoints like PD-1, CTLA4, LAG3, and TIM3 in adenocarcinoma and melanoma (51). Our present study demonstrates that immunology-related pathway including innate and adaptive immune response were among the most significantly-enriched biological processes of dys-regulated lncRNAs in primary melanoma, implying that dys-regulated lncRNAs may participate in the regulation of anti-tumor immunity and inflammatory responses during melanoma progression. The role of tumorous lncRNAs in the regulation of tumor immunology warrants further investigations.

Our subsequent results revealed that CD27-AS1-208 was up-regulated in primary melanoma compared with nevus and its expression was positively correlated with melanoma progression. Further *in vitro* and *in vivo* study confirmed the facilitative role of CD27-AS1-208 in melanoma growth and progression. The biological function of CD27-AS1-208 has not been characterized until the present study. It is one of the transcripts of CD27-AS1, which is reported to be an unfavorable prognostic factor as its up-regulation has been documented to be significantly correlated with poorer survival of patients with colon adenocarcinoma or acute myeloid leukemia (AML) (52, 53). Therefore, both our data and previous reports have illustrated the great potential of CD27-AS1 as a valuable therapeutic target.

Cumulating evidence has proved the vital role of STAT3 activation in melanoma initiation and progression (10–18). Congruently, JAK-STAT signaling was among the most significantly enriched pathways of dys-regulated lncRNAs, which indicated the modulatory effect of lncRNAs on STAT signaling in melanoma. Specifically, we discovered that CD27-AS1-208 up-regulation promoted STAT3 pathway, contributing to melanoma growth and progression. Several compounds that inhibit either the function or expression of STAT3 have now been processed to clinical trials (9). Thus, our data raise the possibility that CD27-AS1-208 could be employed as a target for inhibition of STAT3 signaling for melanoma treatment.

To date, extensive mechanisms reportedly underlie the regulatory role of lncRNAs in STAT3 pathway (54). Many lncRNAs function as a competing endogenous RNA (ceRNA) to increase STAT3 expression or activity by sponging miRNAs (55–58). Lnc-DC promotes STAT3 signaling by interacting with the c-terminus of STAT3 to prevent dephosphorylation of STAT3 Y705 by SHP1 (59). GAS5 accelerates the degradation of STAT3 *via* promoting TRAF6-mediated ubiquitination (60). We found that CD27-AS1-208, which located in the nucleus, interacted with STAT3 and positively regulated STAT3-Tyr705 phosphorylation while the total level of STAT3 was not affected. Therefore, it is possible that CD27-AS1-208 may enhance or maintain the phosphorylation level of STAT3-Tyr705 in the nucleus *via* preventing dephosphorylation of STAT3 Y705, which would be clarified further in our future study.

In conclusion, our study demonstrates that the dys-regulation of lncRNAs is greatly involved in the pathogenesis of melanoma. These lncRNAs may be a group of potential regulators implicated in melanomagenesis *via* the regulation of melanoma biology and the immune response. Moreover, we have demonstrated that lncRNA CD27-AS1-208 was significantly up-regulated and exerted a facilitative role in melanoma progression through the activation of STAT3 pathway. Further validations in larger cohorts are needed to confirm the great value of CD27-AS1-208 in the treatment of melanoma.

## Data Availability Statement

The lncRNAs microarray data have been deposited in GEO (accession number: GSE183878). The sequence of CD27-AS1-208 has been deposited in GenBank (accession number: MZ869025). Datasets analyzed in this article can be found at TCGA database.

## Ethics Statement

The studies involving human participants were reviewed and approved by The Ethics Review Board of Fourth Military Medical University. The patients/participants provided their written informed consent to participate in this study. The animal study was reviewed and approved by Subcommittee on Research Animal Care of the Fourth Military Medical University.

## Author Contributions

CL, WG, and JM conceived the experiments. SG and QS analyzed the data. JM, QS, SG, PX, and WZ performed experiments. QY, XY, and LL did the animal study. YL, TZ, and YY provided tissue samples. JM, WG, CL, QS, and TG wrote the paper. All authors contributed to the article and approved the submitted version.

## Funding

The research leading to these results has received funding from National Natural Science Foundation of China (No. 81402736, No. 81902791) and Shaanxi Young Talents Program (20200303), Support Program of Young Talents in Shaanxi Province (No. 20200303) and Young Eagle Project of Fourth Military Medical University (No. 2019cyjhgwn).

## Conflict of Interest

The authors declare that the research was conducted in the absence of any commercial or financial relationships that could be construed as a potential conflict of interest.

## Publisher’s Note

All claims expressed in this article are solely those of the authors and do not necessarily represent those of their affiliated organizations, or those of the publisher, the editors and the reviewers. Any product that may be evaluated in this article, or claim that may be made by its manufacturer, is not guaranteed or endorsed by the publisher.
