# Supplementary material for: Long Non-Coding RNA CD27-AS1-208 Facilitates Melanoma Progression by Activating STAT3 Pathway
Source: Front Oncol. 2022 Jan 13;11:818178. doi: 10.3389/fonc.2021.818178 (PMC8791859; doi:10.3389/fonc.2021.818178)
Supplement: Supplementary file 1 [file DataSheet_1.docx]

**SUPPLEMENTARY TABLES**

**Supplementary Table 1 Sample information of Nevus and primary MM used for validation of microarray data**

| **Nevus** | | | **Primary MM** | | | | **Metastasis MM** | | |
| --- | --- | --- | --- | --- | --- | --- | --- | --- | --- |
| NO. | Gender | Old(y) | NO. | Gender | Old(y) | Stages | NO. | Gender | Old(y) |
| 1 | Male | 58 | 1 | Male | 48 | IIC | 1 | Female | 58 |
| 2 | Female | 32 | 2 | Male | 57 | II | 2 | Female | 30 |
| 3 | Female | 25 | 3 | Male | 36 | II | 3 | Male | 59 |
| 4 | Female | 25 | 4 | Male | 59 | II | 4 | Male | 46 |
| 5 | Female | 50 | 5 | Male | 53 | II | 5 | Female | 55 |
| 6 | Female | 36 | 6 | Female | 64 | II | 6 | Female | 64 |
| 7 | Male | 10 | 7 | Female | 49 | II | 7 | Female | 36 |
| 8 | Male | 9 | 8 | Male | 65 | IIC | 8 | Male | 66 |
| 9 | Male | 5 | 9 | Female | 51 | IIB | 9 | Male | 59 |
| 10 | Male | 19 | 10 | Female | 42 | IIC | 10 | Male | 59 |
| 11 | Male | 15 | 11 | Female | 51 | I | 11 | Male | 66 |
| 12 | Female | 44 | 12 | Male | 69 | IIC | 12 | Female | 57 |
| 13 | Female | 33 | 13 | Female | 66 | IIC | 13 | Male | 58 |
| 14 | Female | 25 | 14 | Female | 42 | IIC | 14 | Female | 40 |
| 15 | Male | 6 | 15 | Female | 48 | II | 15 | Male | 16 |
| Abbreviation: No., number; MM, melanoma. | | | | | | | | | |

**Supplementary Table 2** **Gene-specific primer sequences and annealing temperatures**

| **Name** | **Gene ID** | **Primer Sequence (5’-3’)** | | **Tm (℃)** |
| --- | --- | --- | --- | --- |
| LINC00518-204 | ENST00000491317.1 | Forward | GCTCAGCAGTTCCCCTCCTT | 58 |
|  |  | Reverse | AACATGGTGCCATTCAGTTATTC | 58 |
| MIAT-206 | ENST00000425476.1 | Forward | GGACAGAGGAGACAGAAGGGAAT | 58 |
|  |  | Reverse | CCTCCCACTTTGGCATTCTA | 58 |
| CHL1-AS1-201 | ENST00000417612.1 | Forward | CAAAAACCCTCCTCATTCTGTCAT | 58 |
|  |  | Reverse | GGCAGCCAGGTGTCTGTAGTG | 58 |
| novel transcript | ENST00000600152.1 | Forward | GTTTTGGTCATGGGAGGAGATT | 58 |
|  |  | Reverse | GGCAAGACAGAGAACAGGAGAGAT | 58 |
| LINC01515-201 | ENST00000433152.2 | Forward | TCATGGATTATTGTCCGAAGCA | 58 |
|  |  | Reverse | GAGGCAGGAGAATGGTGTGAAC | 58 |
| SOX21-AS1-201 | ENST00000438290.1 | Forward | ACTGTGAACAGCAGTGGCATCT | 58 |
|  |  | Reverse | CAGAGCCTGTAAAGGAATATGGTT | 58 |
| CD27-AS1-208 | ENST00000538616.1 | Forward | CCCTCCTCTTATCCCTAATCCTA | 58 |
|  |  | Reverse | ATGCAGACTCTGGCTGACTTCA | 58 |
| novel transcript | ENST00000607434.1 | Forward | GGTGGAAGGATTTGCTTGGTAA | 58 |
|  |  | Reverse | GAGCGAGTGCCATACGTCATC | 58 |
| MCL1 | 4170 | Forward | TCATTTCTTTTGGTGCCTTTGT | 58 |
|  |  | Reverse | AGCCAGTCCCGTTTTGTCCT | 58 |
| Survivin | 332 | Forward | CGAACCCCAGACCTGTTTGTAT | 58 |
|  |  | Reverse | CTGGAGTGCATTTTCTGCTAACA | 58 |
| β-actin | NM_001101 | Forward | AGAAAATCTGGCACCACACC | 58 |
|  |  | Reverse | AGAGGCGTACAGGGATAGCA | 58 |

| **Supplementary Table 3** **Co-expression of lncRANs with immune-related mRNAs** | | | | |
| --- | --- | --- | --- | --- |
| **LncRNA ID** | **mRNA ID** | **mRNA.GeneSymbol** | **Correlation** | **P.value** |
| ENST00000418945.1 | NM_004131 | GZMB | -0.99056 | 0.000133 |
| TCONS_00016045 | NM_004131 | GZMB | -0.99391 | 5.55E-05 |
| TCONS_00015772 | NM_004131 | GZMB | 0.998642 | 2.76E-06 |
| NR_033999.1 | NM_004131 | GZMB | -0.99726 | 1.12E-05 |
| HIT000393033 | NM_004131 | GZMB | -0.99634 | 2.00E-05 |
| ENST00000414430.1 | NM_004131 | GZMB | -0.99376 | 5.82E-05 |
| TCONS_00029753 | NM_004131 | GZMB | 0.991387 | 0.000111 |
| ENST00000522646.1 | NM_004131 | GZMB | -0.99413 | 5.16E-05 |
| ENST00000551450.1 | NM_004131 | GZMB | -0.99489 | 3.91E-05 |
| ENST00000505848.1 | NM_002286 | LAG3 | -0.9914 | 0.000111 |
| ENST00000502083.2 | NM_002286 | LAG3 | 0.994619 | 4.34E-05 |
| TCONS_00016434 | NM_002286 | LAG3 | -0.99699 | 1.36E-05 |
| uc009yti.1 | NM_002286 | LAG3 | 0.992508 | 8.40E-05 |
| uc002ysp.3 | NM_002286 | LAG3 | -0.99004 | 0.000148 |
| HIT000248864 | NM_002286 | LAG3 | -0.99556 | 2.95E-05 |
| ENST00000502049.2 | NM_002286 | LAG3 | -0.99006 | 0.000148 |
| ENST00000419650.1 | NM_002286 | LAG3 | -0.99342 | 6.49E-05 |
| RNA33446 | NM_002286 | LAG3 | 0.993632 | 6.07E-05 |
| ENST00000505848.1 | NM_005018 | PD-1 | -0.99434 | 4.79E-05 |
| ENST00000441399.1 | NM_005018 | PD-1 | -0.99136 | 0.000112 |
| TCONS_00012428 | NM_005018 | PD-1 | -0.99438 | 4.73E-05 |
| XR_158903.2 | NM_005018 | PD-1 | -0.99733 | 1.07E-05 |
| uc009yti.1 | NM_005018 | PD-1 | 0.992396 | 8.65E-05 |
| uc002ysp.3 | NM_005018 | PD-1 | -0.99005 | 0.000148 |
| ENST00000502049.2 | NM_005018 | PD-1 | -0.99406 | 5.28E-05 |
| ENST00000462383.1 | NM_005018 | PD-1 | -0.99331 | 6.70E-05 |
| TCONS_00029753 | NM_005018 | PD-1 | 0.994934 | 3.84E-05 |
| ENST00000601007.1 | NM_005018 | PD-1 | 0.990971 | 0.000122 |
| XR_427821.1 | NM_005018 | PD-1 | -0.99879 | 2.18E-06 |
| ENST00000324348.7 | NM_005018 | PD-1 | -0.99214 | 9.23E-05 |
| ENST00000595892.1 | NM_005018 | PD-1 | 0.992864 | 7.62E-05 |
| ENST00000598546.1 | NM_005018 | PD-1 | 0.990587 | 0.000132 |
| ENST00000600008.1 | NM_005018 | PD-1 | 0.992231 | 9.03E-05 |
| ENST00000439745.1 | NM_005018 | PD-1 | -0.99195 | 9.70E-05 |
| ENST00000519624.1 | NM_005041 | PRF1 | -0.99009 | 0.000147 |
| ENST00000533002.1 | NM_005041 | PRF1 | -0.99262 | 8.15E-05 |
| uc001laz.3 | NM_005041 | PRF1 | -0.99343 | 6.47E-05 |
| ENST00000580056.1 | NM_005041 | PRF1 | 0.991551 | 0.000107 |
| ENST00000452731.1 | NM_005041 | PRF1 | -0.99213 | 9.28E-05 |

**Supplementary Table 4** **The 5’ and 3’ ends of CD27-AS1-208 were acquired by Rapid Amplification of cDNA Ends (RACE)**

| **Position** | **Sequence** |
| --- | --- |
| 5’ end | ATATTTGTCCTCGTCCCTGCCAGTCTCGAAAAGGCACTCTGTCACGTGTACACAGGAAAGGGCCCTGACAAGAGGATGGGACTGCAGTTGTGGCTGCCAGGGCCCCGGCCAGGGTGAAAACAAGGAACATTCCAGAGAAGATCACAAGGATGCGAATAAAATCGGAGCTGCACAGGGATCTTTGGGCTGTAATAGGAGGGGACACA |
| Middle | CAGGGGTTAGGGGAGGGTCTAGCCCTTGGCTCCCTCAGCCCAGCCCTCCTGTCACCTTGCCTCTCCCCTTTCCCCCACCTTATTCTATCCCACCCGCCACCGTTGGCCAACTCCTCTCCTAAACAGTGGACTCTGCTTTTTCCCCTCCTCTTATCCCTAATCCTAGGAGCTTTCTGTCTGGCTTCCCTCCTGGCCCCTCGTGTATGCATTCTCTCTAGCGGTGGGGATTAAGGAGAAAACTCACGTGGCCAGTGGGTAGAGAGAGTCCGGGCAGGCAGCTGCCTGAAGTCAGCCAGAGTCTGCATGTGCCCAGCTGTCCTGGCCTCCAGCATCTCTGGAAAAACAGAGACAGAATGAGTAGCAGCAGGGGACCCCATAGGGCACATCTGAAGGCCTGAGAGGTGCTGGATGGGATGGGAAGGCAGAAACAGGCTGACC |
| 3’ end | TTATCTAGGGGTAGGAGTGGGGAGTACTGGTTGGGGTATCACCGCTACCCCACGTGATGGCACAGAGAGTTGCCTGGAACTTACCACTGACATAAGAGTCTTGCTTTGTCGCCCAGGTTGGAGTGCAGTGGCACGATCTCGGCTCAGTGCAACCTCCGCCTCCTGAGTTCAAGTGATTCTCCTGCCTCAGCCTCCCTAGTAGCTGGGATTACAGGACAAGGAGAGGGACAAATTCCTTCTTGGCATCCTTATGGCCCTGTGACCTGCTAATGAATGCTGACTCTGAGTTGCTACTAGAATTTGGTTTCCTTGTGGCTTCCCAGGAATTGTGAGCCATGTTGGATAAGGACCTGCAAGGGAAGAATGAGGAAGCCTGGGACAGTGCAGGAGGGGAGAGCACTGGTGGTAGAGAGGAGTGATGACAAGGGTCTCCGCCAAAAGGGAAATGGAGGGGTTAAAGAAGCTGCTGGGGAGGGTTAAAAACAATGTGTTCTCACTCGTAGGTGGGAATTGAACAATGAGAACACTTGGACACAGGAAGGGGAACATCACACACTGGGGCCTGTCGTGGGGTGTGGGGAGGGAGGAGGGATAGCATTAGGAGATATACCTAATGTAAATGATGAGTTAATGGGTGCAGCACACCAACATGGCACATGTATACCTATGTAACAAACCTGCACATTGTGTACATGTACCCTAGAACTTAAAGTATAATTAAAAAACAAAACAAAACAAAACAAAAAAAAAAAAAAA |

**Supplementary Table 5** **Candidate proteins selected for validation**

| **Protein** | **Name** | **Function or Pathway** |
| --- | --- | --- |
| **PP2CA** | Protein phosphatase 2CA | The catalytic subunit of the major Ser/Thr phosphatases 2A (PP2A) |
| **MAPK1** | Mitogen-activated protein kinase 1 | Also known as ERK in MAPK pathway |
| **PHB** | Prohibitin | Function as a trans-acting regulatory RNA |
| **STAT3** | Signal transducer and activator of transcription 3 | A nuclear transcription factor of STAT family |
| **RAC1** | Rac family small GTPase 1 | A GTPase which belongs to the RAS superfamily of small GTP-binding proteins |

| **Supplementary Table 6 Relevant coverage information of candidate proteins**  **pull-down by CD27-AS1-208** | | | | |
| --- | --- | --- | --- | --- |
| **Protein** | **Name** | **Mass (Da)** | **Coverage (%)** | **Unique Peptide** |
| **PP2CA** | Protein phosphatase 2CA | 88067.2 | 8.31 | 4 |
| **MAPK1** | Mitogen-activated protein kinase 1 | 29803.8 | 25.37 | 6 |
| **PHB** | Prohibitin | 35574.8 | 6.15 | 2 |
| **STAT3** | Signal transducer and activator of transcription 3 | 21449.9 | 11.98 | 1 |
| **RAC1** | Rac family small GTPase 1 | 42089.3 | 4.93 | 2 |

**SUPPLEMENTARY FIGURE LEGENDS**

**Supplementary Figure 1.** **Characterization of lncRNAs and mRNAs expression profiles in primary melanoma compared with nevus.** (A) Volcano plots show significantly differentially-expressed lncRNAs and mRNAs in primary MM compared with nevus. The values of X axis is log2 (fold change) and Y axis is -log10 (*P* value). The red and green points in the plot represent the significantly high and low expressed-lncRNAs or mRNAs respectively. (B) Hierarchical clustering results of differentially-expressed mRNAs in primary MM and Nevus. “Green” indicates relative low expression whereas “red” indicates relative high expression. (C) Chromosome distribution of differentially-expressed lncRNAs. The x-axis shows the number of lncRNAs. MM, melanoma.

**Supplementary Figure 2. Bioinformatics analysis of differentially-expressed mRNAs in primary MM and Nevus.** (A) GO enrichment analysis was performed on biological processes. (B) Results of KEGG pathway analysis. The vertical and horizontal axis represent the biological process or pathways and −log10 (*P* value) of the corresponding biological process or pathways, respectively. The top 10 significant GO items and pathways are shown. MM, melanoma; Sig, significant.

**Supplementary Figure 3. CD27-AS1-208 is highly expressed in the tumors of CHOL, DLBC, HNSC, KIRP, LAML, PAAD, PCPG and THYM.** The expression level of CD27-AS1-208 in other kinds of tumors was explored by analyzing TCGA (The Cancer Genome Atlas) database. TPM represents relative expression level. CHOL, Cholangio carcinoma; DLBC, Lymphoid Neoplasm Diffuse Large B-cell Lymphoma; HNSC, Head and Neck squamous cell carcinoma; KIRP, Kidney renal papillary cell carcinoma; LAML, Acute Myeloid Leukemia; PAAD, Pancreatic adenocarcinoma; PCPG, Pheochromocytoma and Paraganglioma; THYM, Thymoma.

**Supplementary Figure 4. CD27-AS1-208 is mainly located in the nucleus.** The expression level of CD27-AS1-208 in cytoplasm and nucleus of A2058 and 451Lu cells was tested by semi-quantitative PCR.

**Supplementary Figure 5. The representative images of high and low CD27-AS1-208 expression in primary melanoma.** Fluorescence in situ hybridization (FISH) analysis was performed to show the expression and location of CD27-AS1-208 in primary melanoma. A and B are representative images of high and low CD27-AS1-208 expression, respectively.

**Supplementary Figure 6. The correlation of CD27-AS1-208 level with the expression of MITF, TYR and PMEL in melanoma.** TCGA Skin Cutaneous Melanoma (SKCM) database was used to analyze the correlation of CD27-AS1-208 level with the expression of MITF, TYR and PMEL in melanoma. TPM represents relative expression level.

**Supplementary Figure 7. Knockdown of CD27-AS1-208 has no effect on the short term proliferation of melanoma cells.** The proliferative ability of A2058 and A375 cells was measured by CCK8 assay at different time points after cells were transfected with siCD27-AS1-208 or siNC. Data represent the mean ± SD of triplicates. NC, negative control.

**Supplementary Figure 8. Identification of protein regulated by lncRNA CD27-AS1-208 in A2058 cell.** (A) The protein interacting with lncRNA CD27-AS1-208 were acquired using RNA pull-down and silver-stained as indicated by red box. (B) The venn diagram of protein interacting with lncRNA CD27-AS1-208 and the control. Red marks indicate proteins interacting with CD27-AS1-208-sense whereas blue indicates proteins interacting with CD27-AS1-208- antisense as control. (C) The number of cytoplasmic and nuclear protein interacted with CD27-AS1-208. (D) The expression levels of indicated protein after 48h of transfection. (E and F) The activities of PP2A and RAC1 were measured after 48h of transfection. Data represent the mean ± SD of triplicates. ^*^, *P*<0.05，^**^, *P*<0.01; NC, negative control; ns, not significant.

**Supplementary Figure 9. Inhibition of PP2A activity has no effect on the anti-tumor function of the knockdown of CD27-AS1-208 in melanoma cells.** (A and B) Cell viability of A2058 and A375 cells at different time points after indicated treatment. (C) Colony formation of A2058 and A375 cells transfected with indicated siRNA alone or in combination with OA (1nM). The column represents the number of colonies. (D) The invasive capacity of A2058 and A375 cells transfected with the indicated siRNA alone or in combination with OA (1nM) was assessed by matrigel invasion assay. Representative fields of the invaded cells are shown on the left. The invaded cells were quantified on the right. Scale bar = 100μm. Data represent the mean ± SD of triplicates. ^*^, *P*<0.05, ^***^, *P*<0.001. NC, negative control.

**Supplementary Figure 10. STAT3 signaling is activated by IL-6 in melanoma cells.** The activation of STAT3 (pSTAT3(Y705)) was tested by western blot in A2058 and A375 cells after indicated treatment.
