# Supplementary figures and images for: Long Non-Coding RNA CD27-AS1-208 Facilitates Melanoma Progression by Activating STAT3 Pathway (part 1 of 2)
Source: Front Oncol. 2022 Jan 13;11:818178. doi: 10.3389/fonc.2021.818178 (PMC8791859; doi:10.3389/fonc.2021.818178)

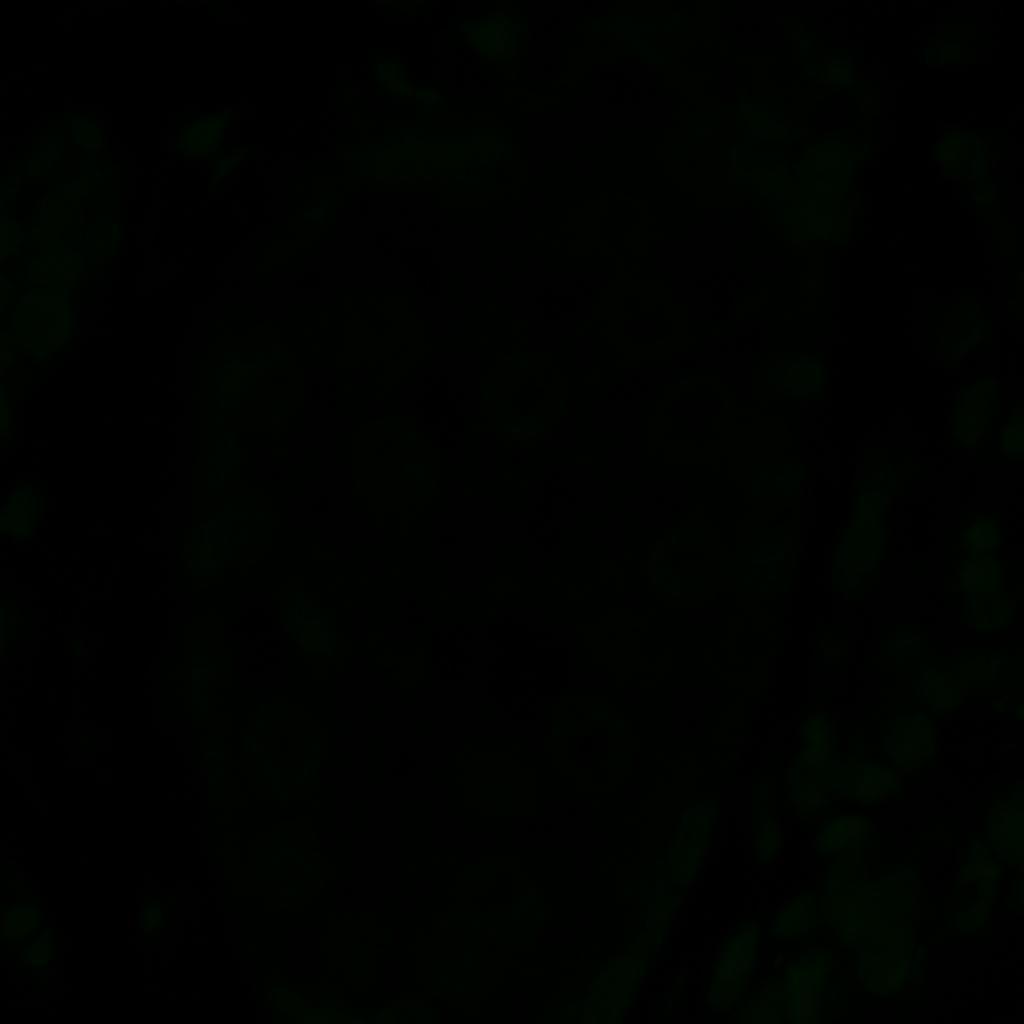

Supplement: Supplementary file 2 [file DataSheet_2.zip › ROW DATA Figure 1-2/Figure 2/Figure 2E/Nevus/CD27-AS1-208.jpg]

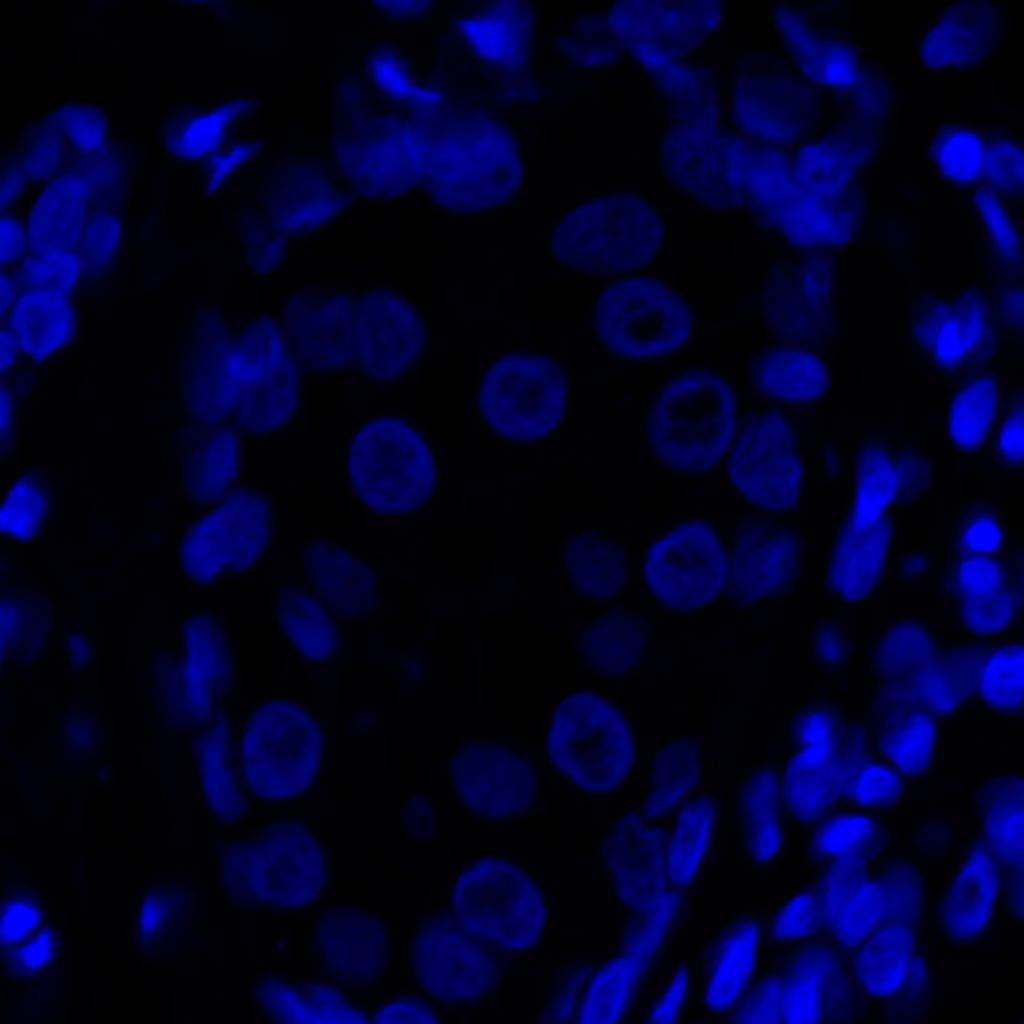

Supplement: Supplementary file 2 [file DataSheet_2.zip › ROW DATA Figure 1-2/Figure 2/Figure 2E/Nevus/DAPI.jpg]

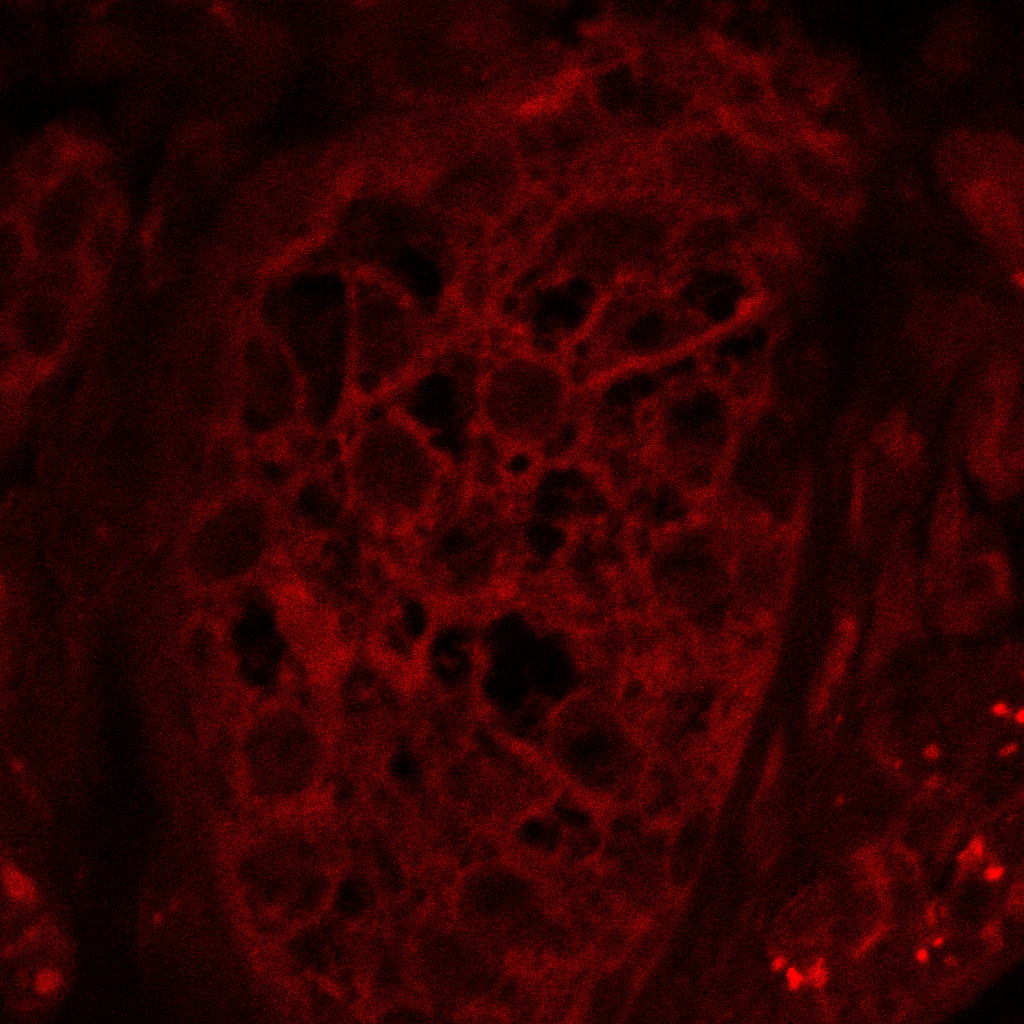

Supplement: Supplementary file 2 [file DataSheet_2.zip › ROW DATA Figure 1-2/Figure 2/Figure 2E/Nevus/Melan A.jpg]

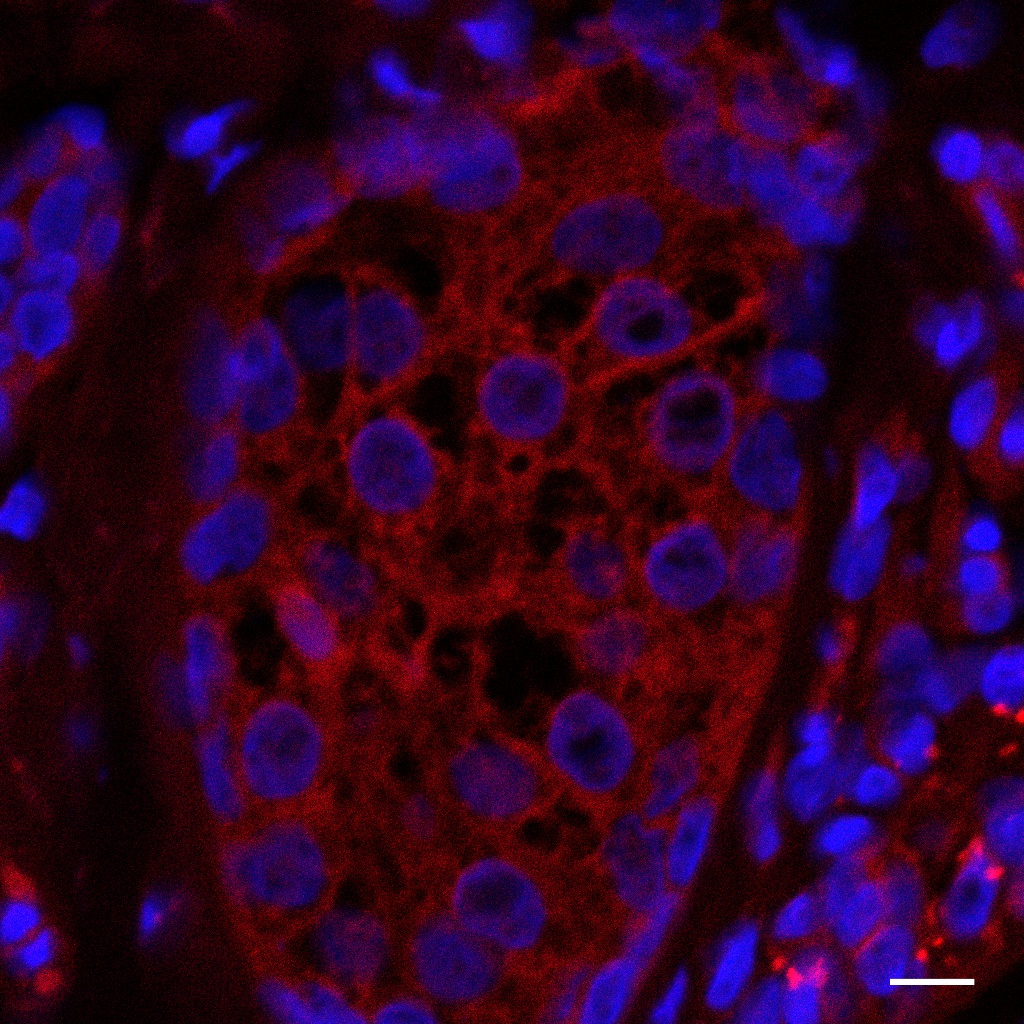

Supplement: Supplementary file 2 [file DataSheet_2.zip › ROW DATA Figure 1-2/Figure 2/Figure 2E/Nevus/Merge.jpg]

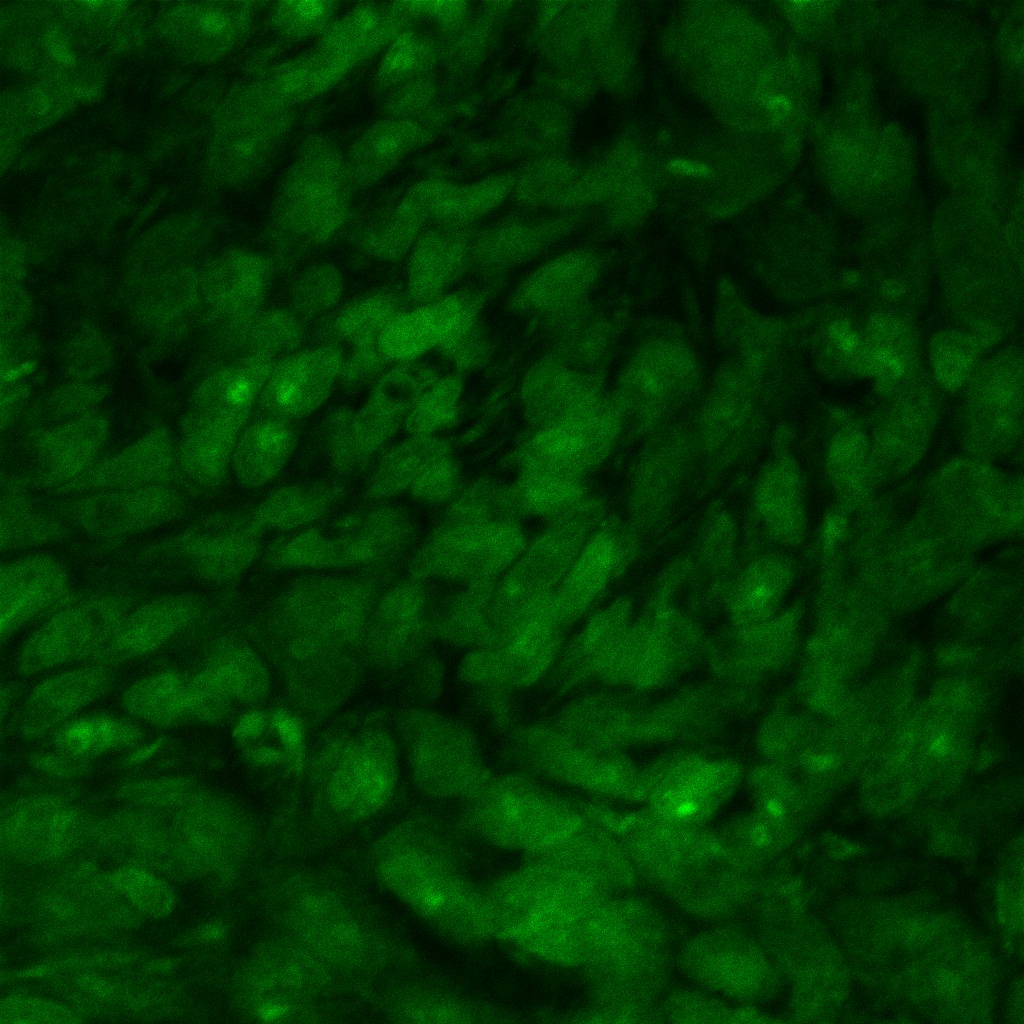

Supplement: Supplementary file 2 [file DataSheet_2.zip › ROW DATA Figure 1-2/Figure 2/Figure 2E/Primary melanoma/CD27-AS1-208.jpg]

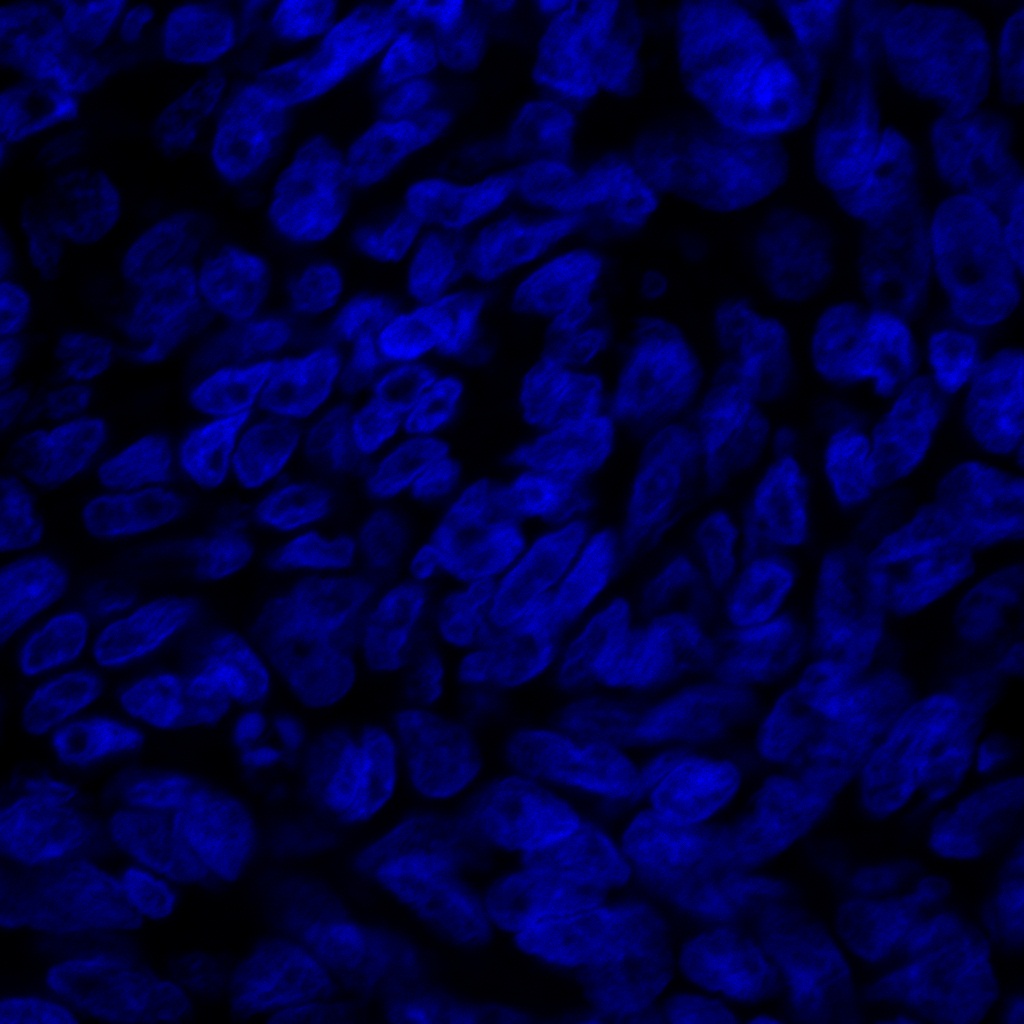

Supplement: Supplementary file 2 [file DataSheet_2.zip › ROW DATA Figure 1-2/Figure 2/Figure 2E/Primary melanoma/DAPI.jpg]

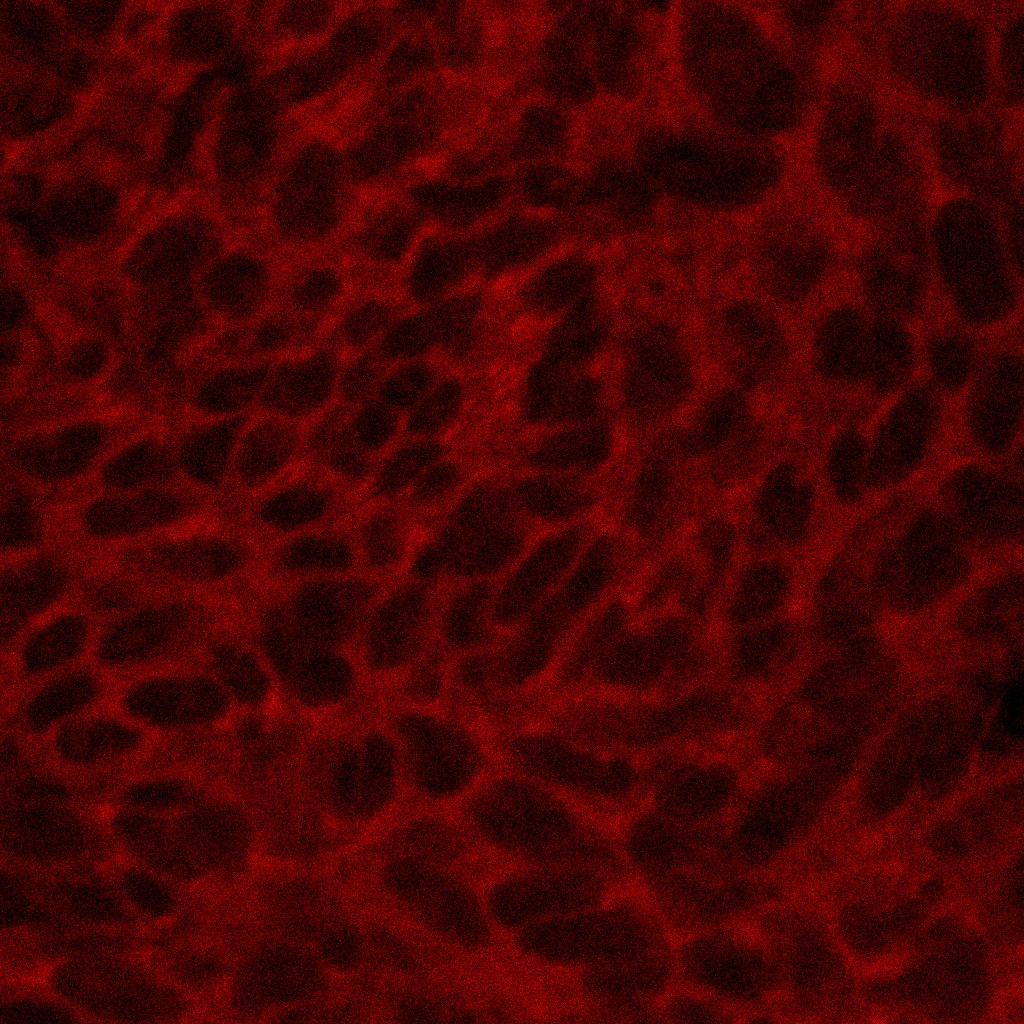

Supplement: Supplementary file 2 [file DataSheet_2.zip › ROW DATA Figure 1-2/Figure 2/Figure 2E/Primary melanoma/Melan A.jpg]

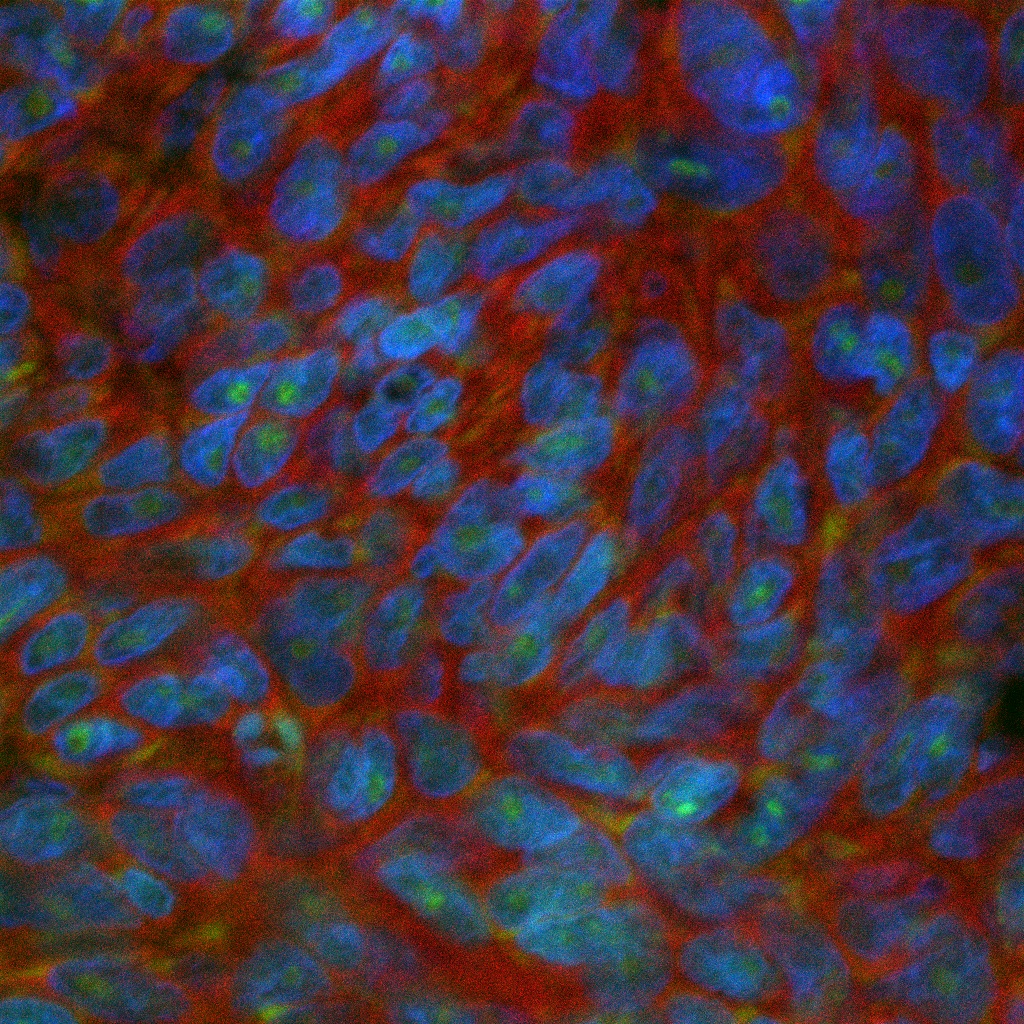

Supplement: Supplementary file 2 [file DataSheet_2.zip › ROW DATA Figure 1-2/Figure 2/Figure 2E/Primary melanoma/Merge.jpg]

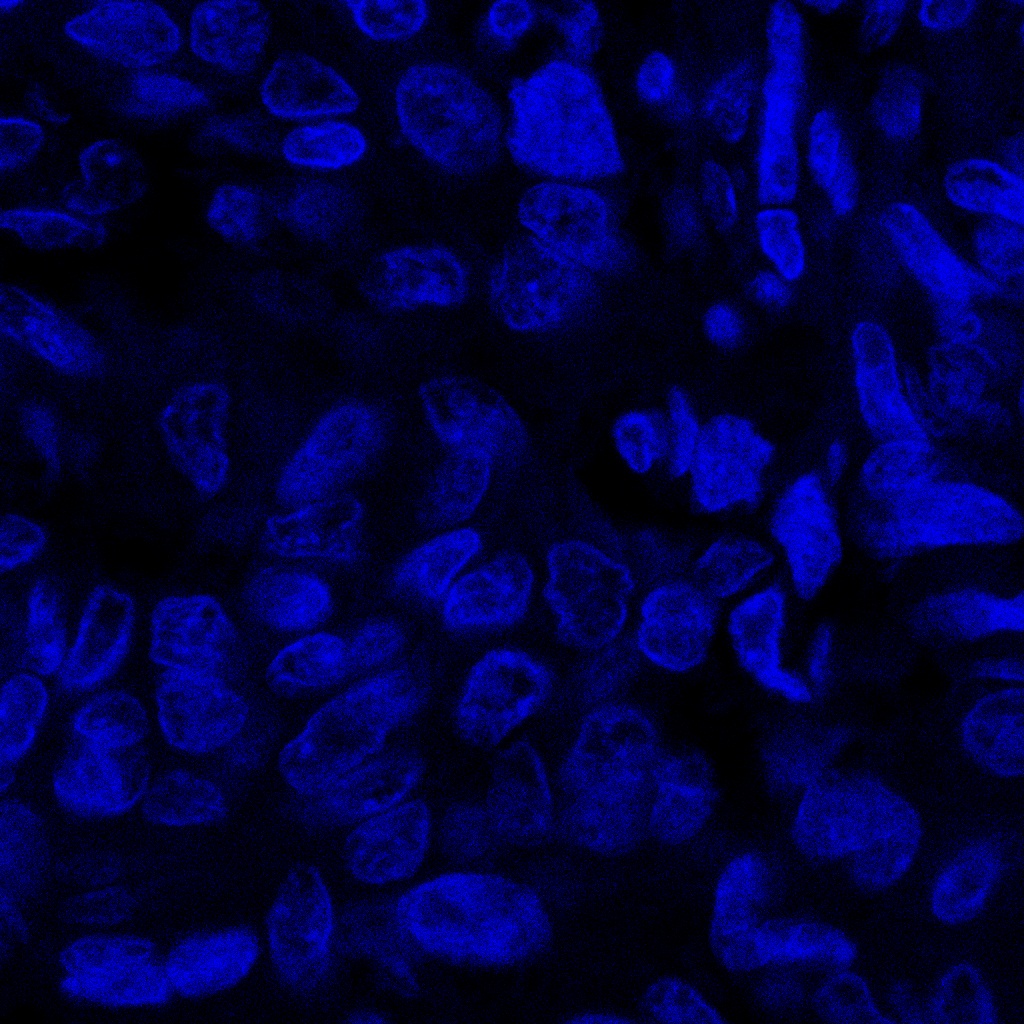

Supplement: Supplementary file 2 [file DataSheet_2.zip › ROW DATA Figure 1-2/Figure 2/Figure 2F/High/DAPI.jpg]

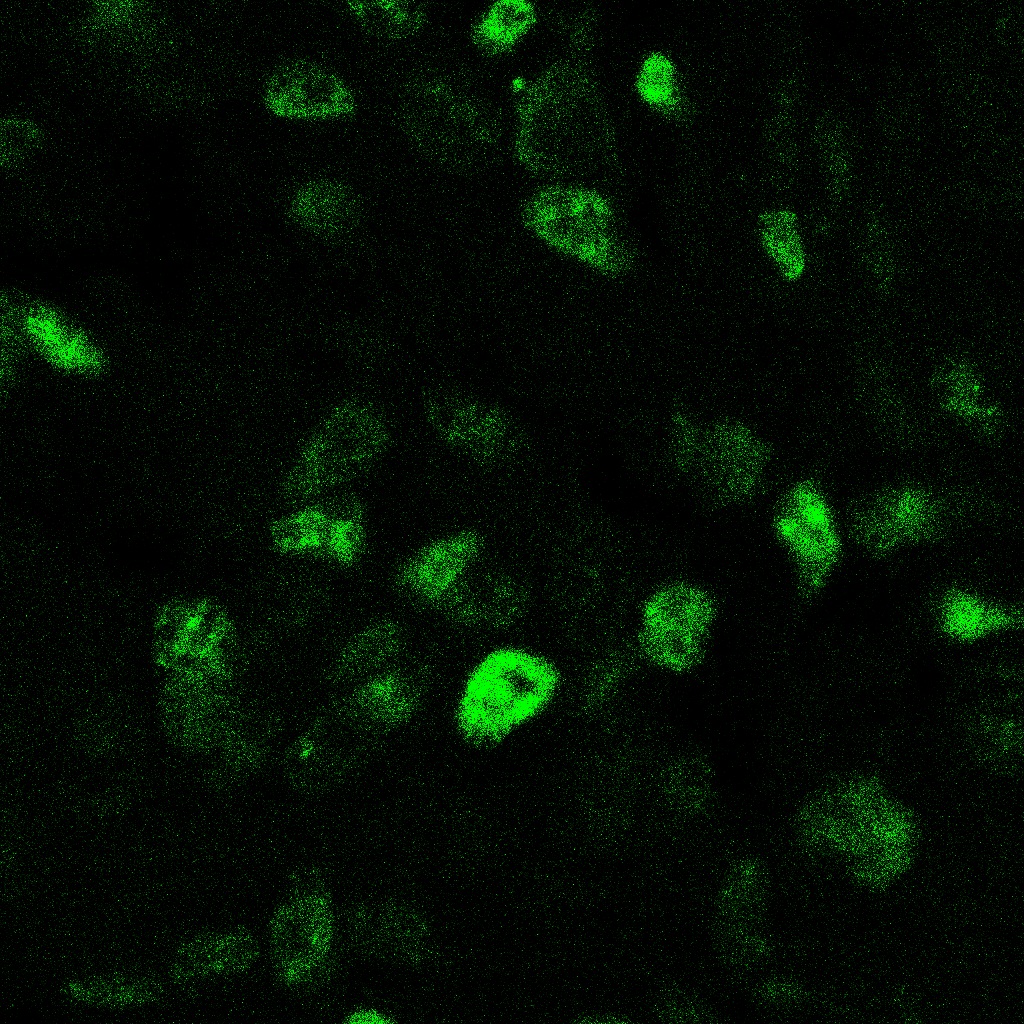

Supplement: Supplementary file 2 [file DataSheet_2.zip › ROW DATA Figure 1-2/Figure 2/Figure 2F/High/Ki67.jpg]

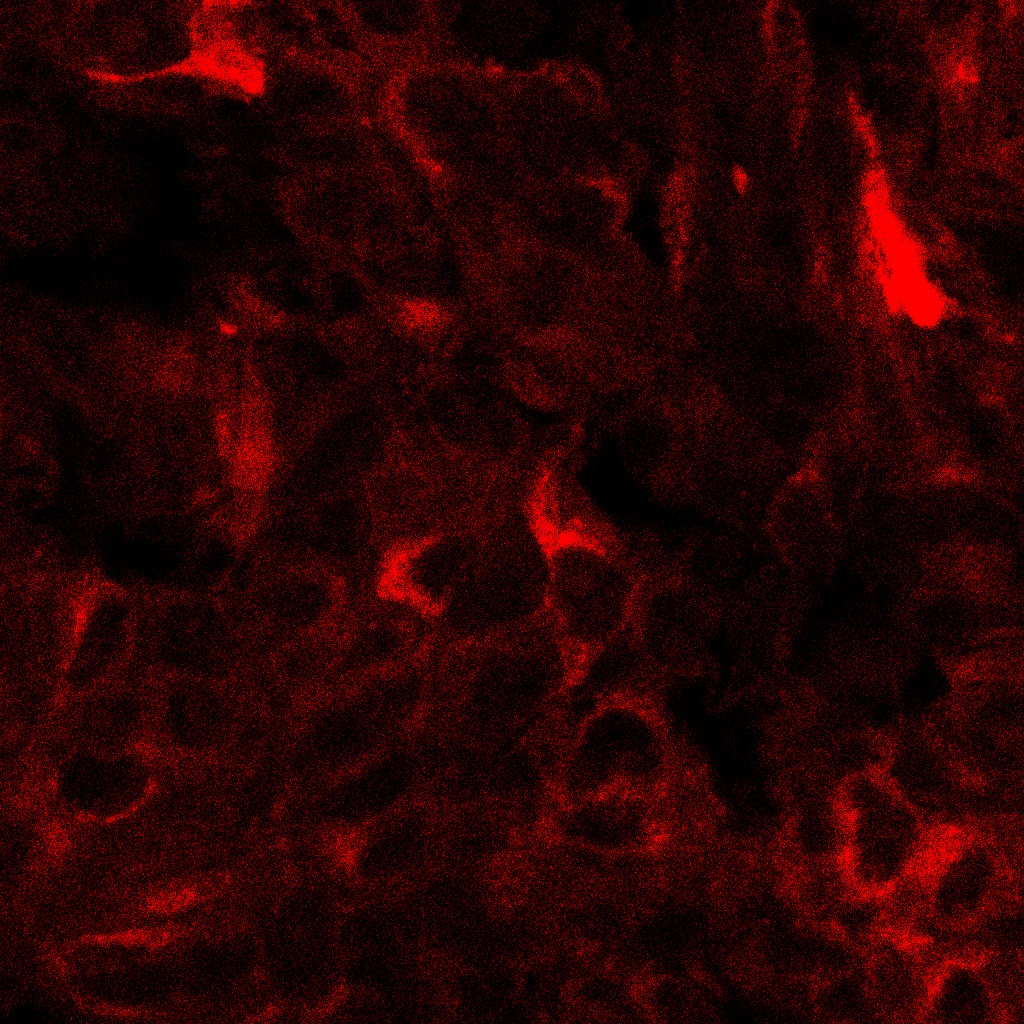

Supplement: Supplementary file 2 [file DataSheet_2.zip › ROW DATA Figure 1-2/Figure 2/Figure 2F/High/Melan A.jpg]

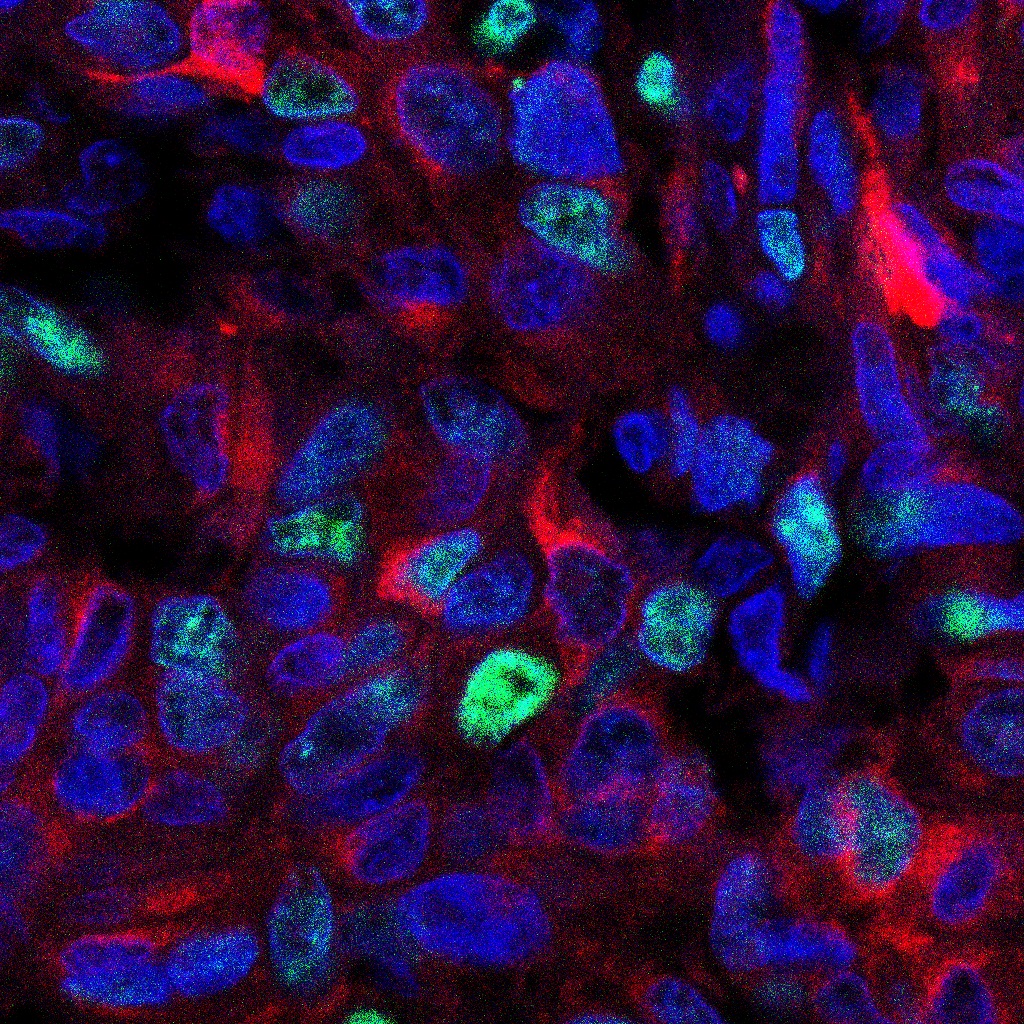

Supplement: Supplementary file 2 [file DataSheet_2.zip › ROW DATA Figure 1-2/Figure 2/Figure 2F/High/Merge.jpg]

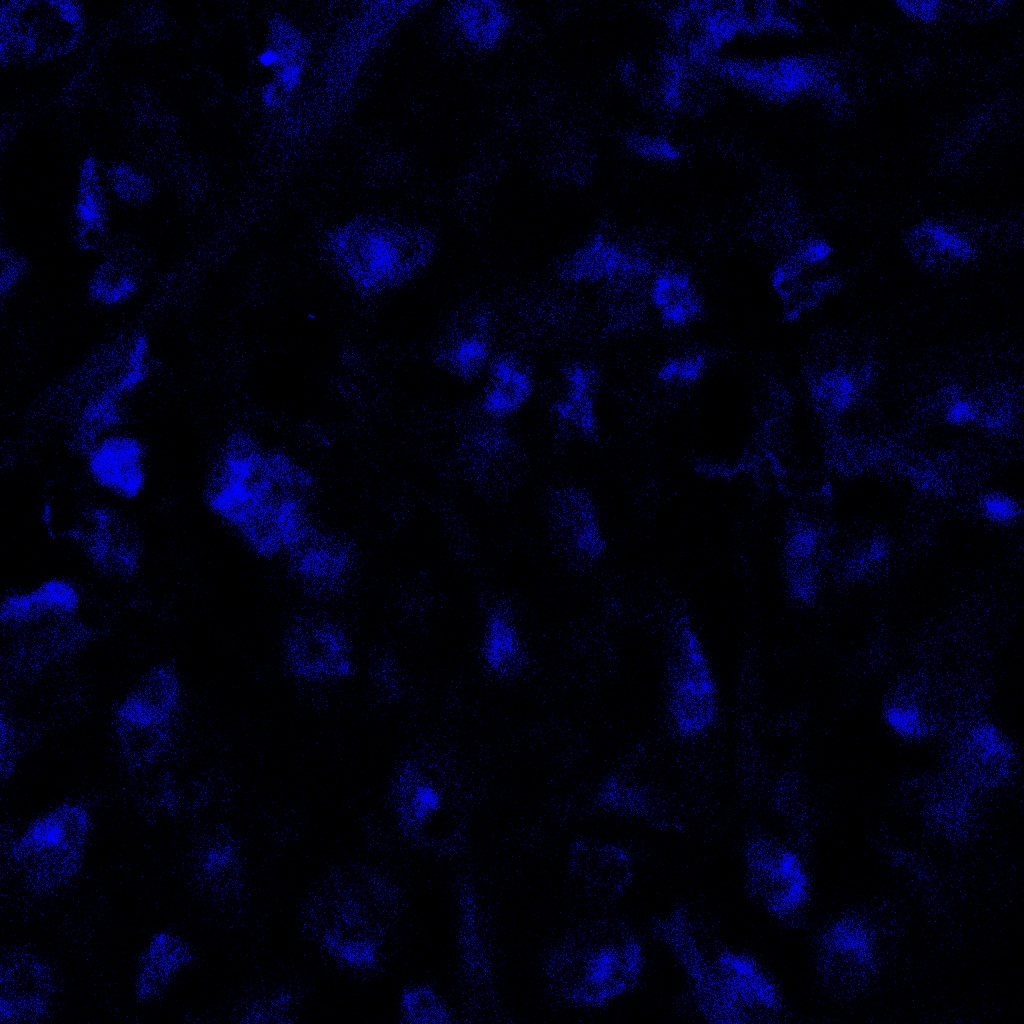

Supplement: Supplementary file 2 [file DataSheet_2.zip › ROW DATA Figure 1-2/Figure 2/Figure 2F/Low/DAPI.jpg]

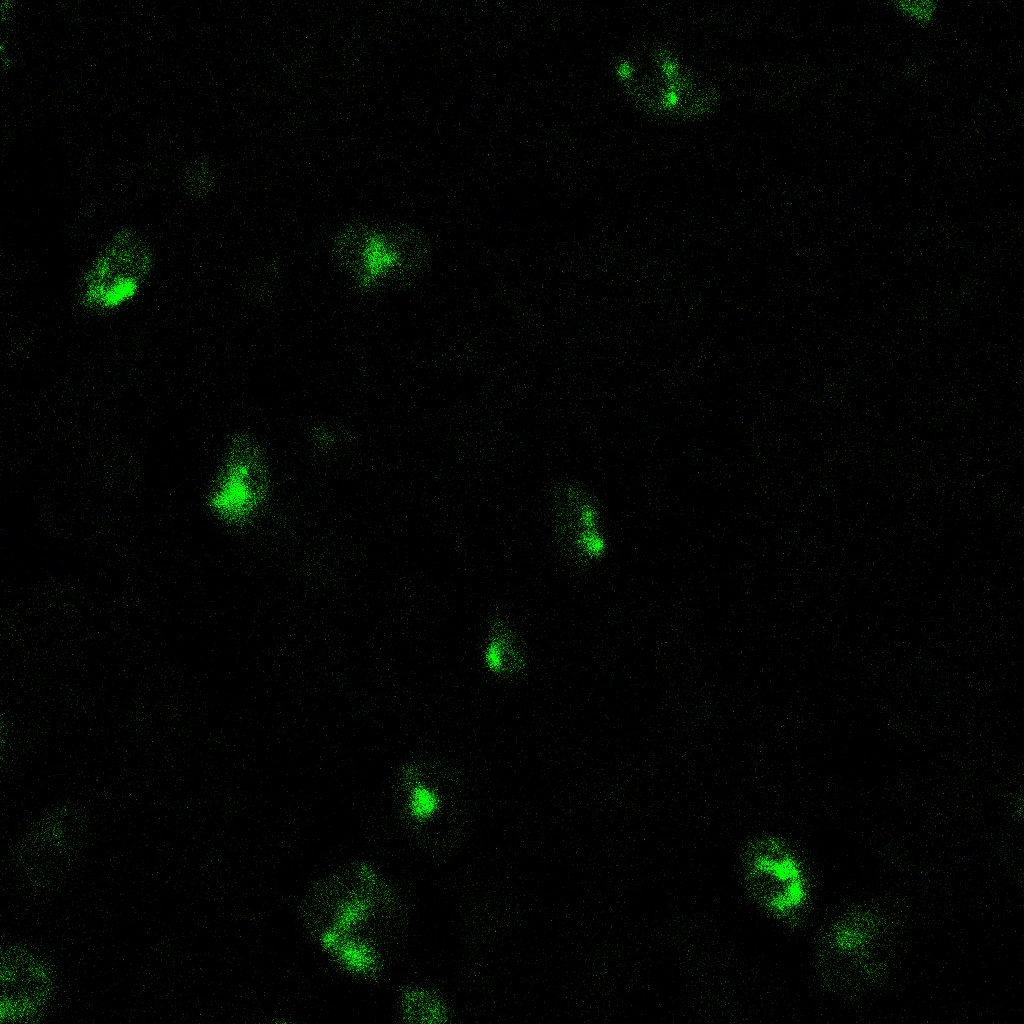

Supplement: Supplementary file 2 [file DataSheet_2.zip › ROW DATA Figure 1-2/Figure 2/Figure 2F/Low/Ki67.jpg]

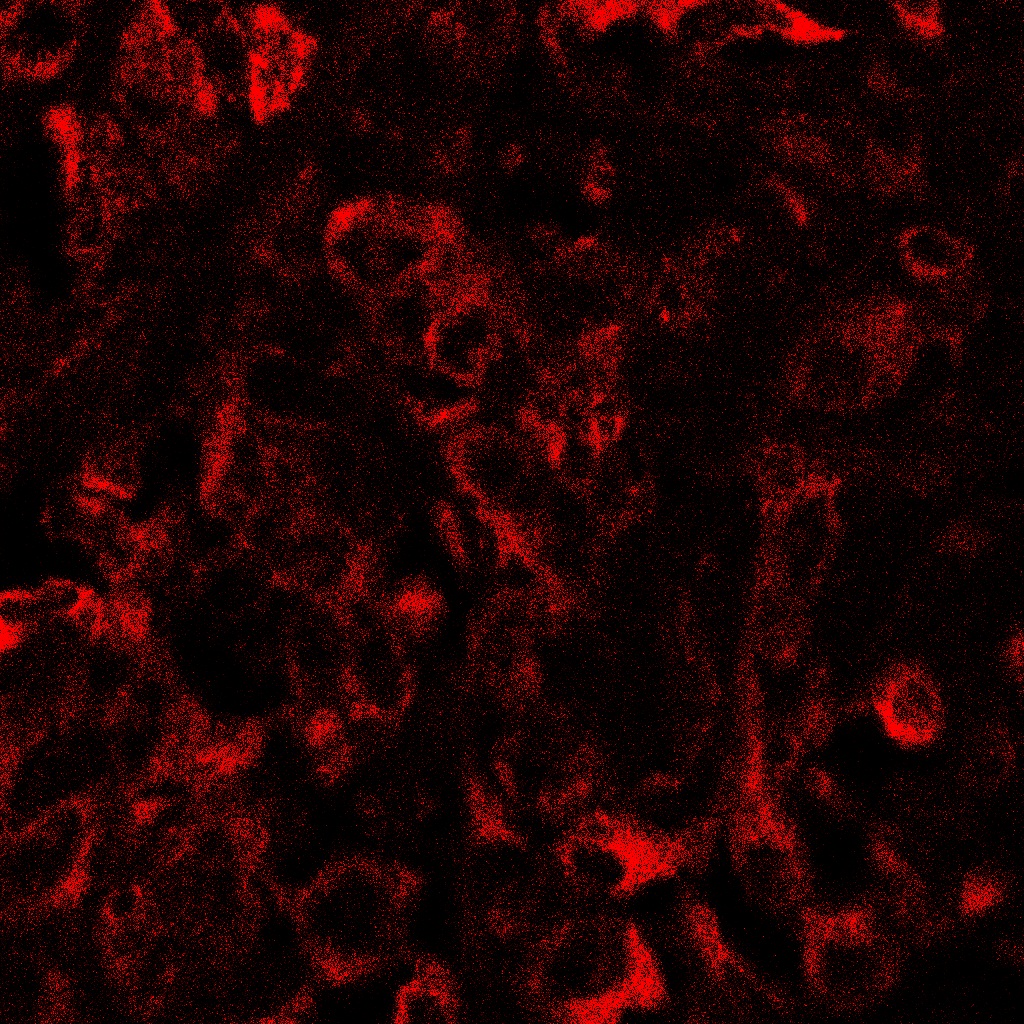

Supplement: Supplementary file 2 [file DataSheet_2.zip › ROW DATA Figure 1-2/Figure 2/Figure 2F/Low/Melan A.jpg]

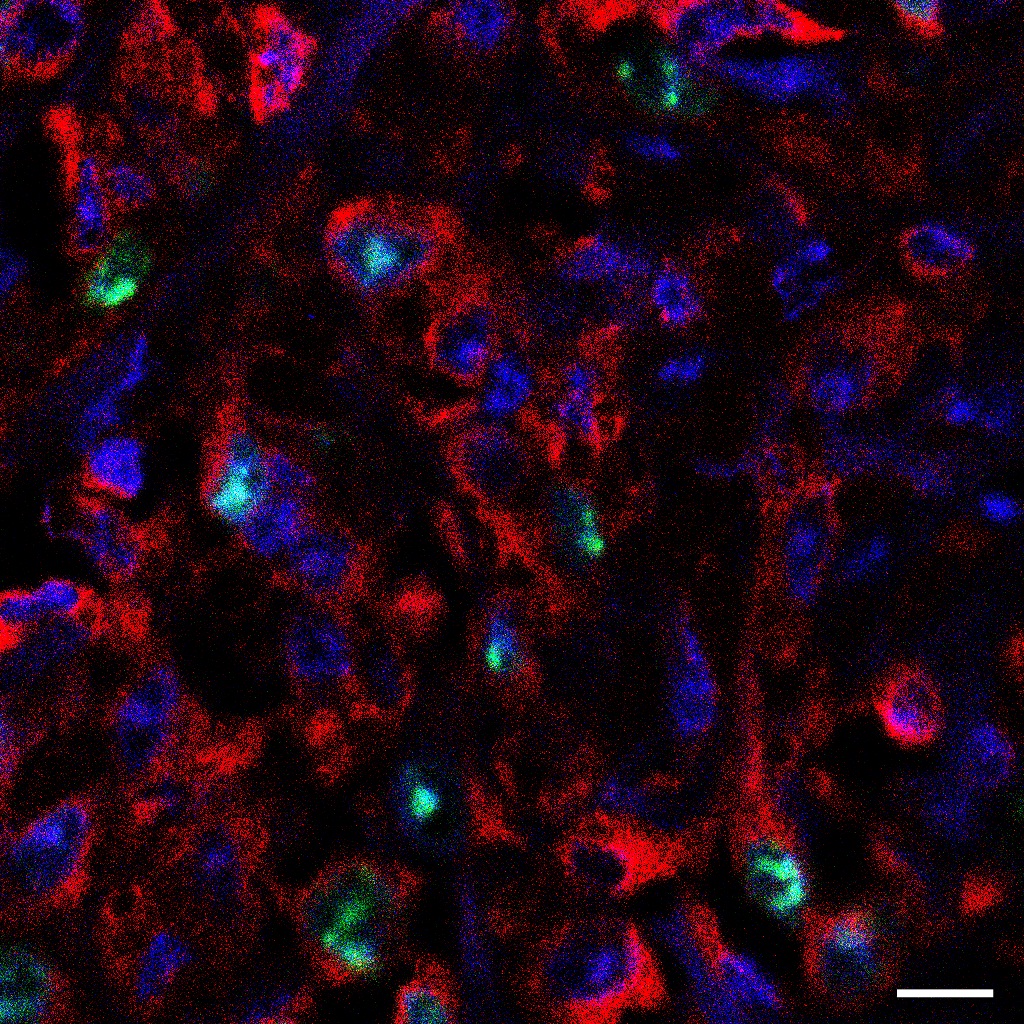

Supplement: Supplementary file 2 [file DataSheet_2.zip › ROW DATA Figure 1-2/Figure 2/Figure 2F/Low/Merge.jpg]

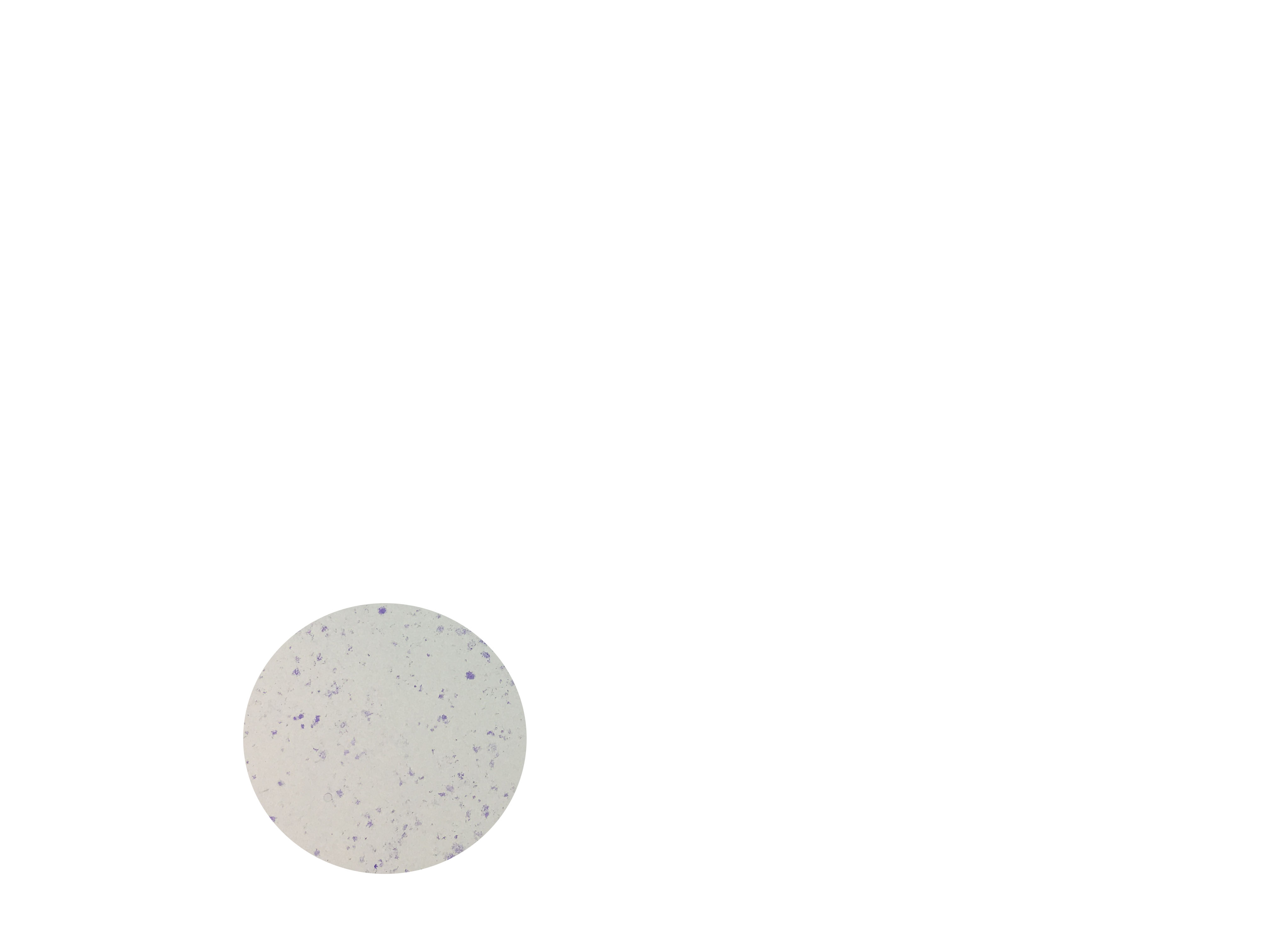

Supplement: Supplementary file 3 [file DataSheet_3.zip › ROW DATA Figure 3A and B/Figure 3B/A2058 siCD27-AS1-1.jpg]

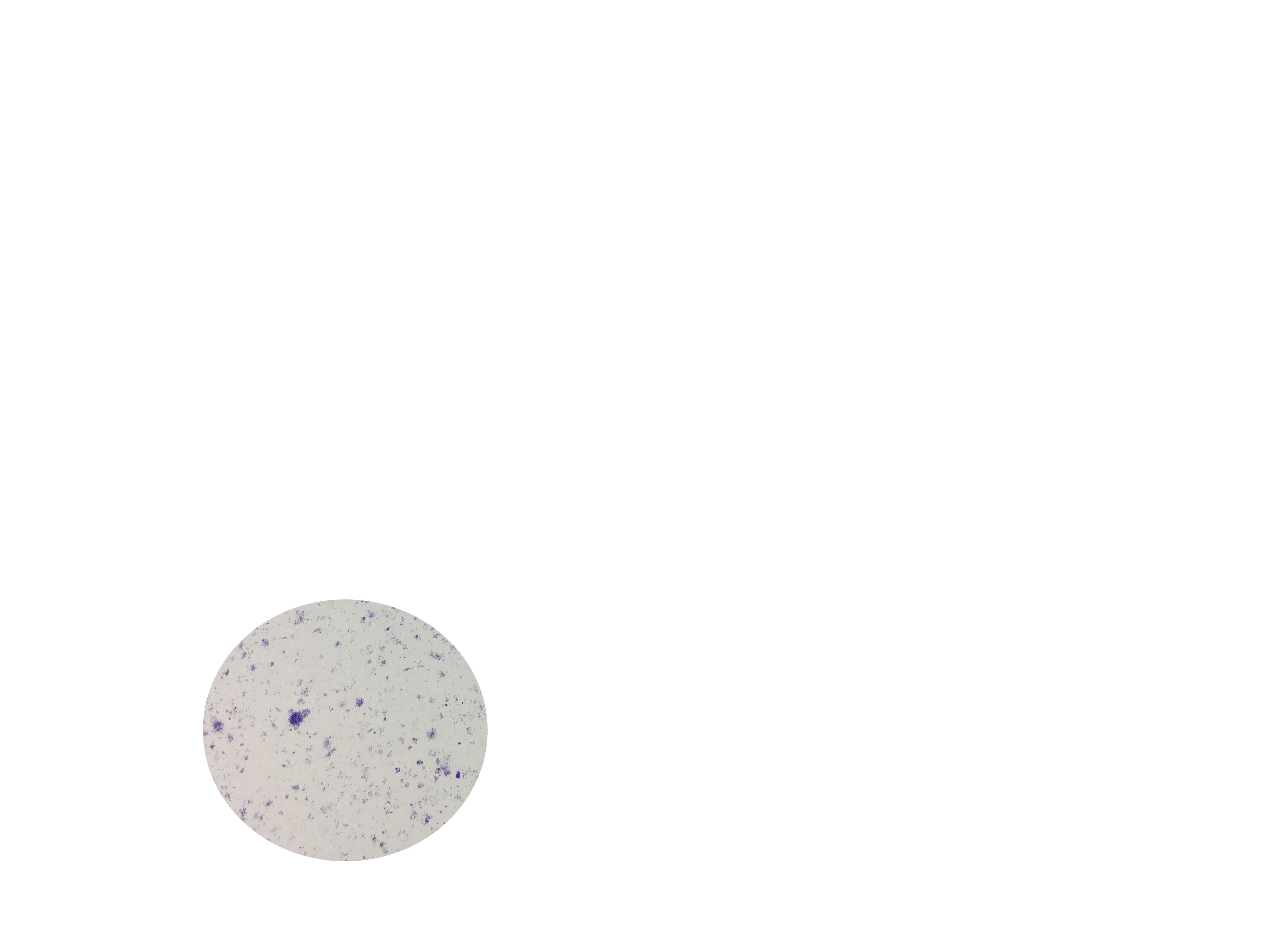

Supplement: Supplementary file 3 [file DataSheet_3.zip › ROW DATA Figure 3A and B/Figure 3B/A2058 siCD27-AS1-2.jpg]

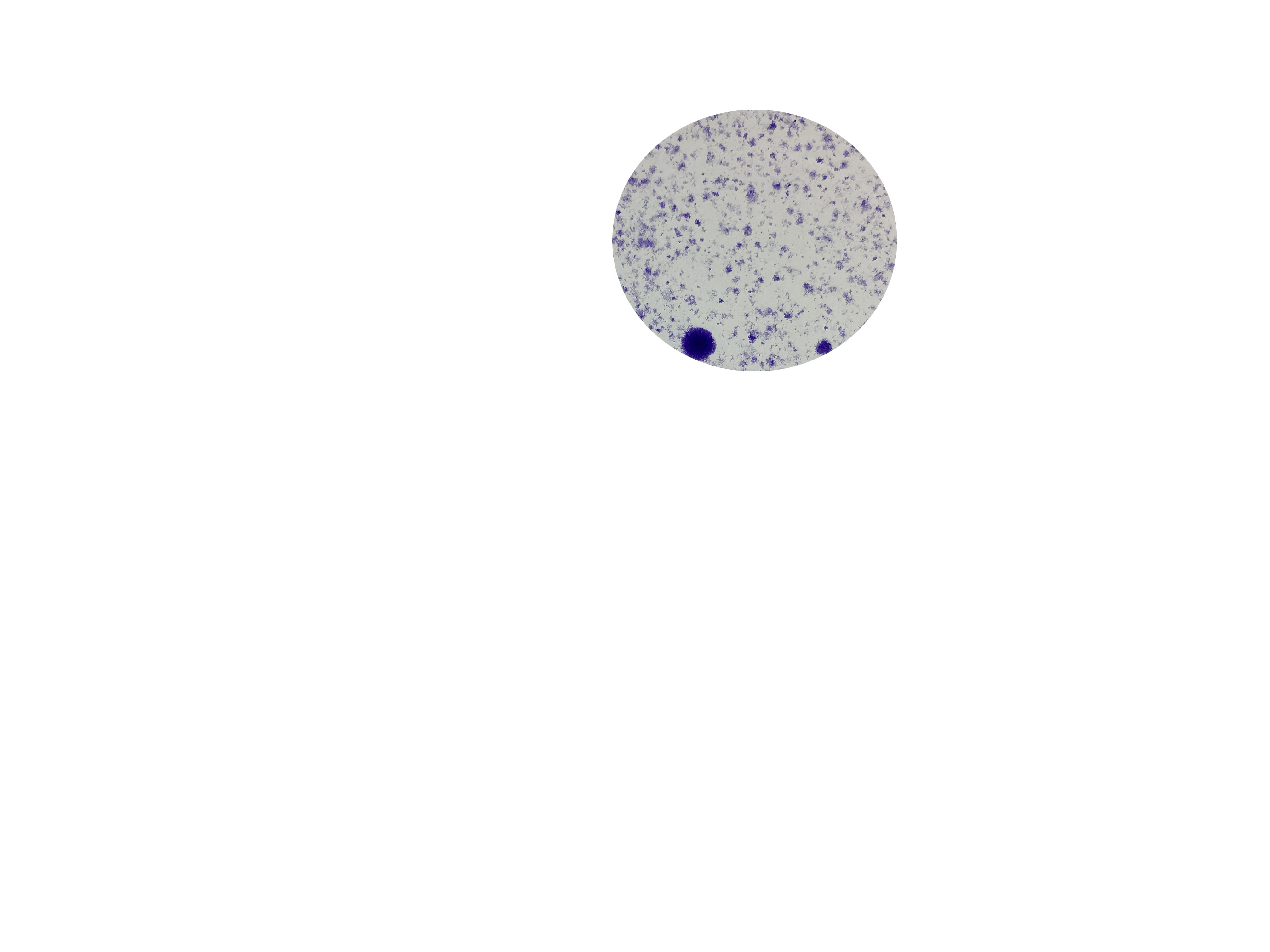

Supplement: Supplementary file 3 [file DataSheet_3.zip › ROW DATA Figure 3A and B/Figure 3B/A2058 siNC.jpg]

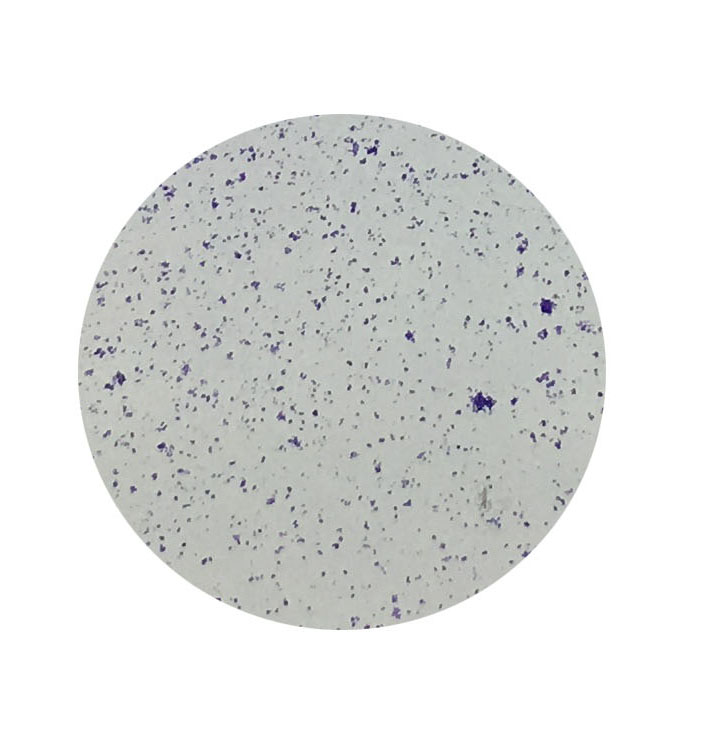

Supplement: Supplementary file 3 [file DataSheet_3.zip › ROW DATA Figure 3A and B/Figure 3B/A375 siCD27-AS1-208-1.jpg]

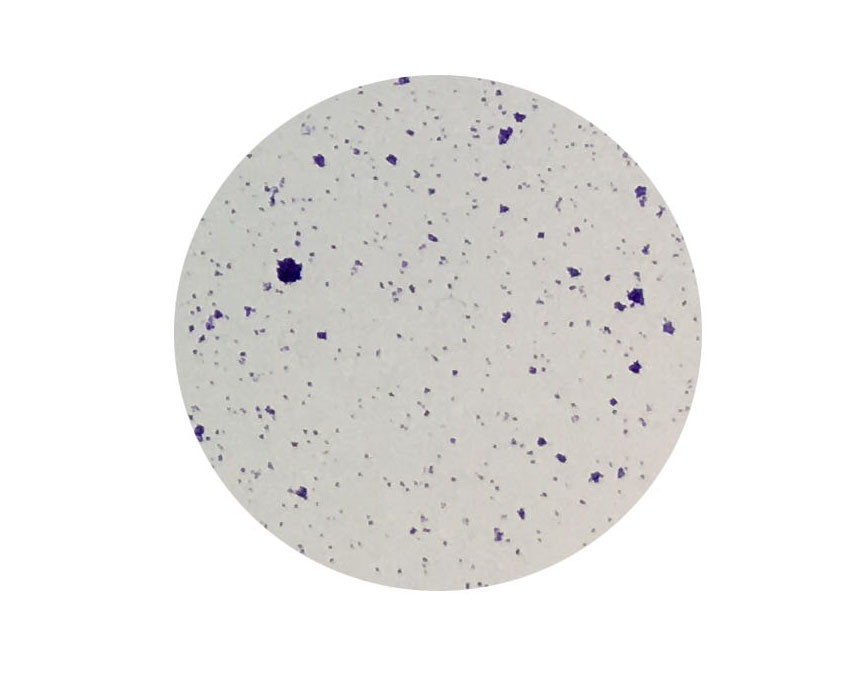

Supplement: Supplementary file 3 [file DataSheet_3.zip › ROW DATA Figure 3A and B/Figure 3B/A375 siCD27-AS1-208-2.jpg]

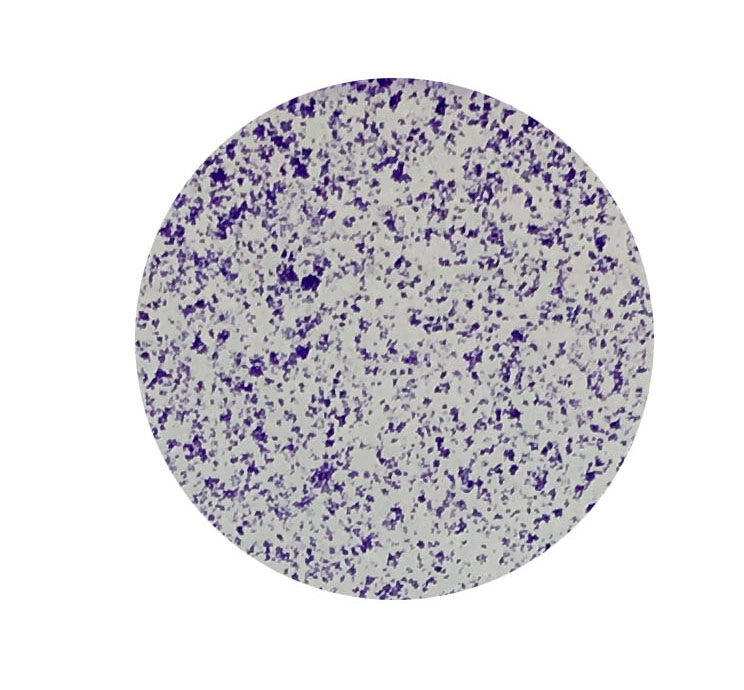

Supplement: Supplementary file 3 [file DataSheet_3.zip › ROW DATA Figure 3A and B/Figure 3B/A375 siNC.jpg]

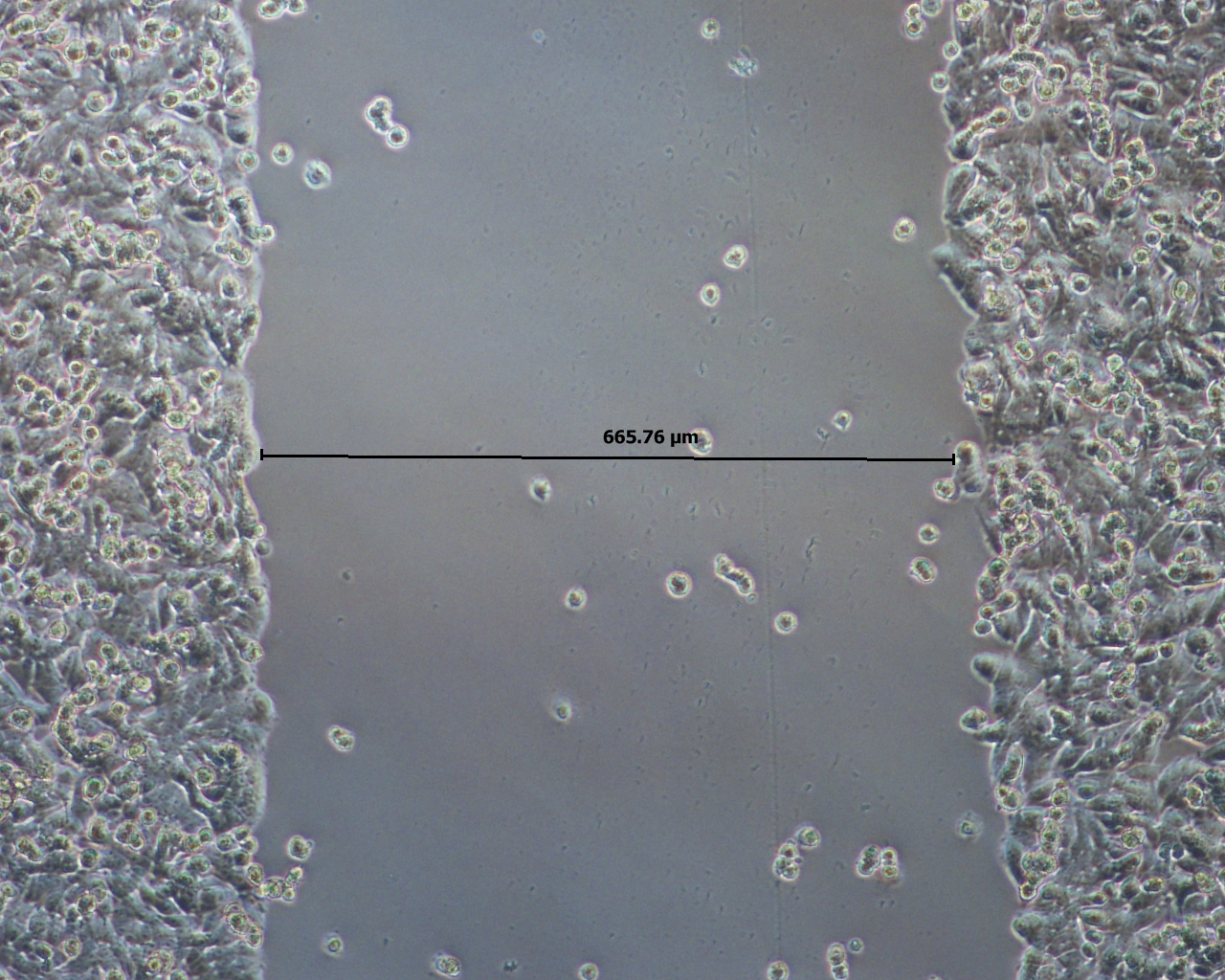

Supplement: Supplementary file 4 [file DataSheet_4.zip › ROW DATA Figure 3C A375/A375 siCD27-AS1-208-1 0h.tif]

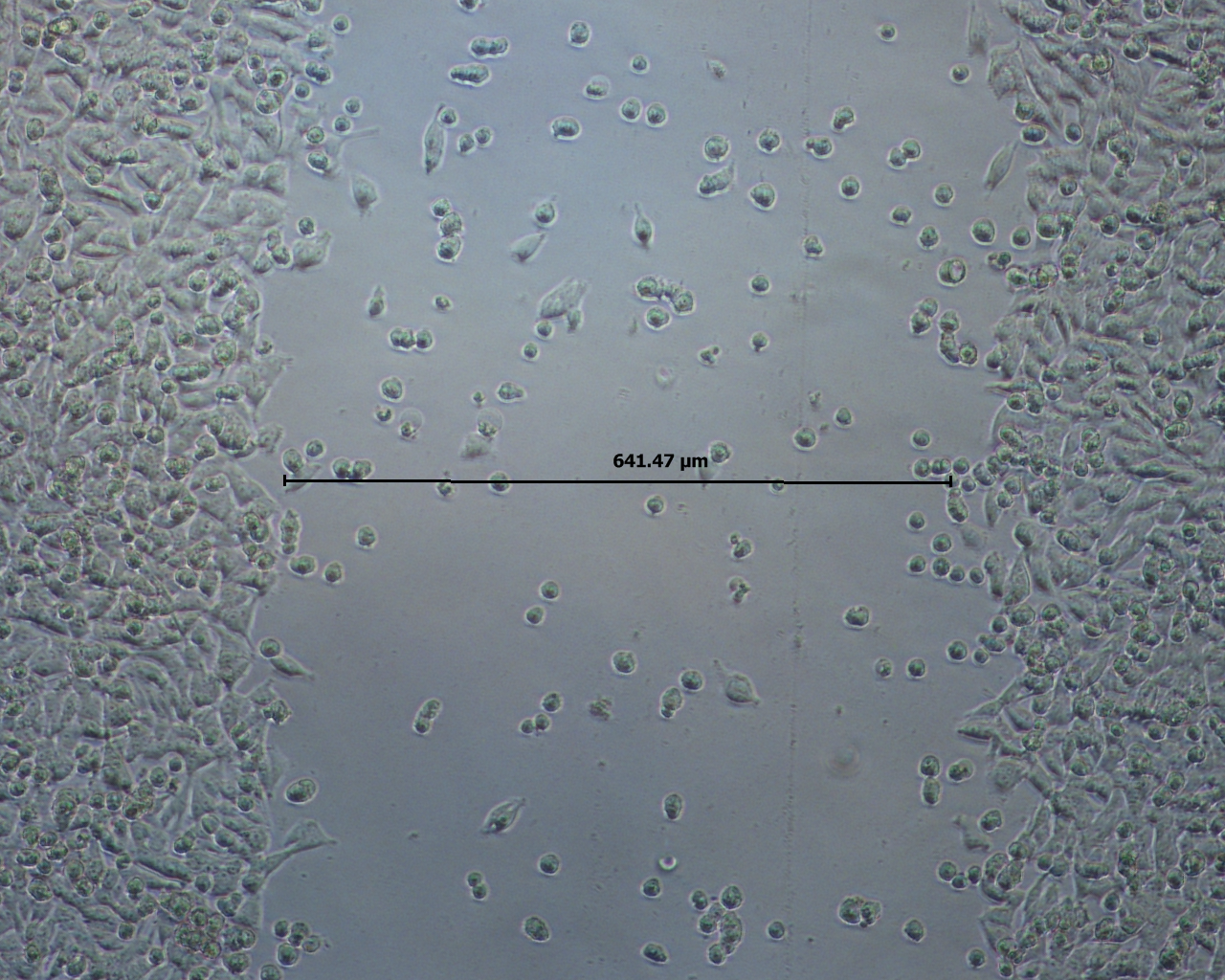

Supplement: Supplementary file 4 [file DataSheet_4.zip › ROW DATA Figure 3C A375/A375 siCD27-AS1-208-1 24h.tif]

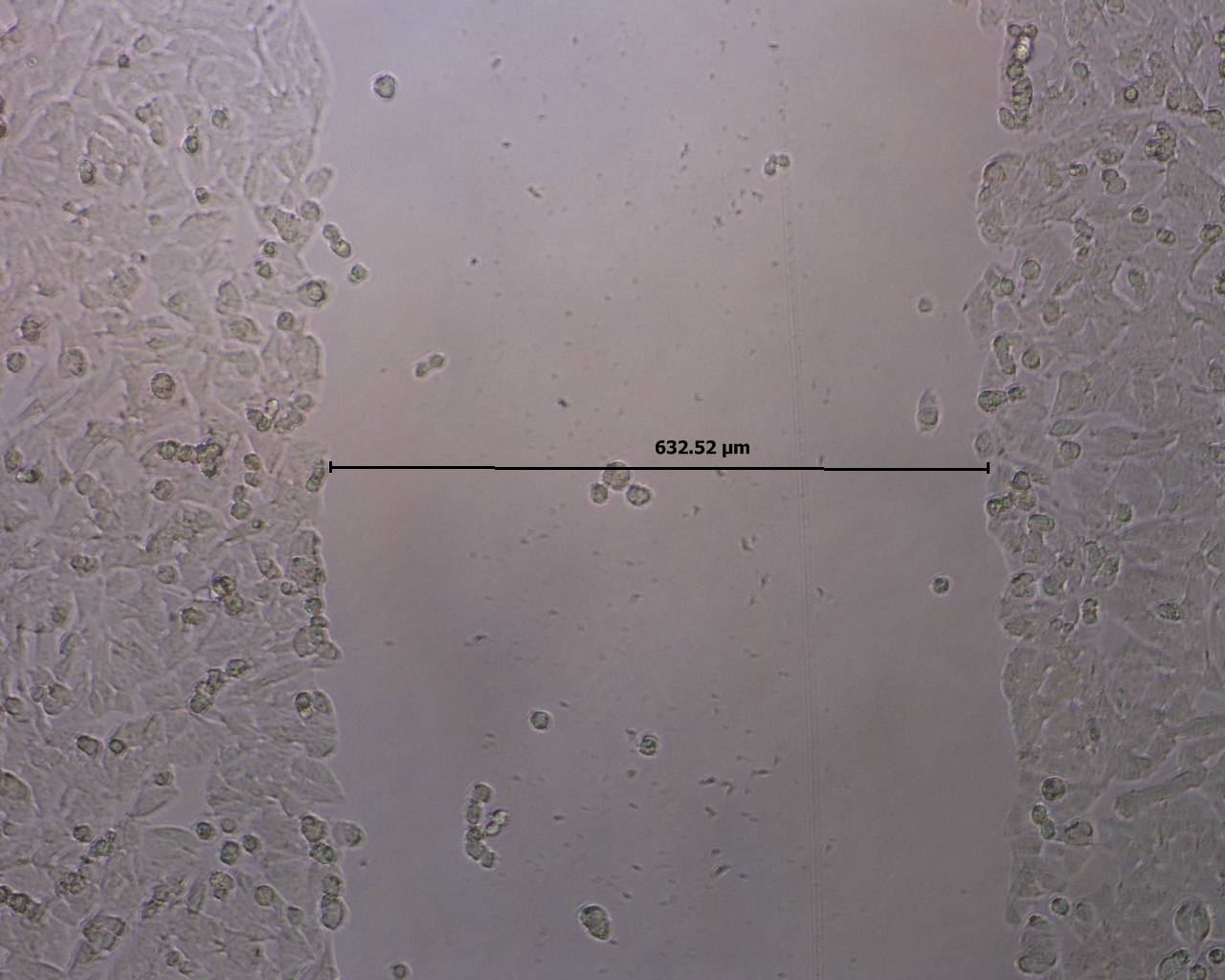

Supplement: Supplementary file 4 [file DataSheet_4.zip › ROW DATA Figure 3C A375/A375 siCD27-AS1-208-2 0h.tif]

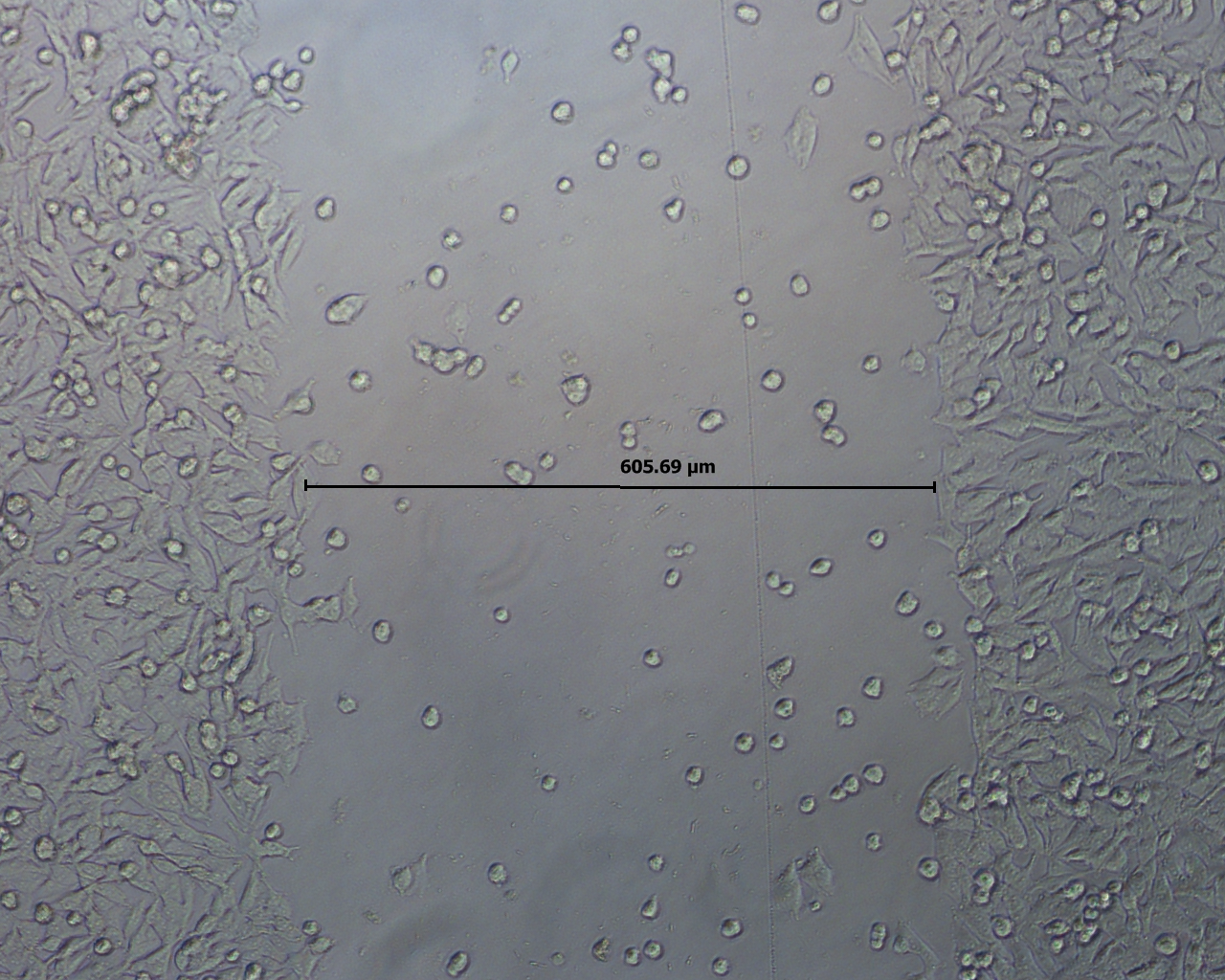

Supplement: Supplementary file 4 [file DataSheet_4.zip › ROW DATA Figure 3C A375/A375 siCD27-AS1-208-2 24h.tif]

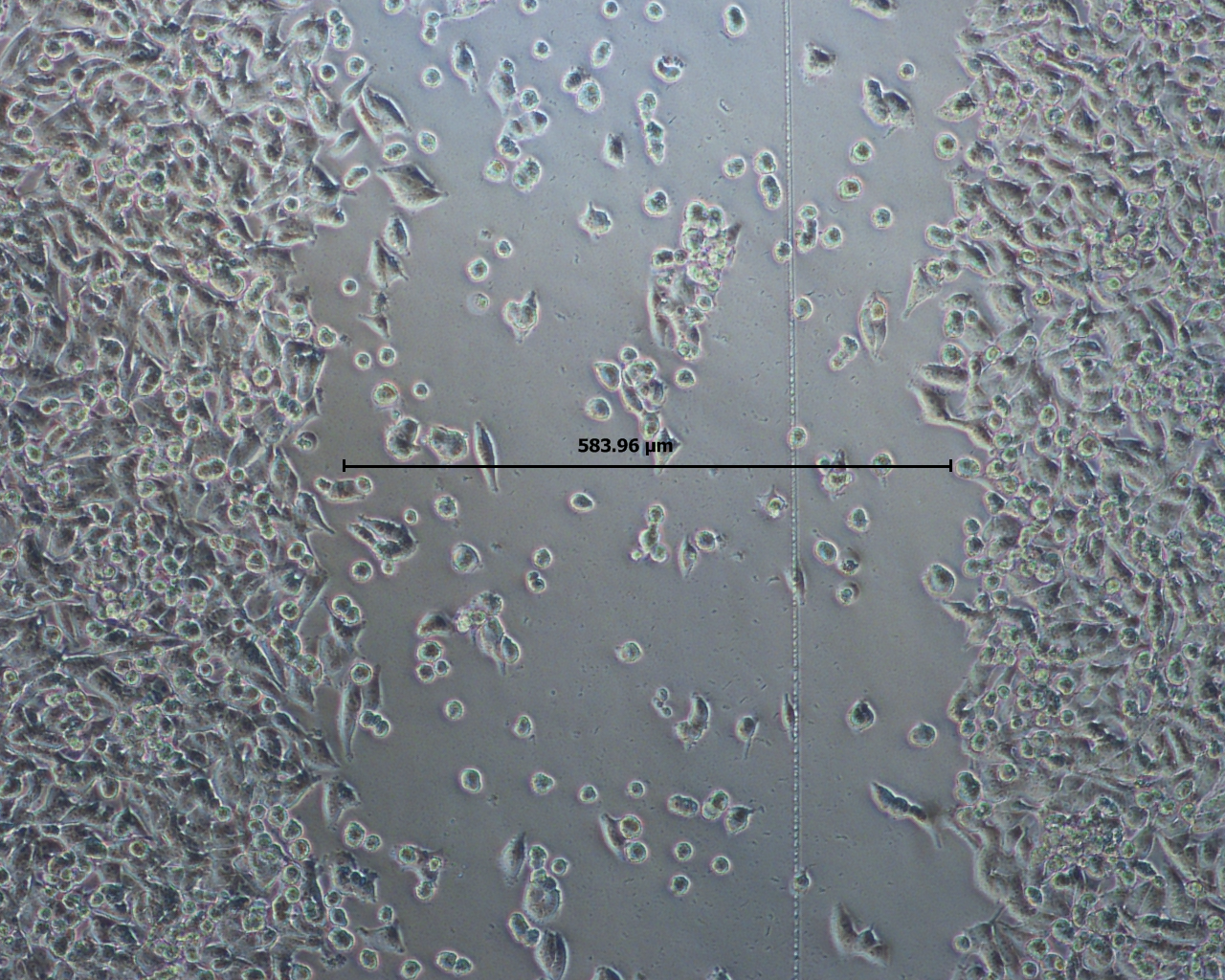

Supplement: Supplementary file 4 [file DataSheet_4.zip › ROW DATA Figure 3C A375/A375 siNC 24h.tif]

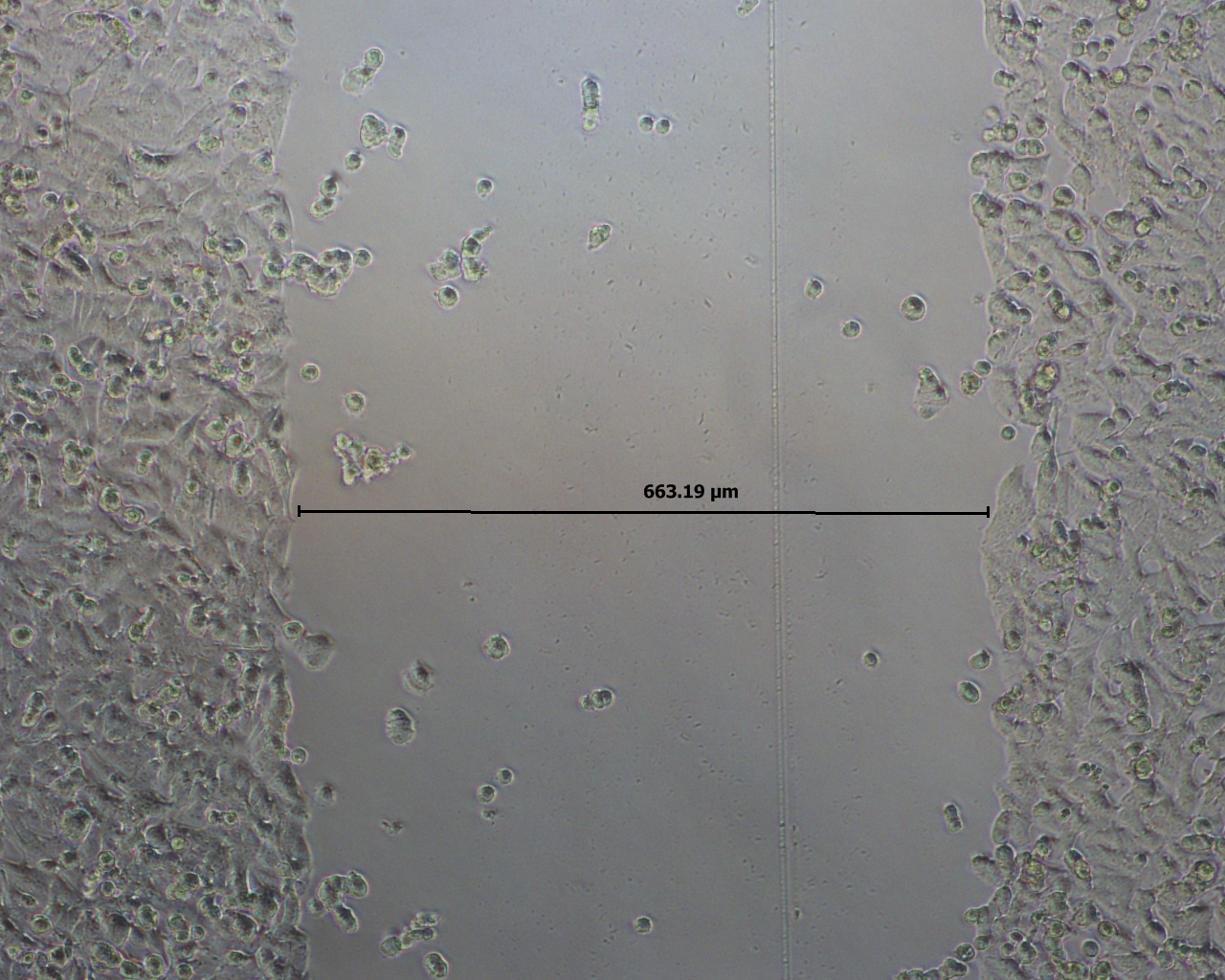

Supplement: Supplementary file 4 [file DataSheet_4.zip › ROW DATA Figure 3C A375/A375 siNC-0h.tif]

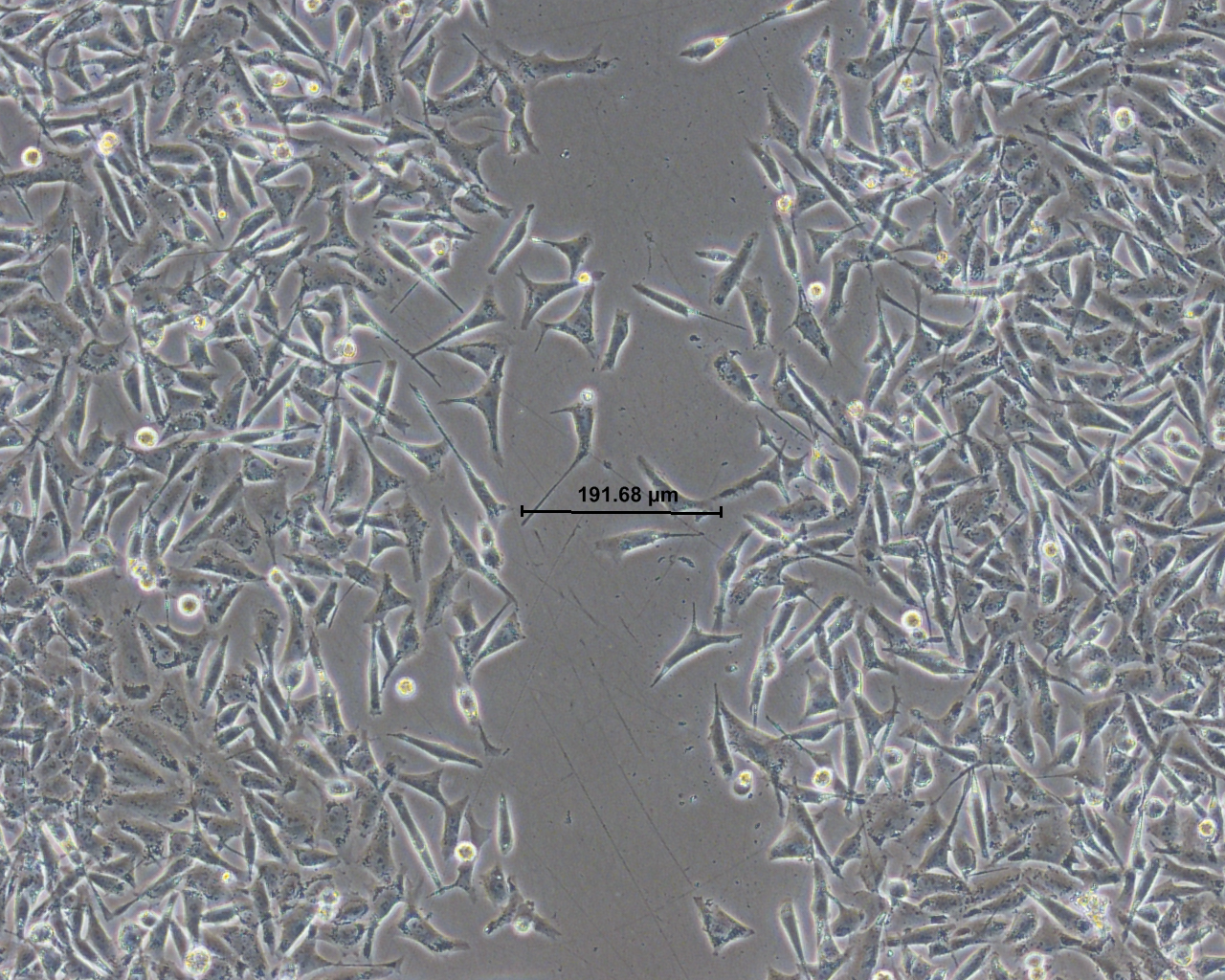

Supplement: Supplementary file 5 [file DataSheet_5.zip › ROW DATA Figure 3C A2058/A2058 siNC 24h.tif]

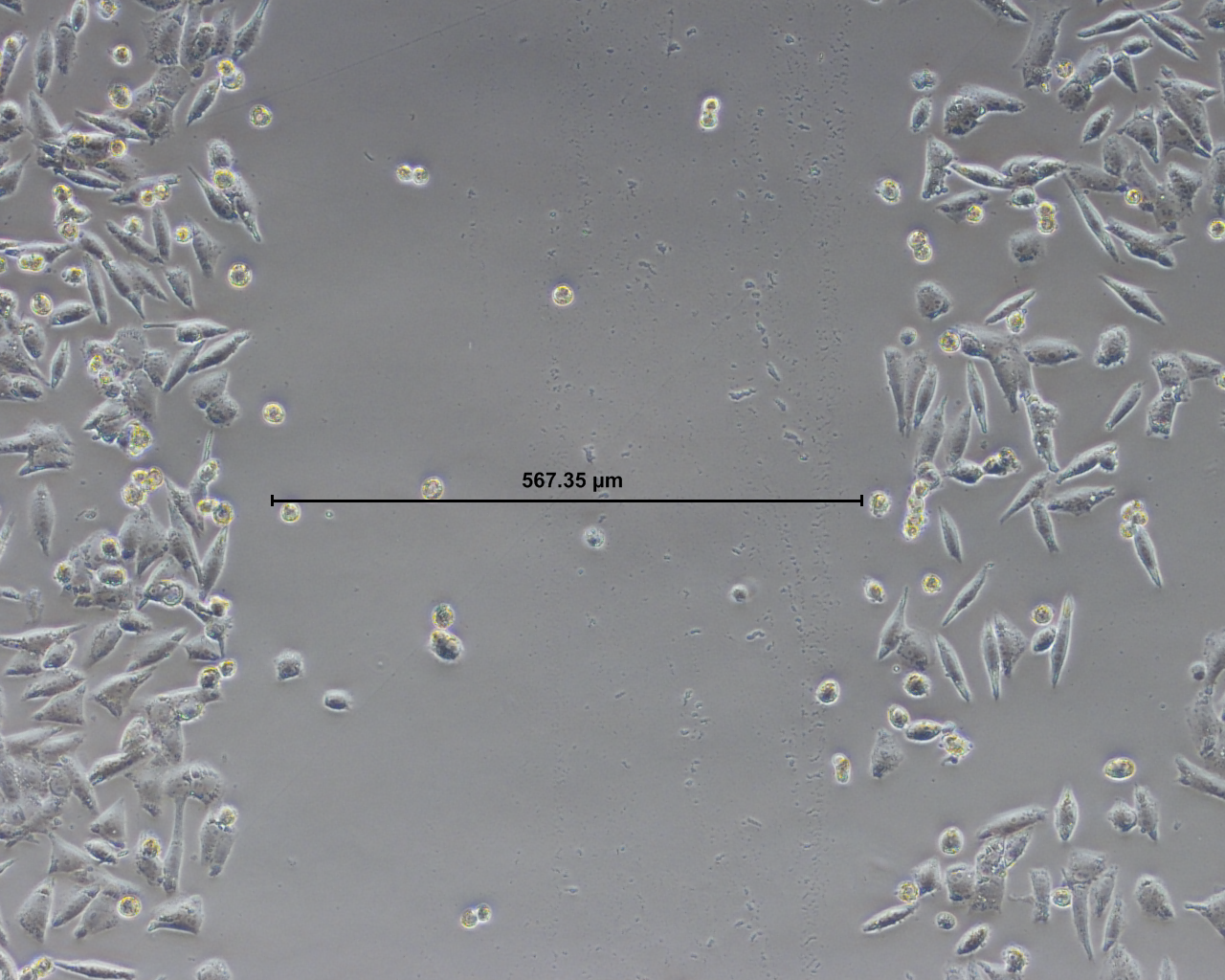

Supplement: Supplementary file 5 [file DataSheet_5.zip › ROW DATA Figure 3C A2058/A2058 siCD27-AS1-208-1 0h.tif]

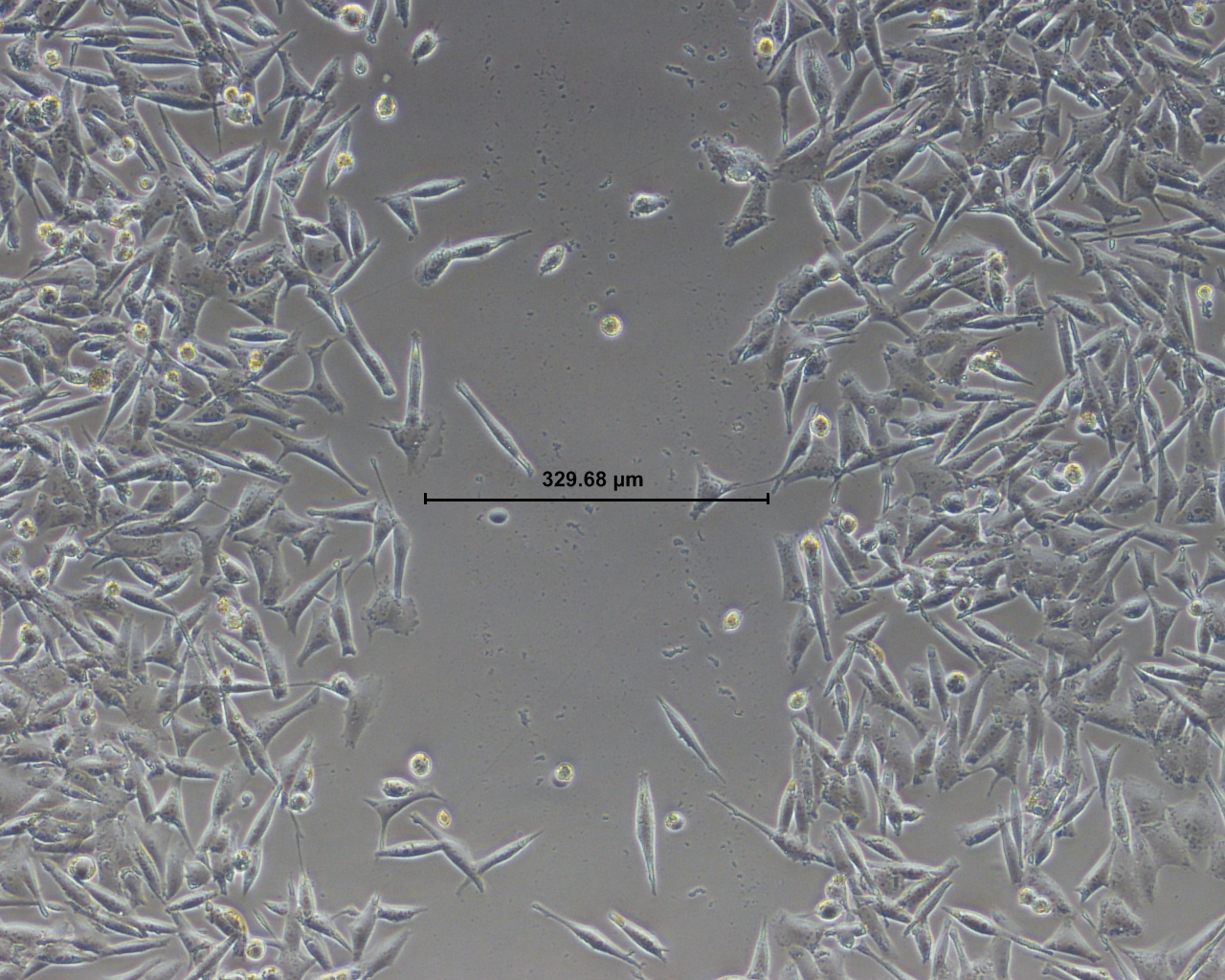

Supplement: Supplementary file 5 [file DataSheet_5.zip › ROW DATA Figure 3C A2058/A2058 siCD27-AS1-208-1 24h.tif]

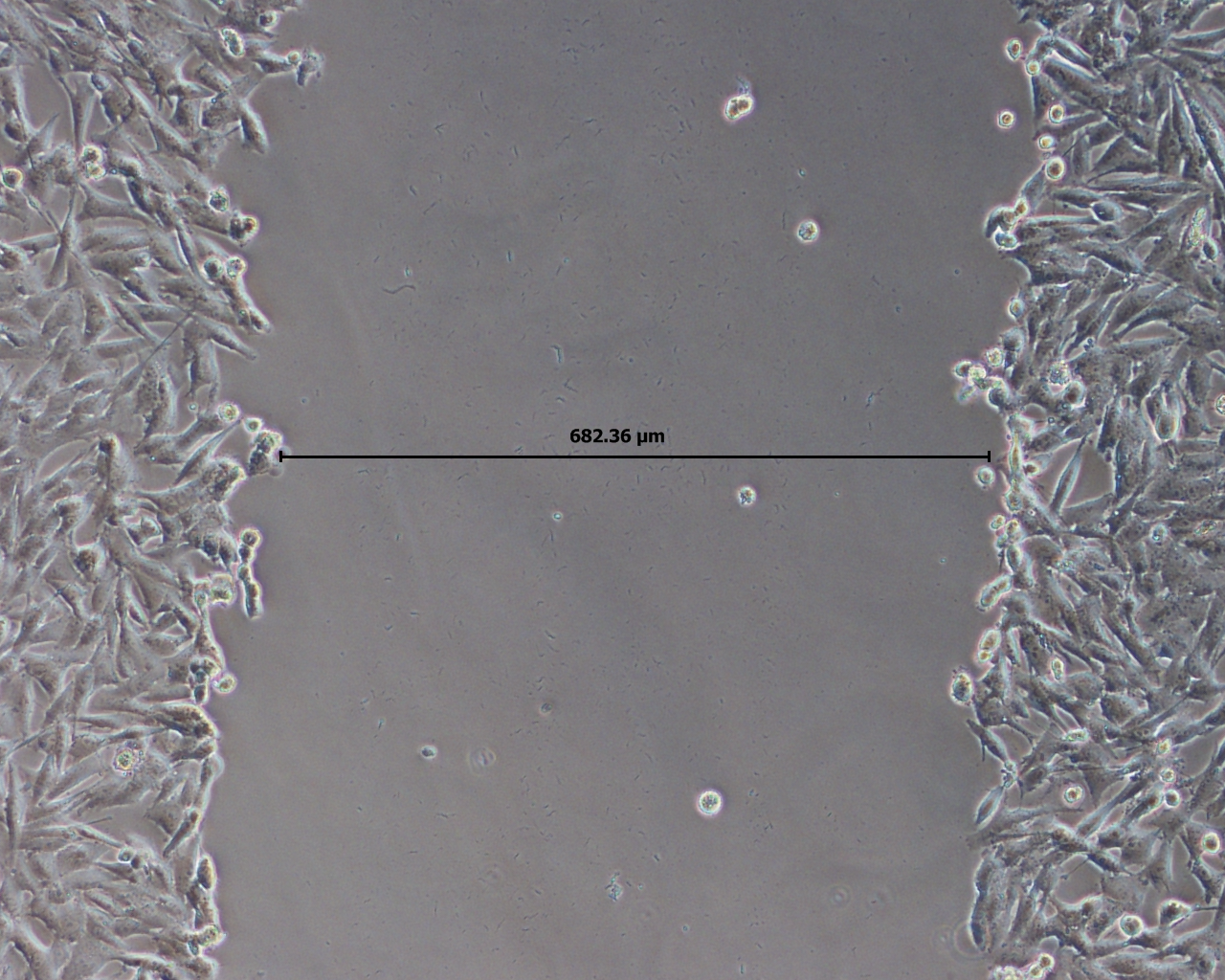

Supplement: Supplementary file 5 [file DataSheet_5.zip › ROW DATA Figure 3C A2058/A2058 siCD27-AS1-208-2 0h.tif]

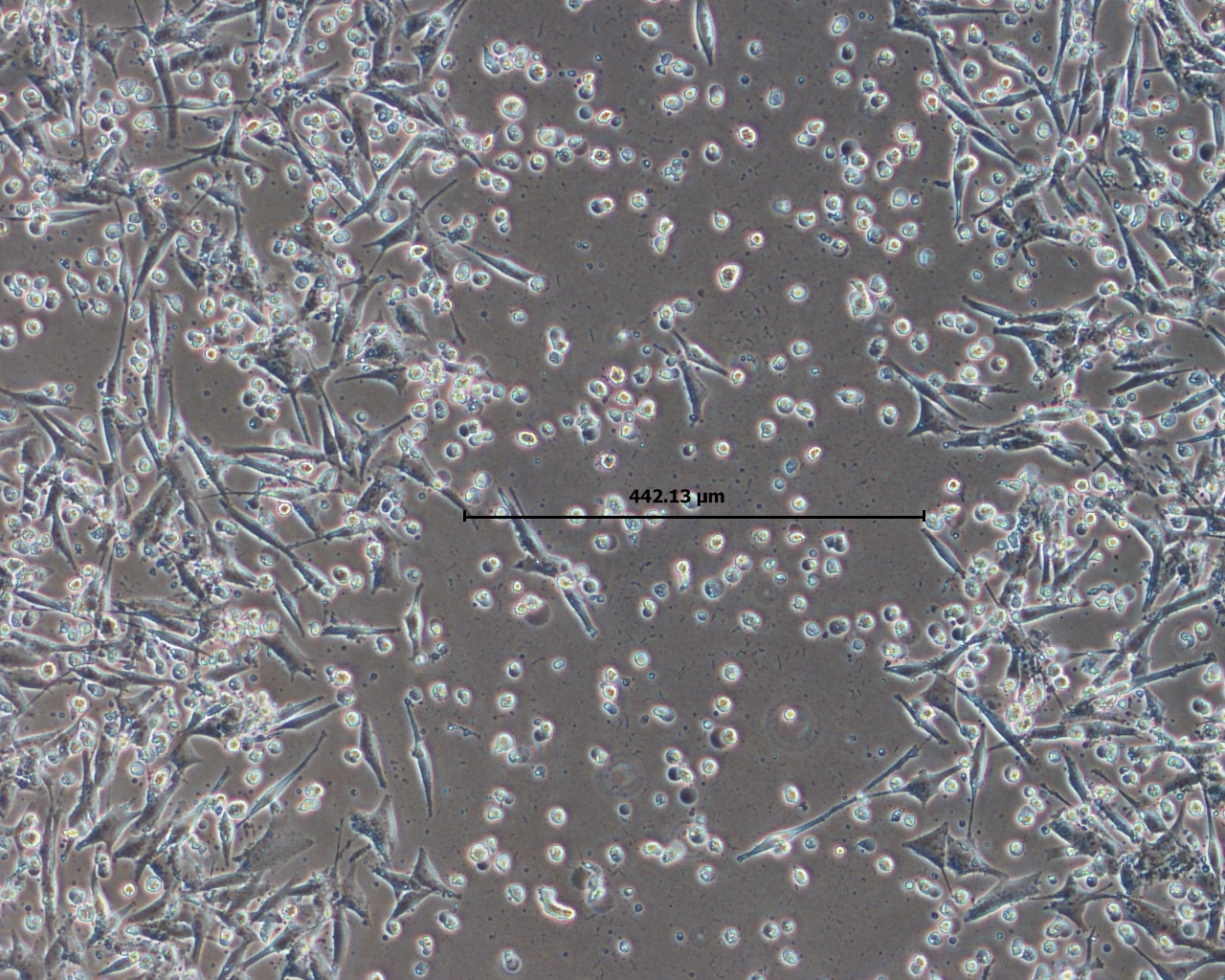

Supplement: Supplementary file 5 [file DataSheet_5.zip › ROW DATA Figure 3C A2058/A2058 siCD27-AS1-208-2 24h.tif]

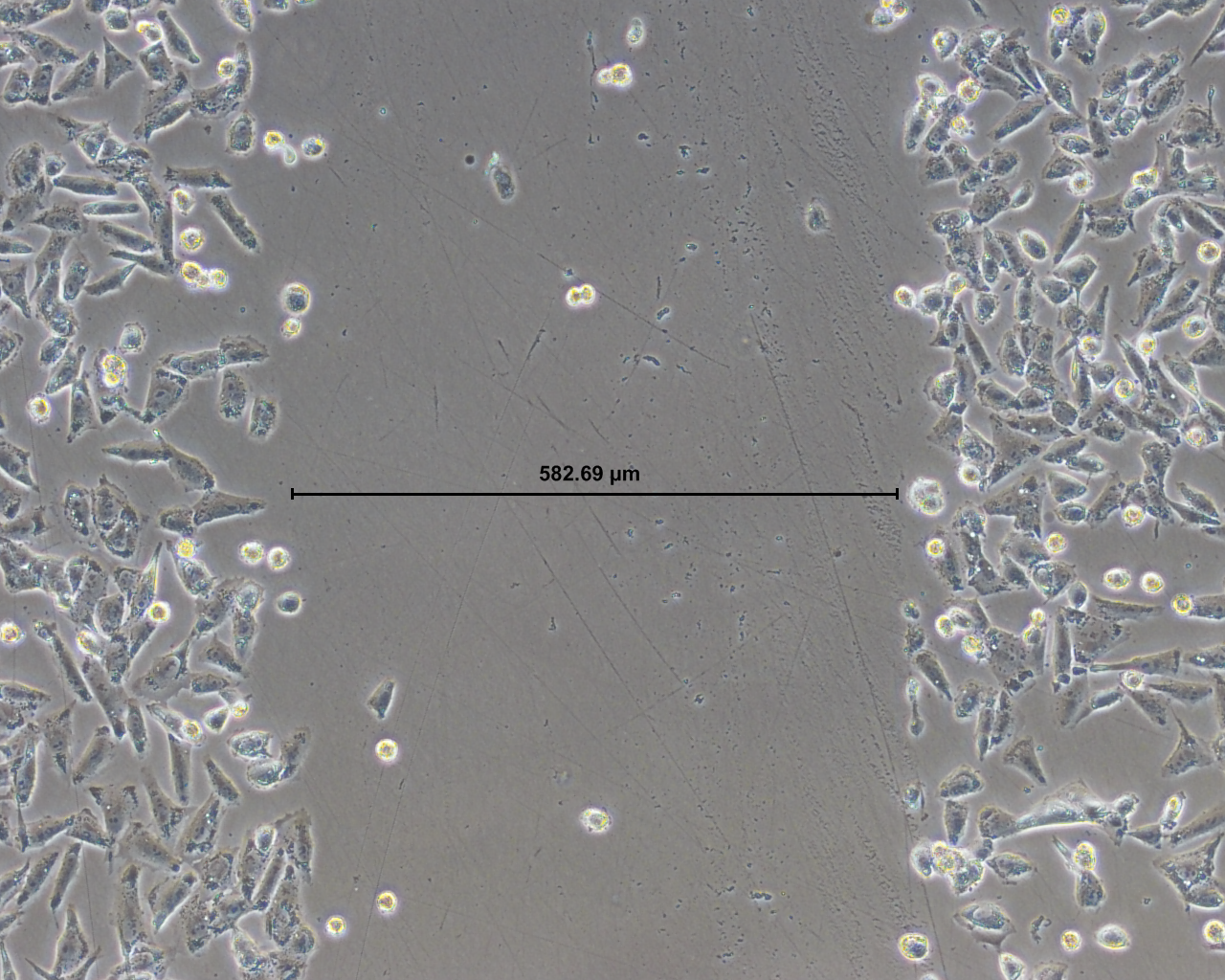

Supplement: Supplementary file 5 [file DataSheet_5.zip › ROW DATA Figure 3C A2058/A2058 siNC 0h.tif]

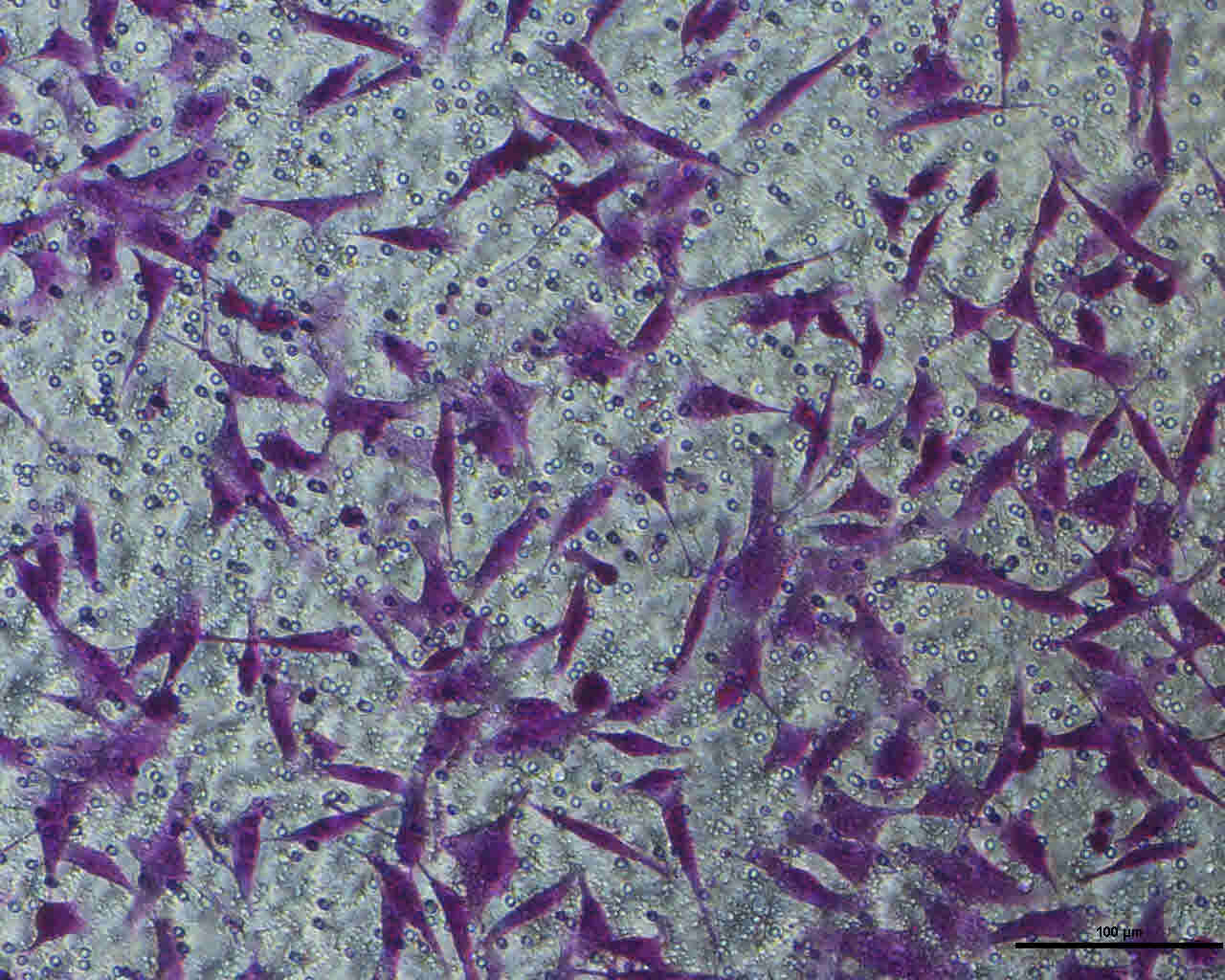

Supplement: Supplementary file 6 [file DataSheet_6.zip › Figure 3D/A2058 siCD27-AS1-208-1.jpg]

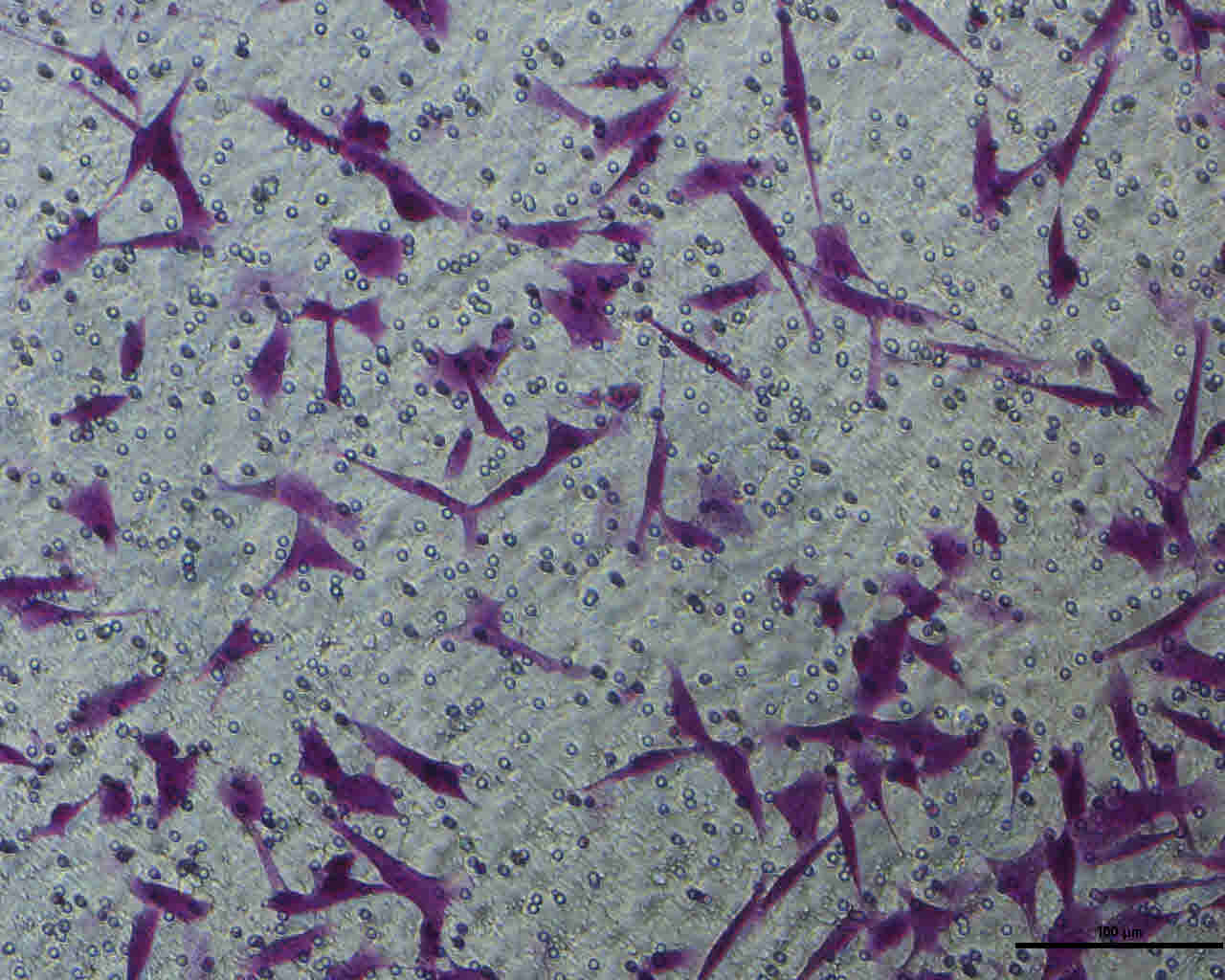

Supplement: Supplementary file 6 [file DataSheet_6.zip › Figure 3D/A2058 siCD27-AS1-208-2.jpg]

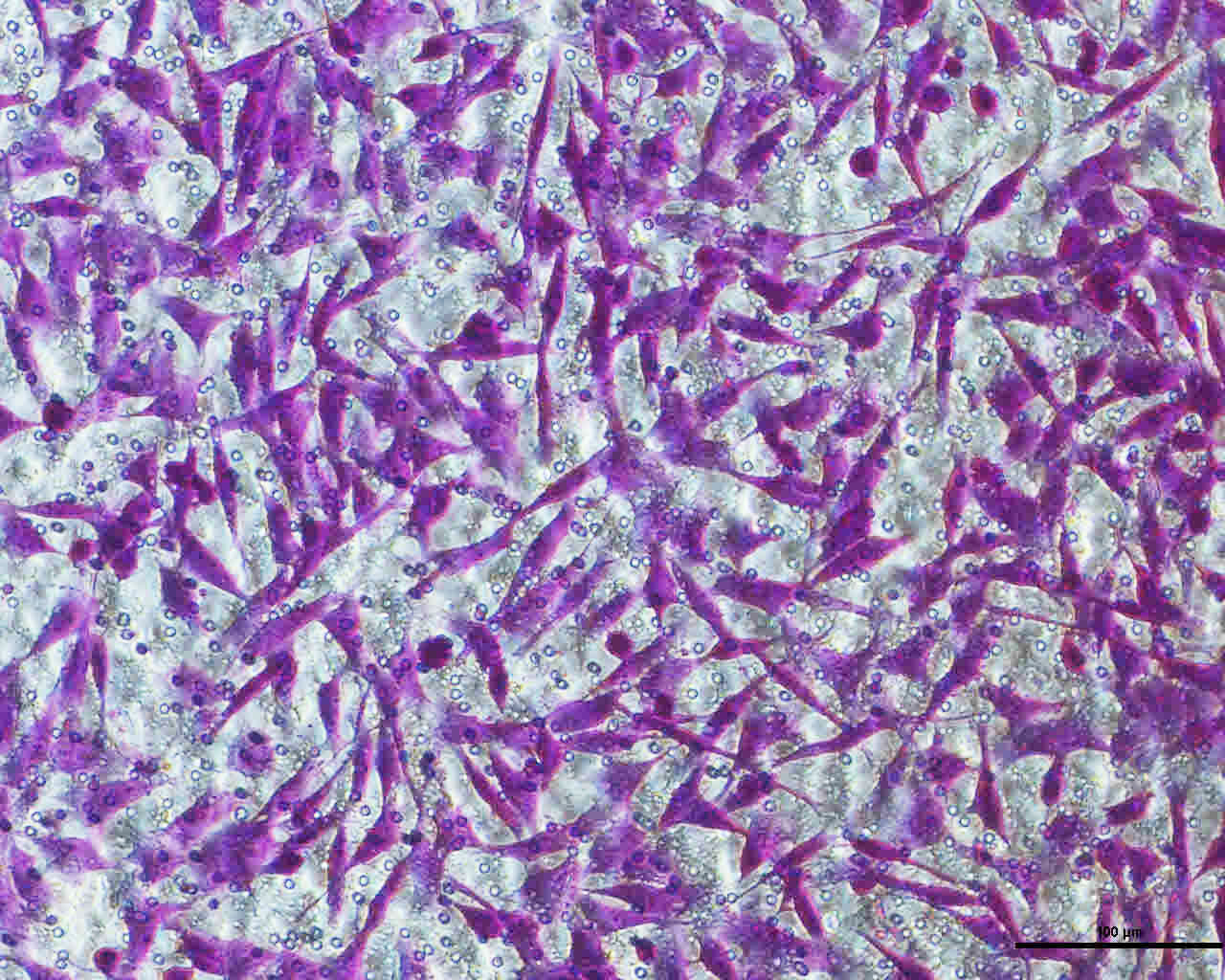

Supplement: Supplementary file 6 [file DataSheet_6.zip › Figure 3D/A2058 siNC.jpg]

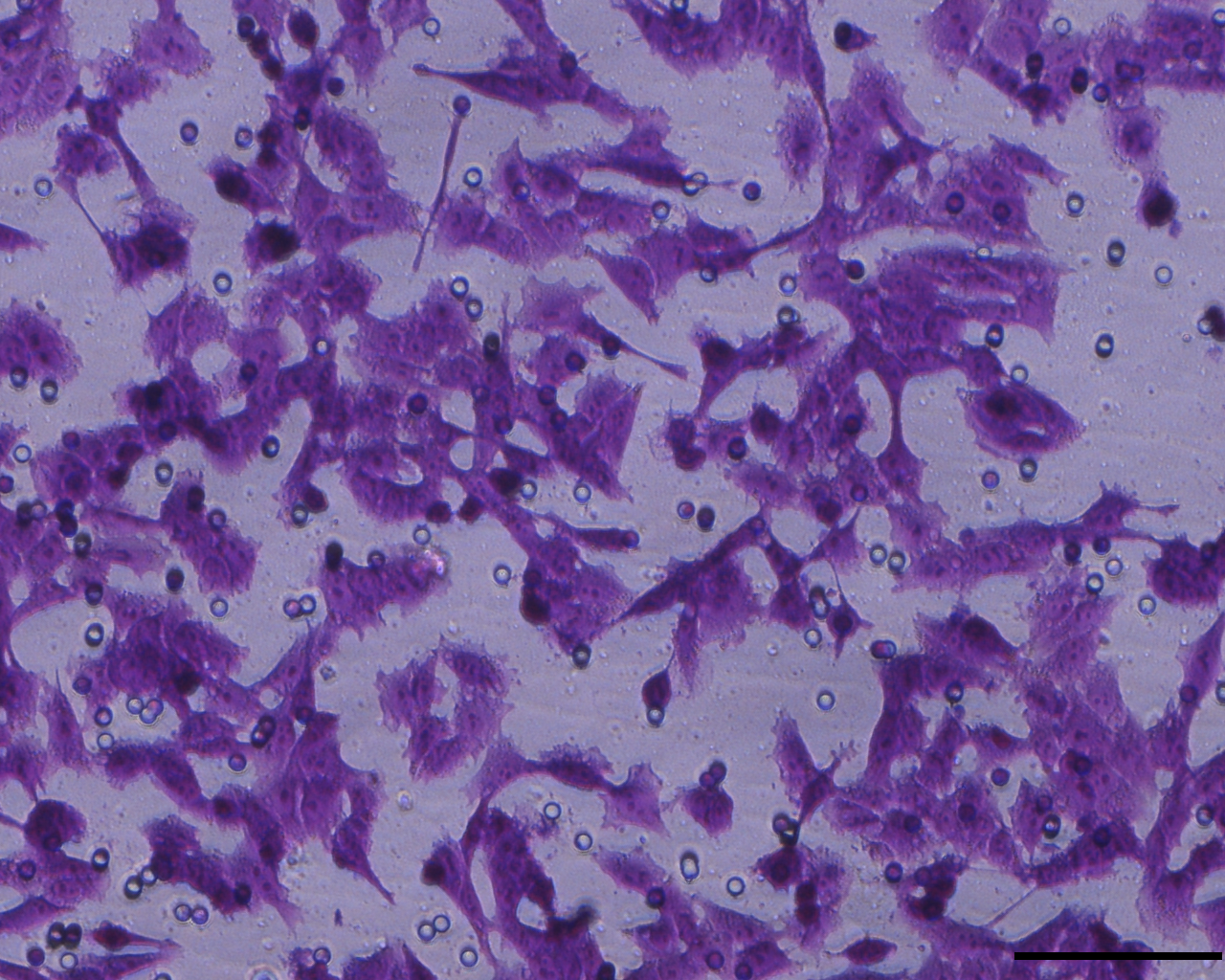

Supplement: Supplementary file 6 [file DataSheet_6.zip › Figure 3D/A375 siCD27-AS1-208-1.tif]

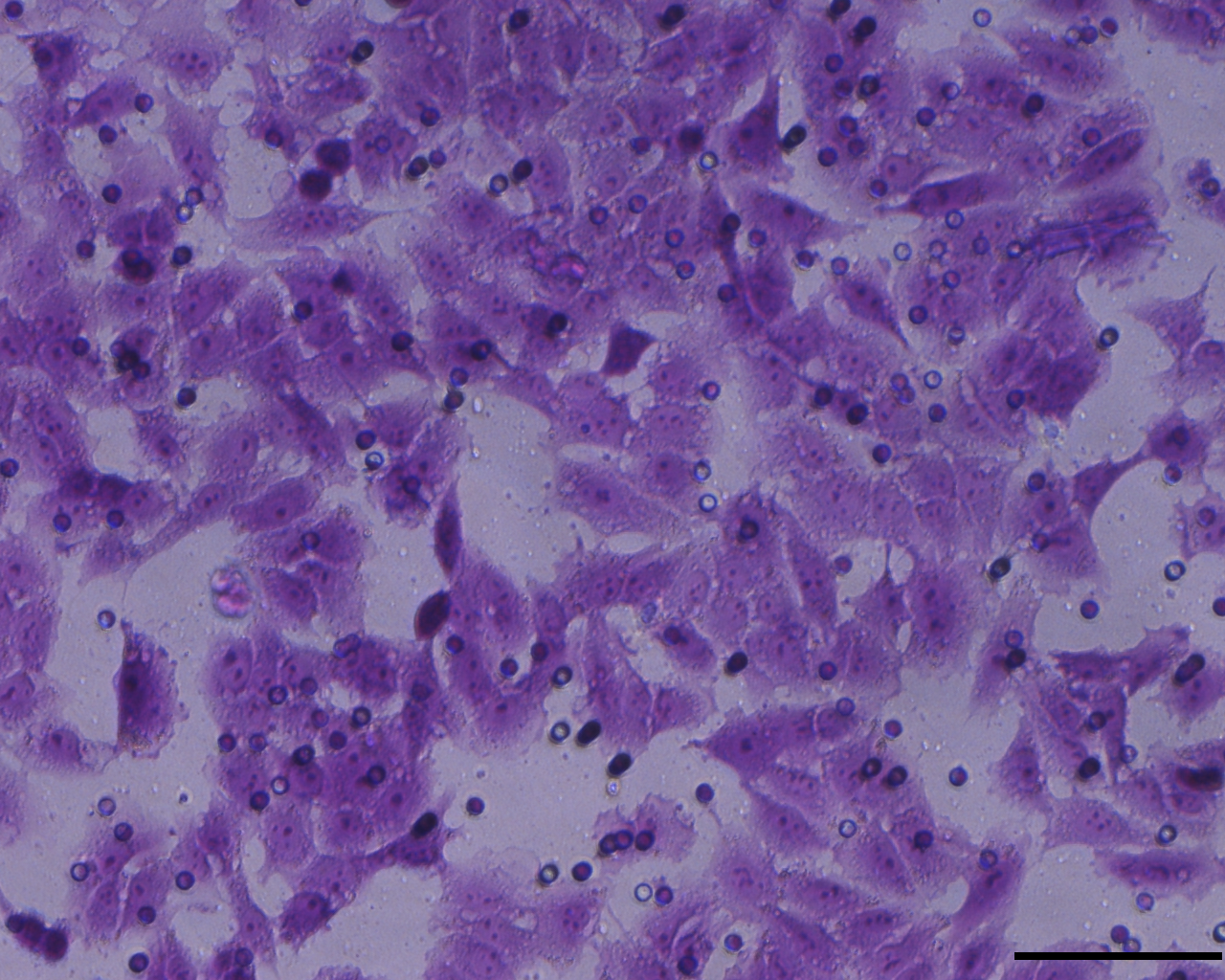

Supplement: Supplementary file 6 [file DataSheet_6.zip › Figure 3D/A375 siCD27-AS1-208-2.tif]

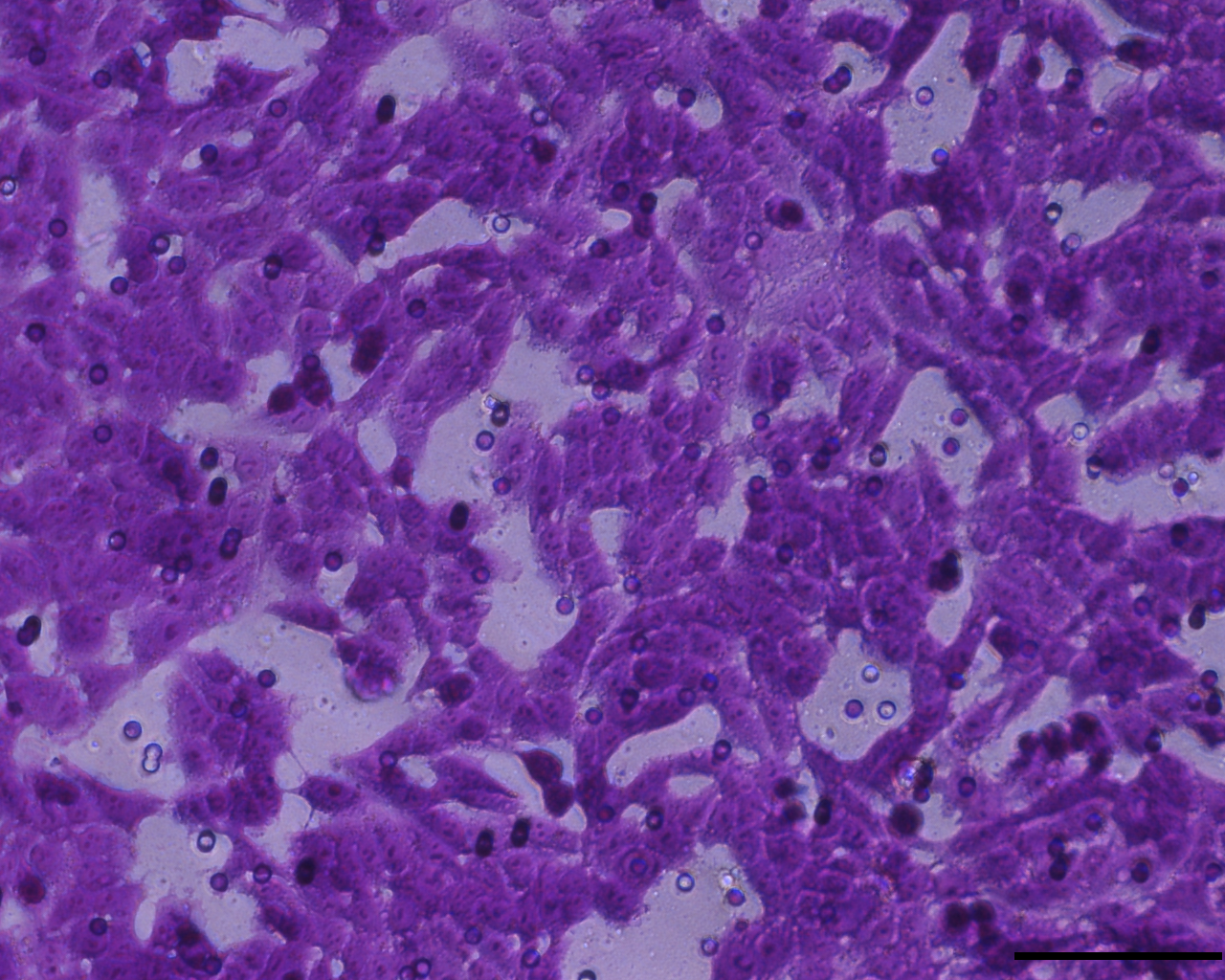

Supplement: Supplementary file 6 [file DataSheet_6.zip › Figure 3D/A375 siNC.tif]

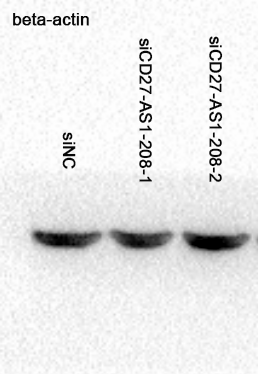

Supplement: Supplementary file 7 [file DataSheet_7.zip › ROW DATA Figure 4/Figure 4A/A2058 beta-actin.tif]

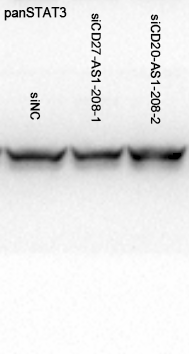

Supplement: Supplementary file 7 [file DataSheet_7.zip › ROW DATA Figure 4/Figure 4A/A2058 panSTAT3.tif]

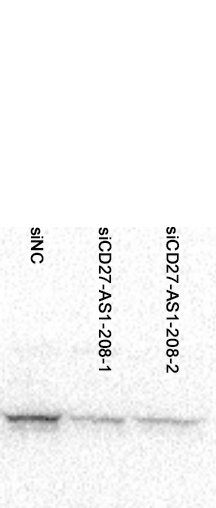

Supplement: Supplementary file 7 [file DataSheet_7.zip › ROW DATA Figure 4/Figure 4A/A2058 pSTAT3(Y705).tif]

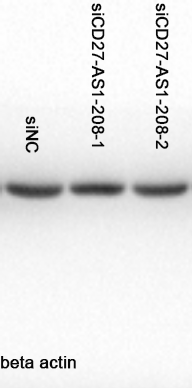

Supplement: Supplementary file 7 [file DataSheet_7.zip › ROW DATA Figure 4/Figure 4B/A375 beta-actin.tif]

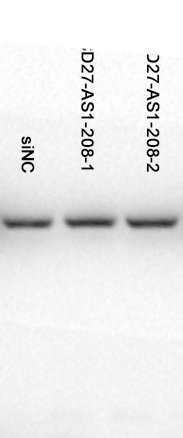

Supplement: Supplementary file 7 [file DataSheet_7.zip › ROW DATA Figure 4/Figure 4B/A375 panSTAT3.tif]

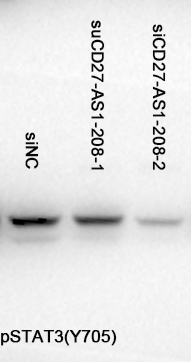

Supplement: Supplementary file 7 [file DataSheet_7.zip › ROW DATA Figure 4/Figure 4B/A375 pSTAT3(Y705).tif]

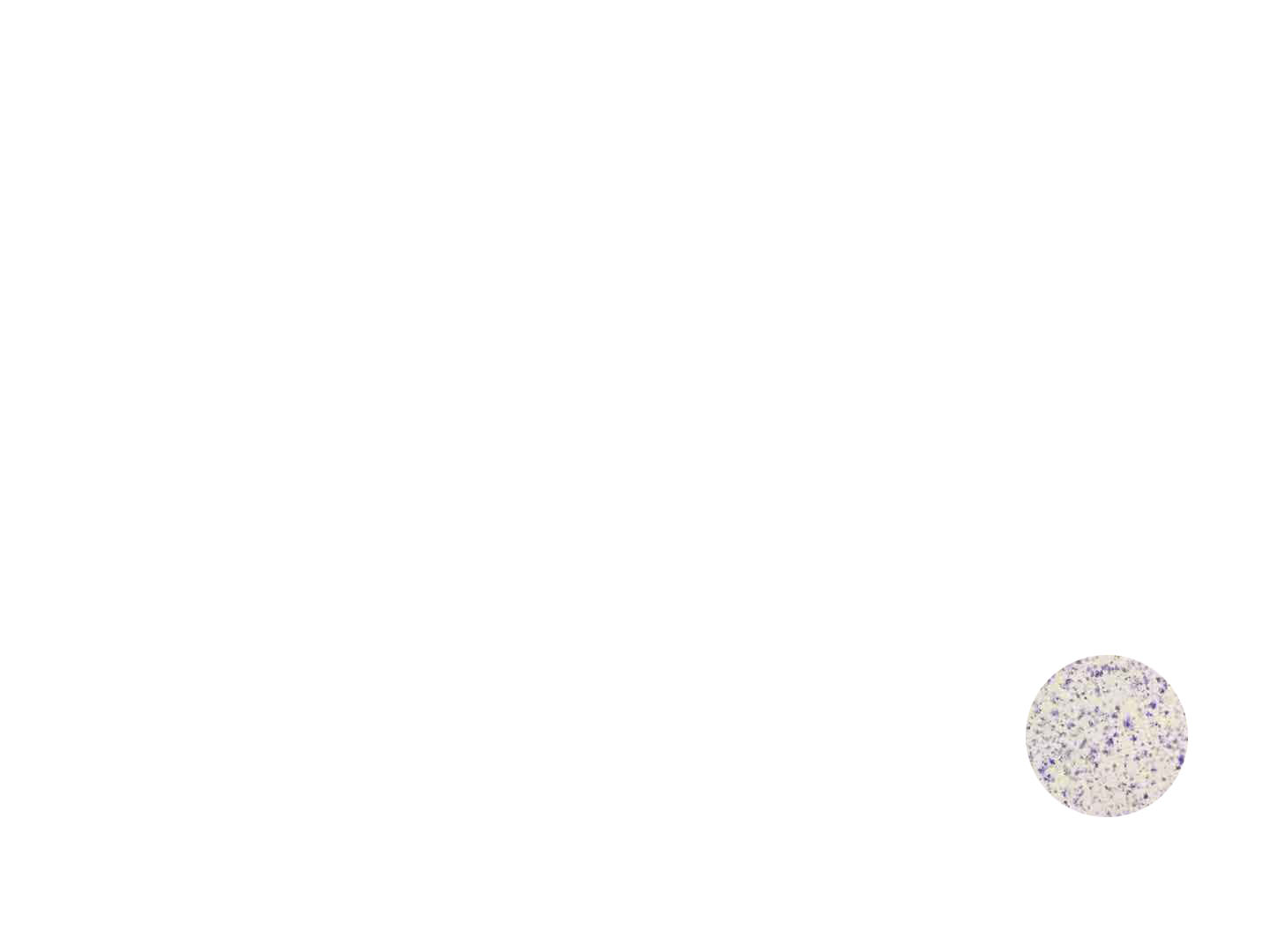

Supplement: Supplementary file 7 [file DataSheet_7.zip › ROW DATA Figure 4/Figure 4D/A2058 siCD27-AS1-208+IL6.jpg]

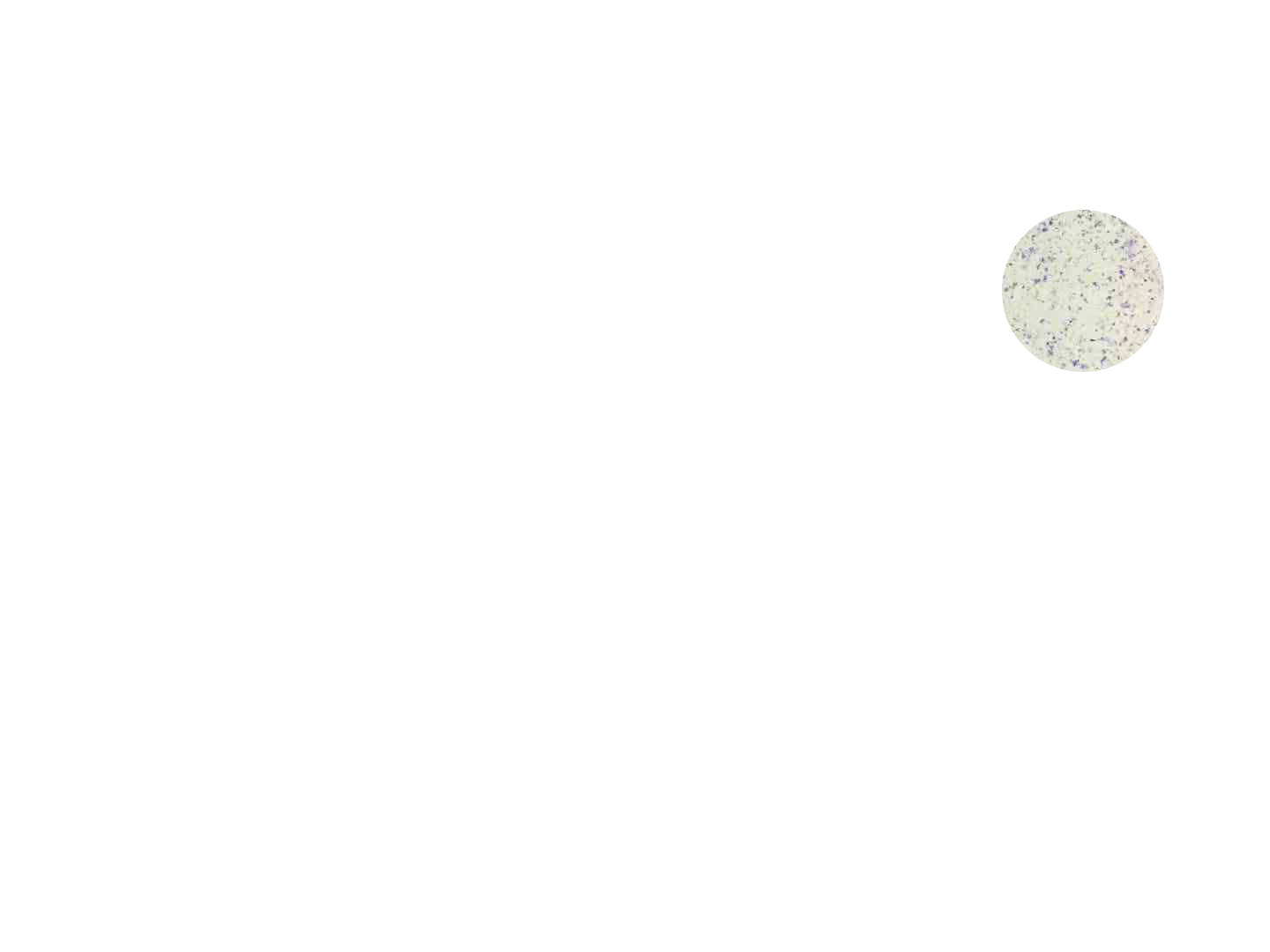

Supplement: Supplementary file 7 [file DataSheet_7.zip › ROW DATA Figure 4/Figure 4D/A2058 siCD27-AS1-208.jpg]

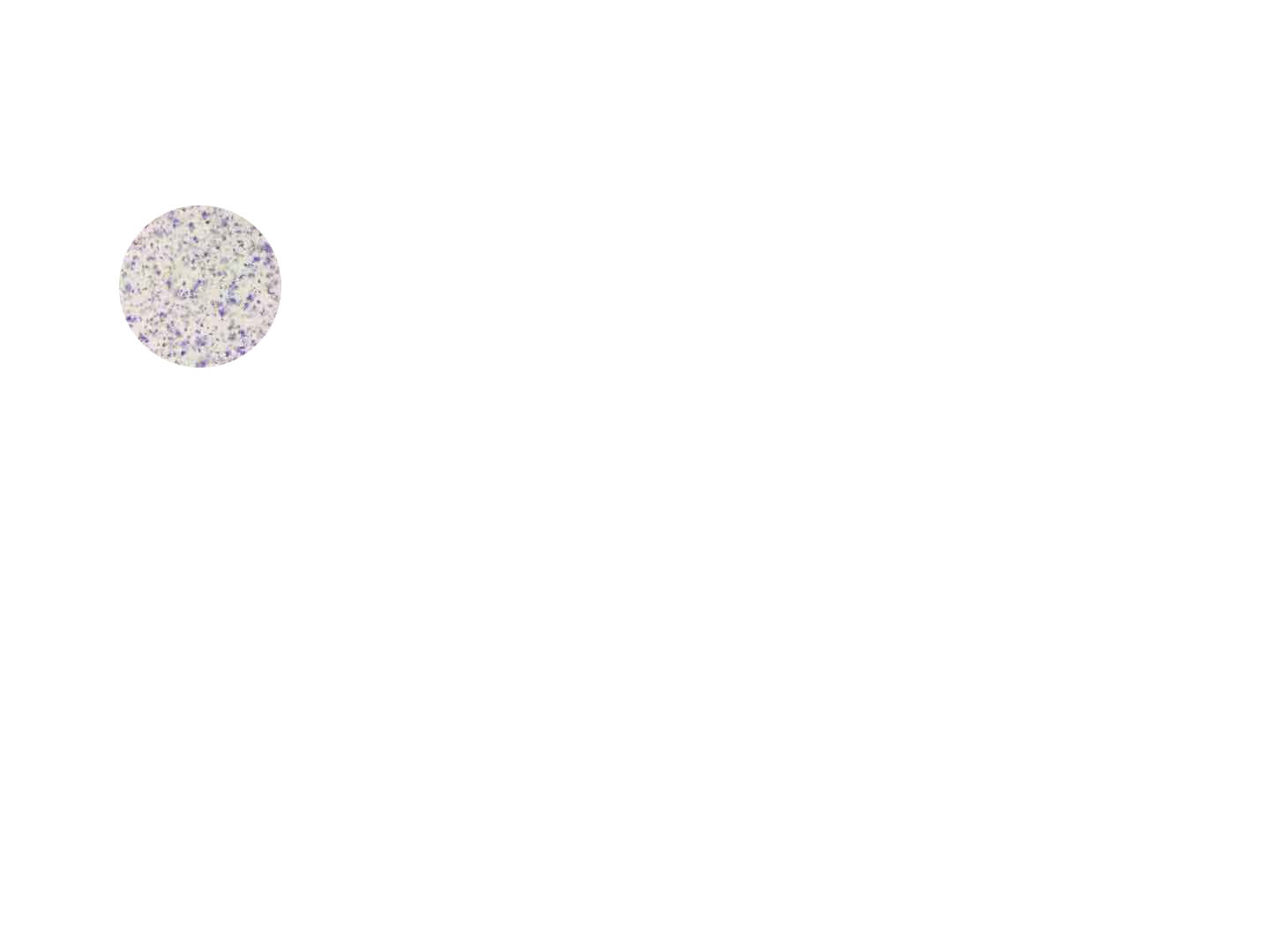

Supplement: Supplementary file 7 [file DataSheet_7.zip › ROW DATA Figure 4/Figure 4D/A2058 siNC.jpg]

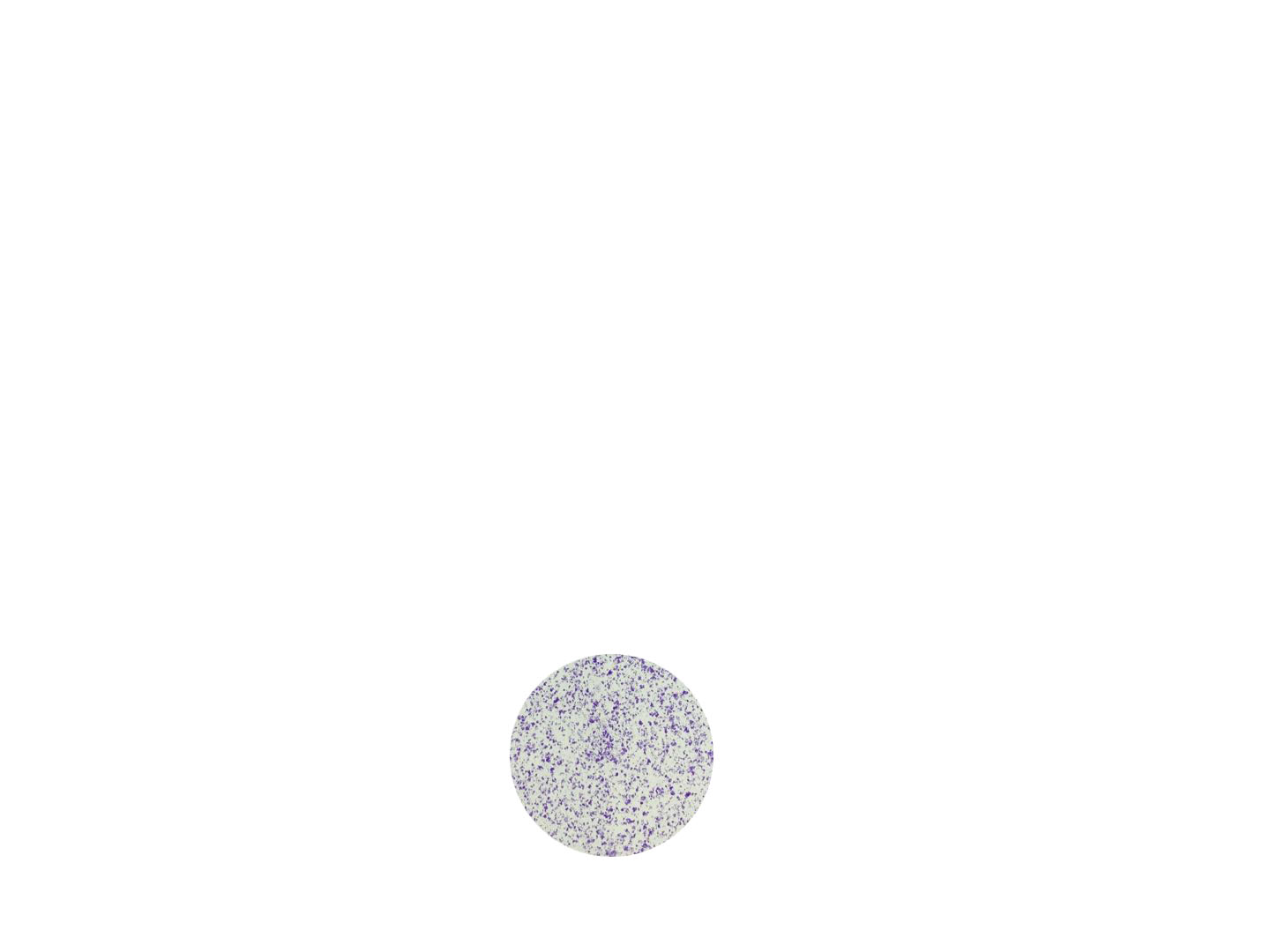

Supplement: Supplementary file 7 [file DataSheet_7.zip › ROW DATA Figure 4/Figure 4D/A375 siCD27-AS1-208+IL6.jpg]

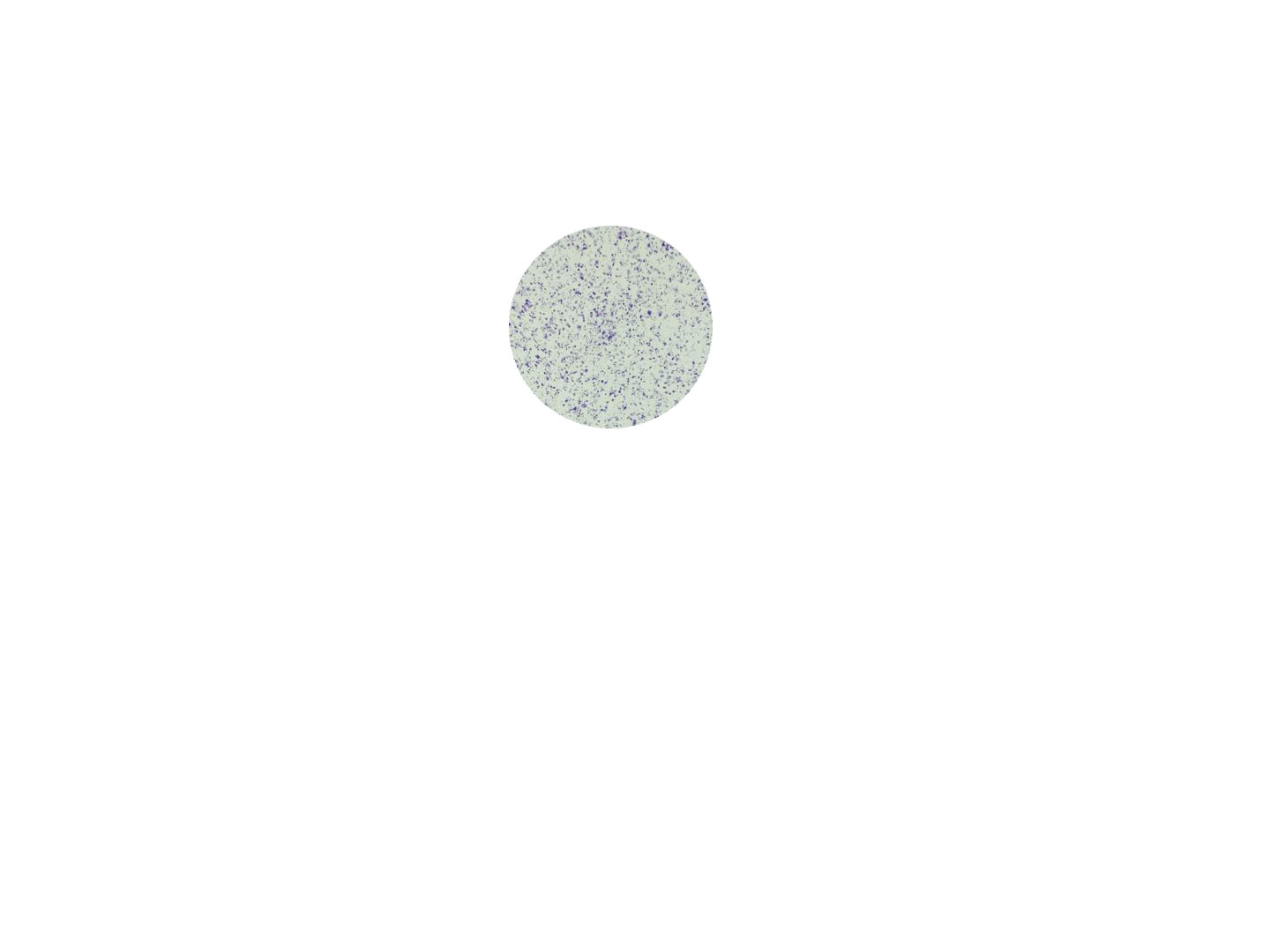

Supplement: Supplementary file 7 [file DataSheet_7.zip › ROW DATA Figure 4/Figure 4D/A375 siCD27-AS1-208.jpg]

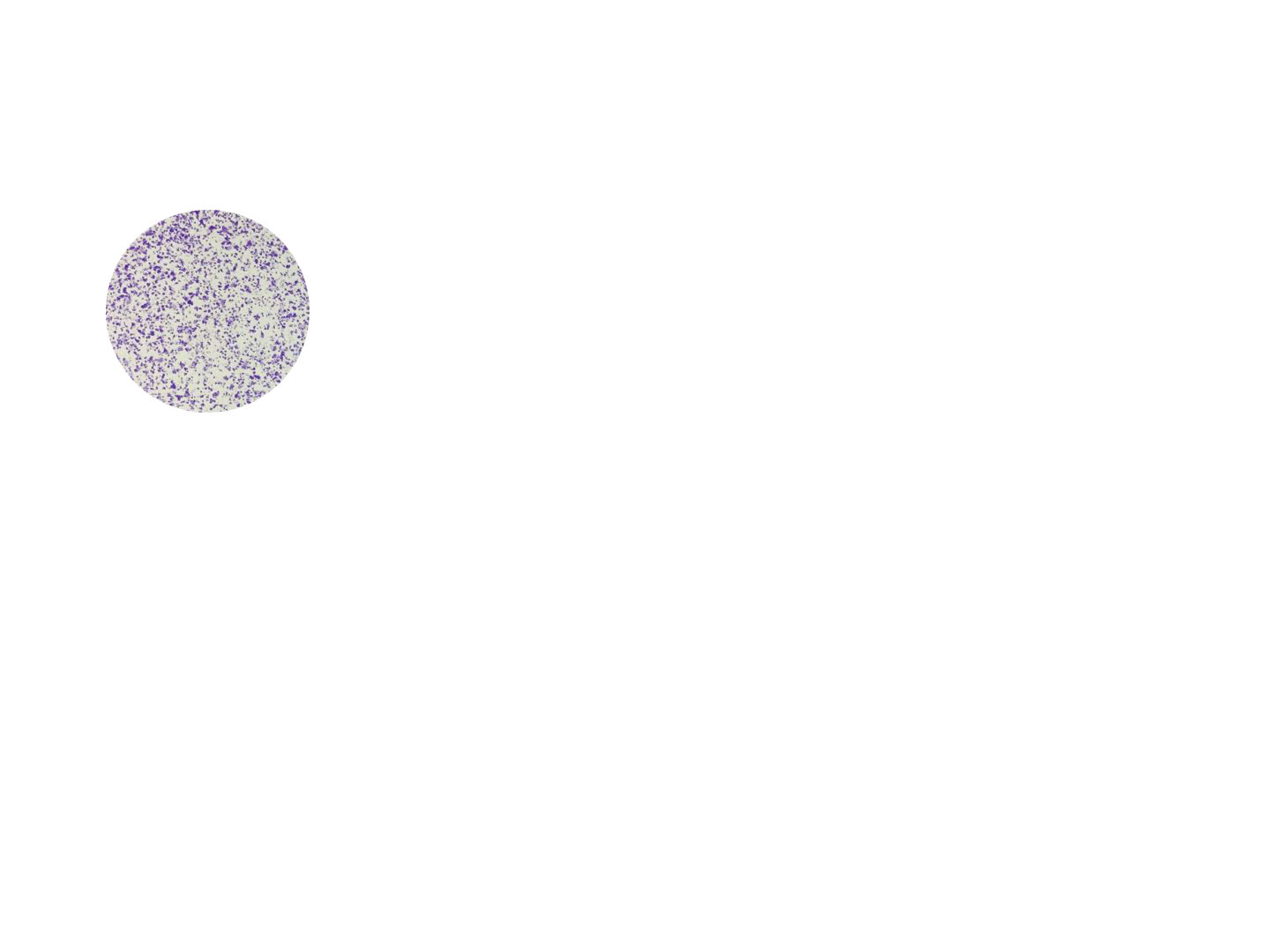

Supplement: Supplementary file 7 [file DataSheet_7.zip › ROW DATA Figure 4/Figure 4D/A375 siNC.jpg]

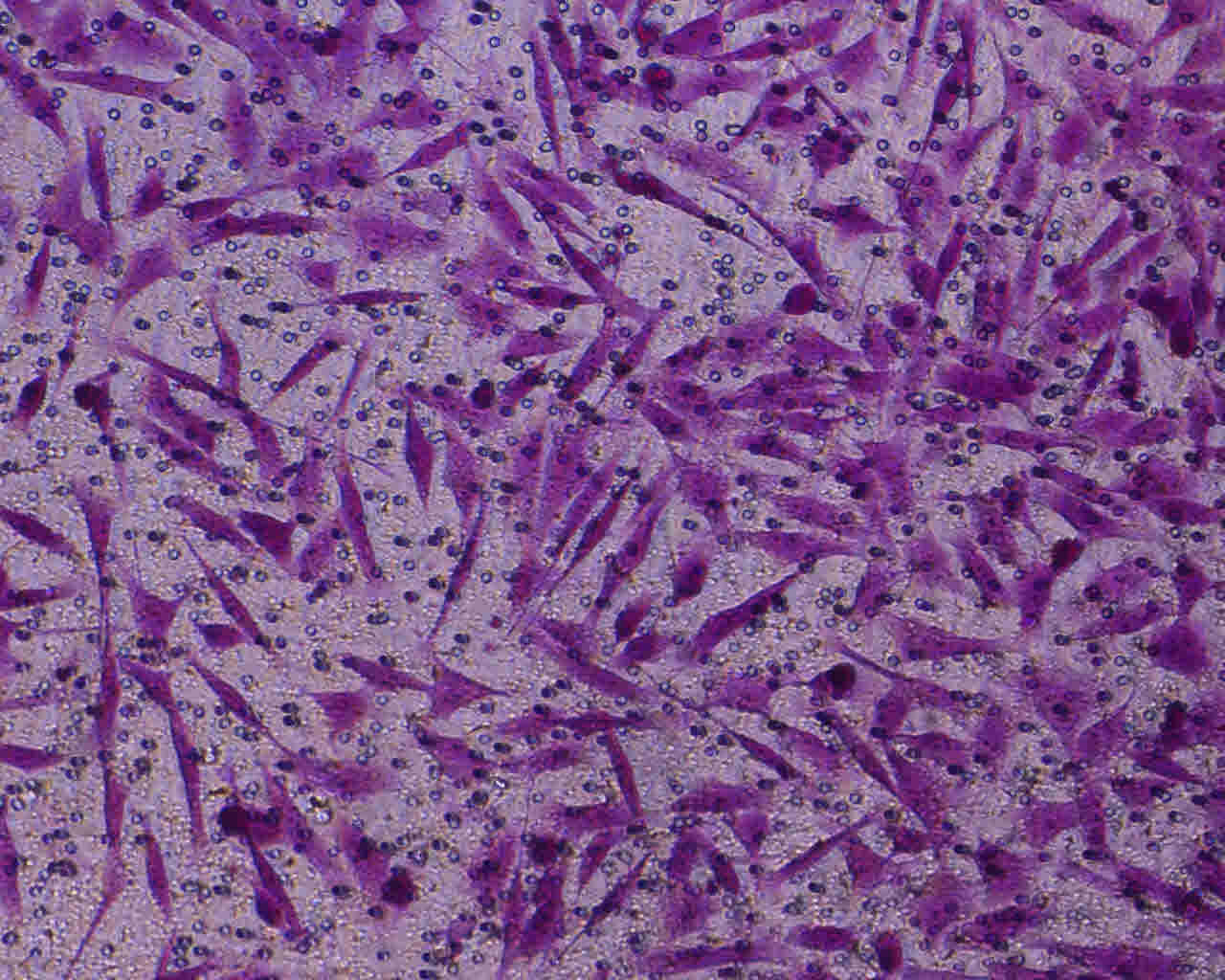

Supplement: Supplementary file 7 [file DataSheet_7.zip › ROW DATA Figure 4/Figure 4E/A2058 siCD27-AS1-208+IL6.jpg]

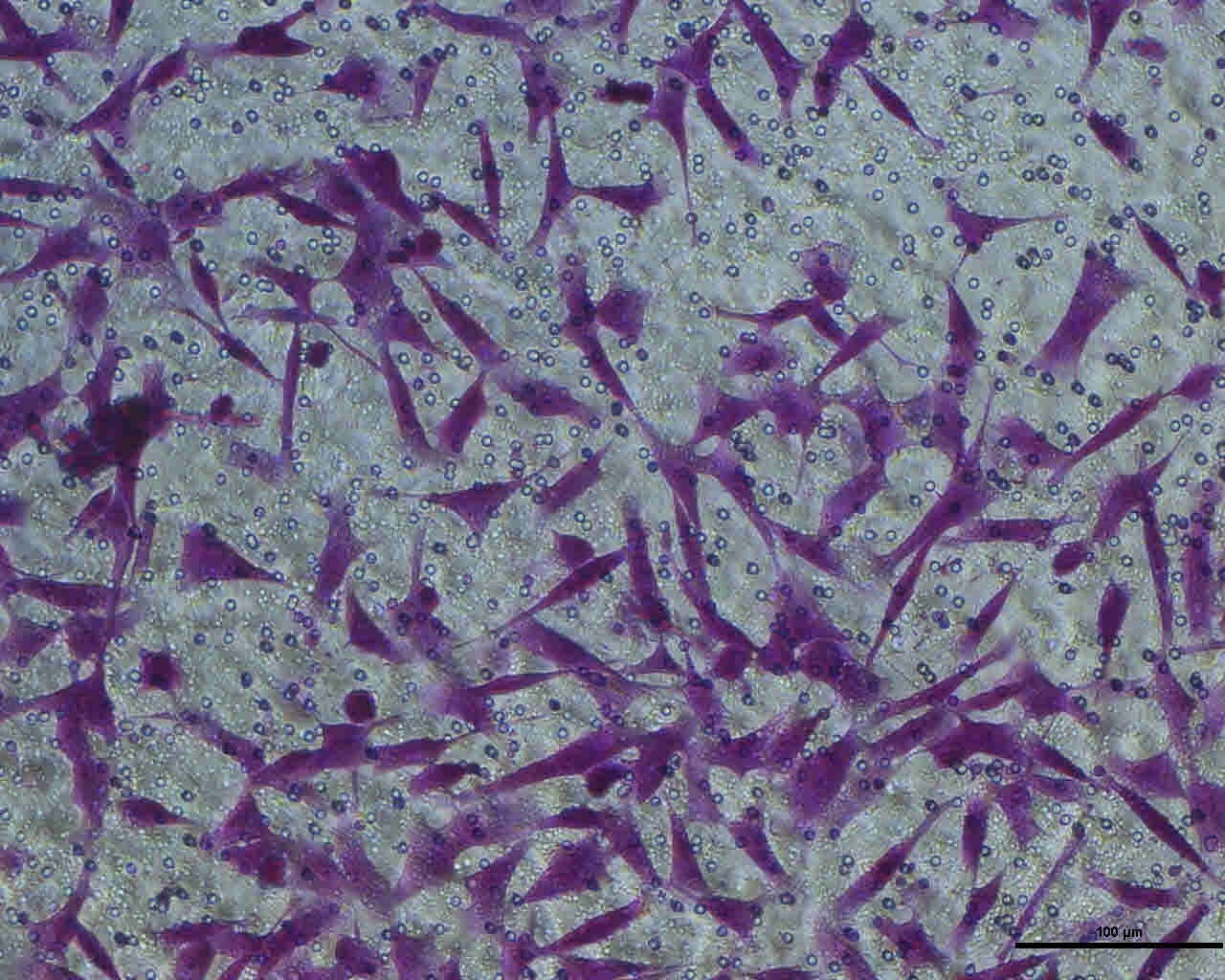

Supplement: Supplementary file 7 [file DataSheet_7.zip › ROW DATA Figure 4/Figure 4E/A2058 siCD27-AS1-208.jpg]

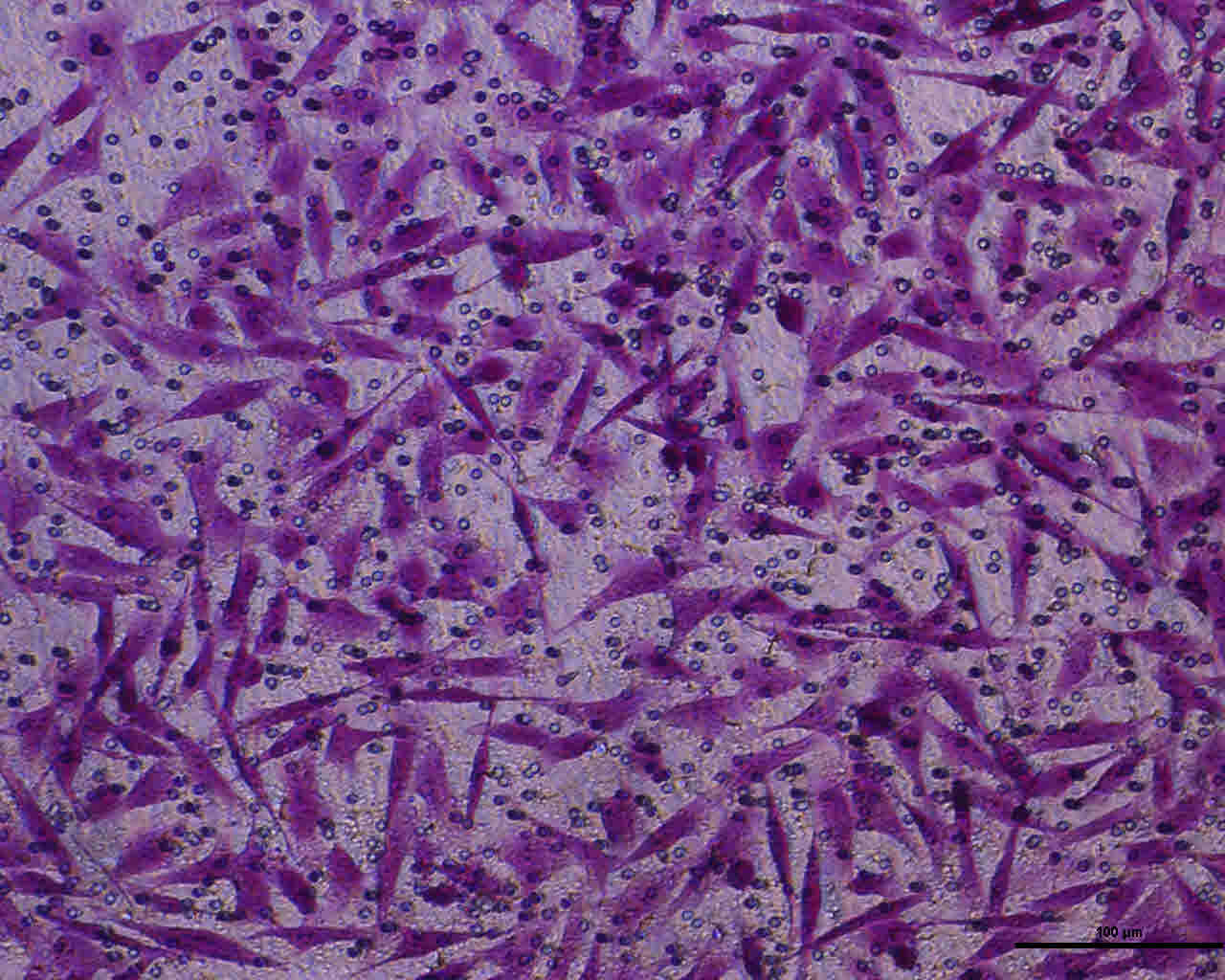

Supplement: Supplementary file 7 [file DataSheet_7.zip › ROW DATA Figure 4/Figure 4E/A2058 siNC.jpg]

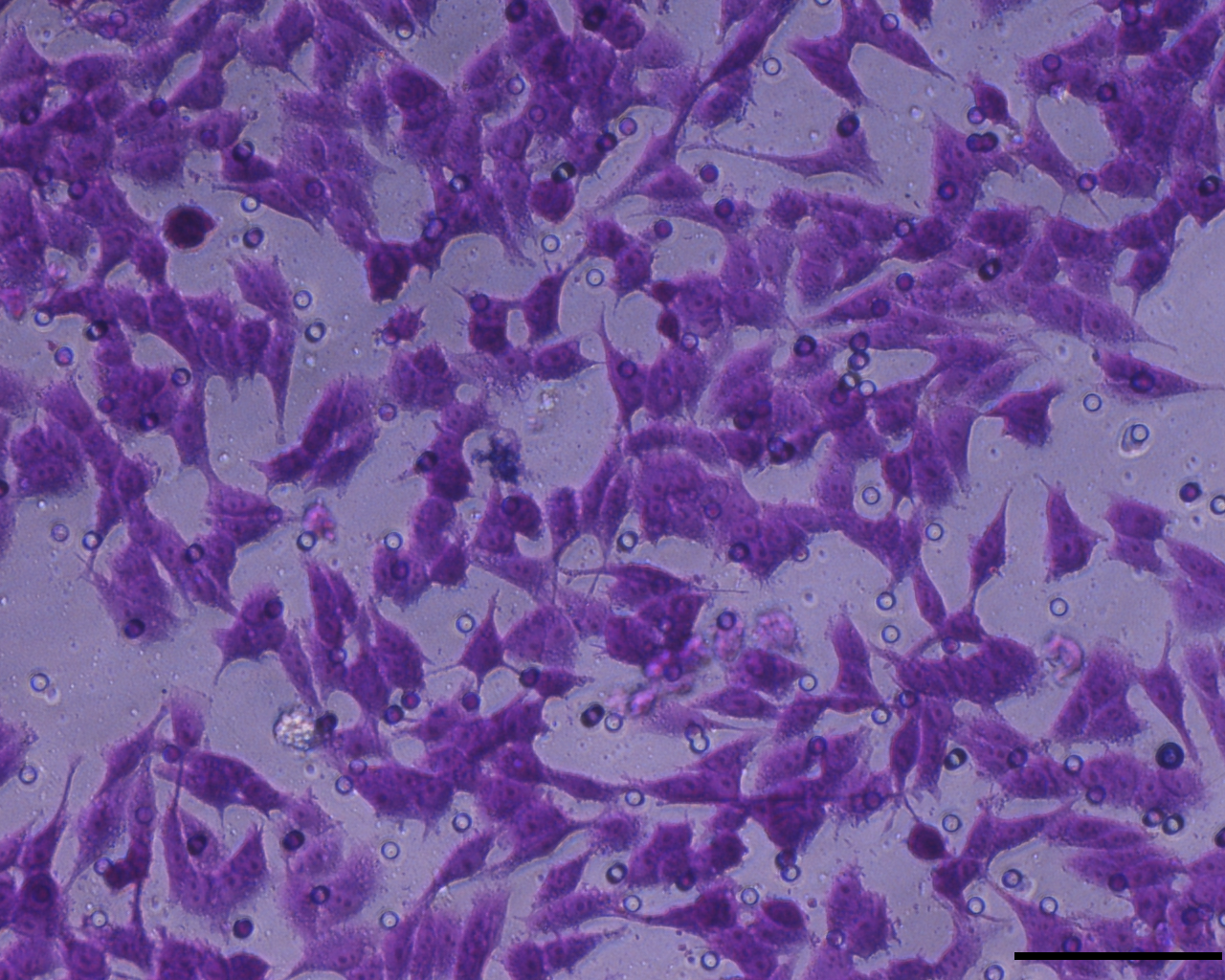

Supplement: Supplementary file 7 [file DataSheet_7.zip › ROW DATA Figure 4/Figure 4E/A375 siCD27-AS1-208+IL6.tif]

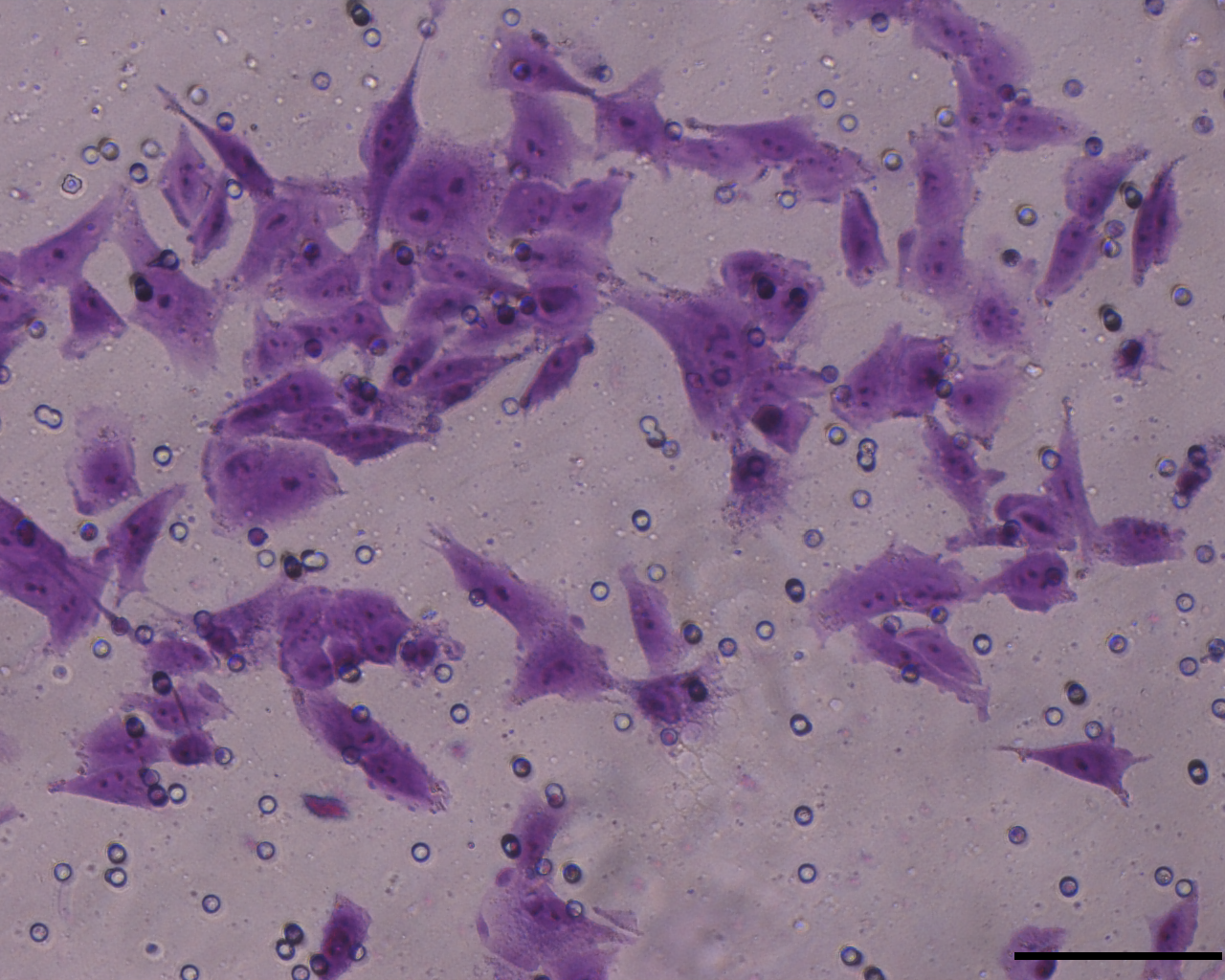

Supplement: Supplementary file 7 [file DataSheet_7.zip › ROW DATA Figure 4/Figure 4E/A375 siCD27-AS1-208.tif]

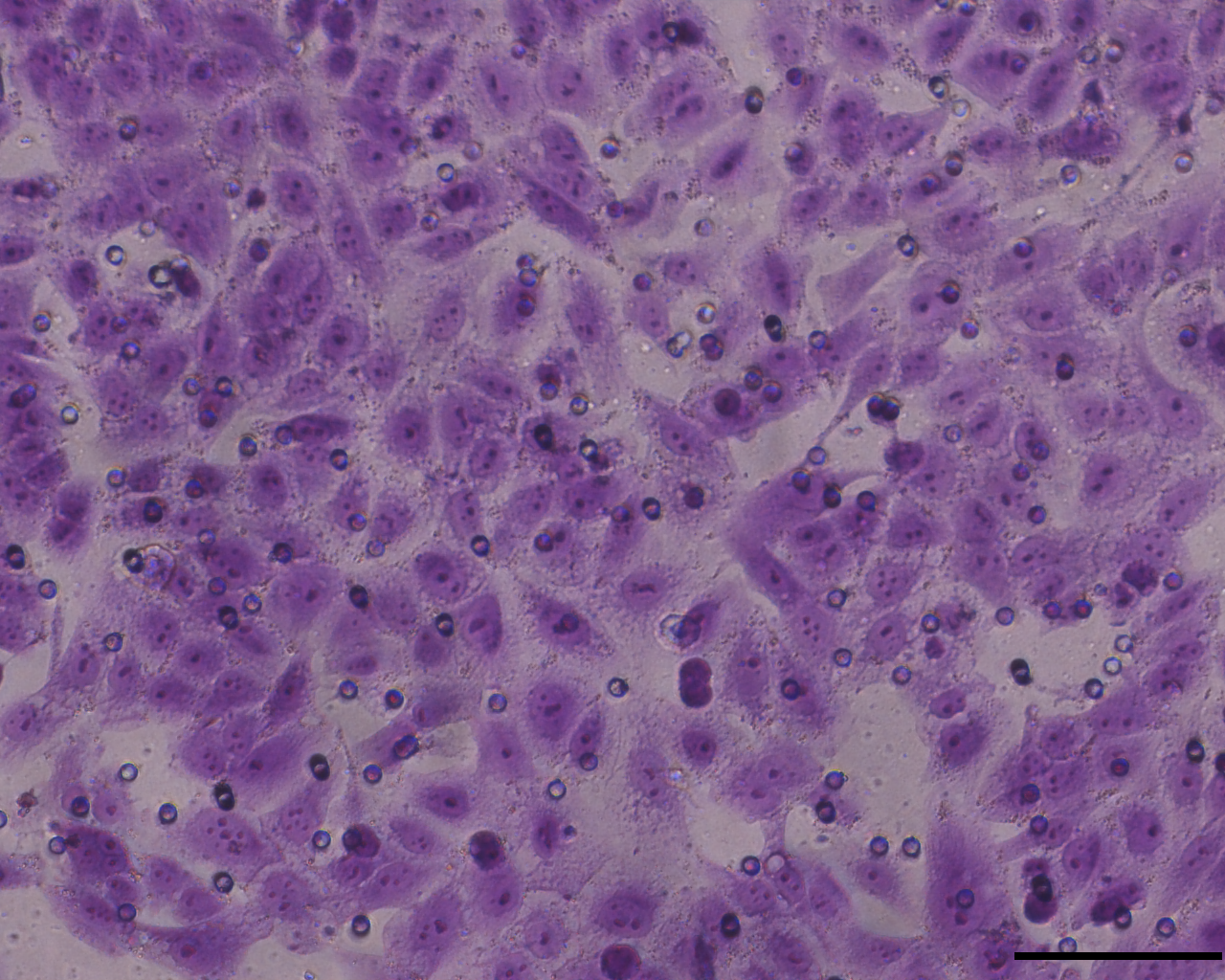

Supplement: Supplementary file 7 [file DataSheet_7.zip › ROW DATA Figure 4/Figure 4E/A375 siNC.tif]

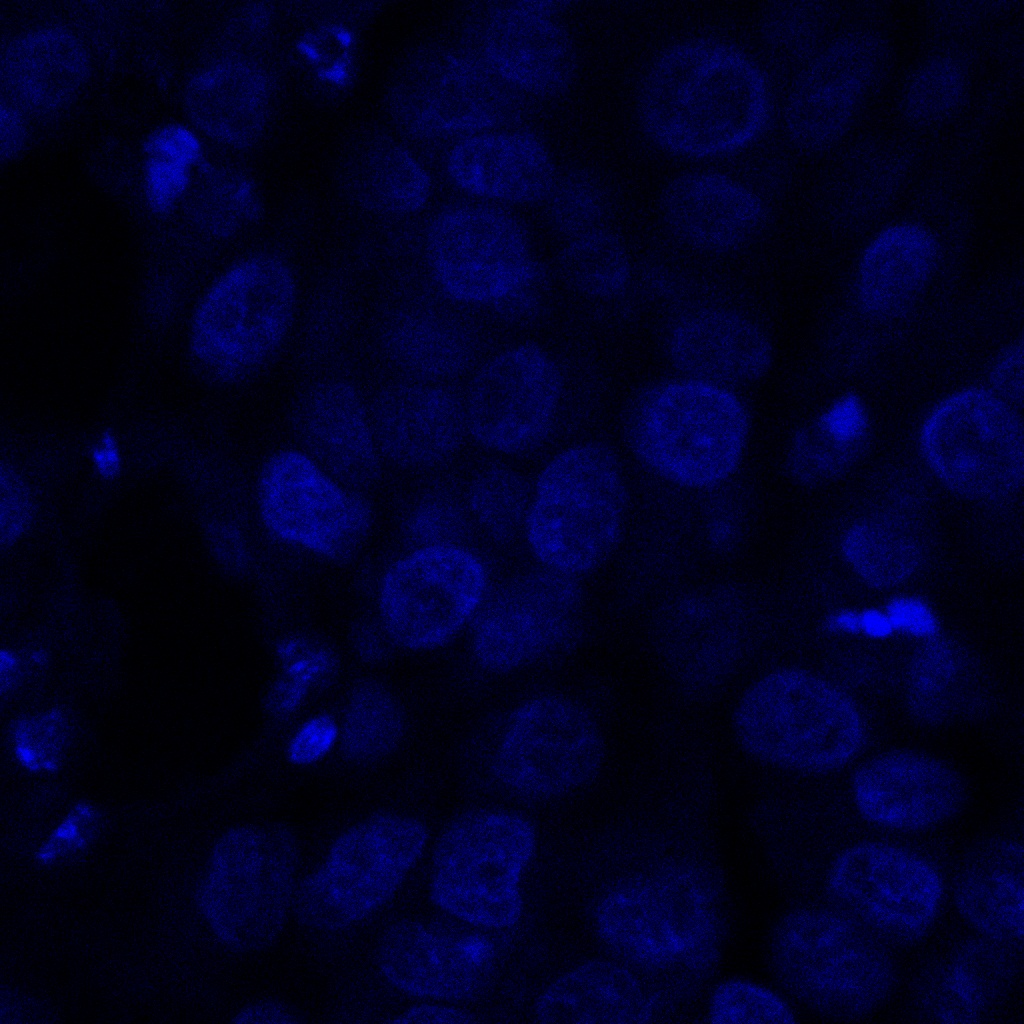

Supplement: Supplementary file 8 [file DataSheet_8.zip › ROW DATA Figure 5/Figure 5D/shCD27-AS1-208 DAPI.jpg]

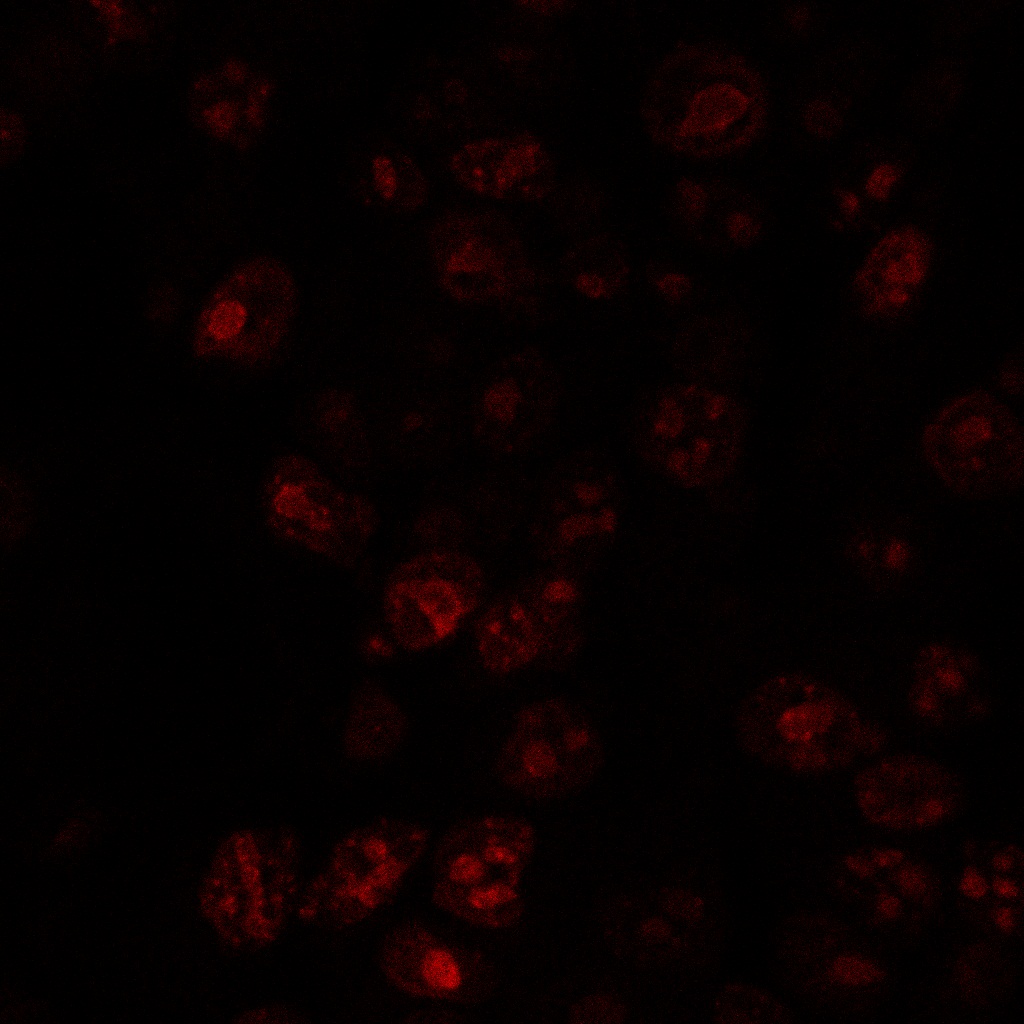

Supplement: Supplementary file 8 [file DataSheet_8.zip › ROW DATA Figure 5/Figure 5D/shCD27-AS1-208 Ki67.jpg]

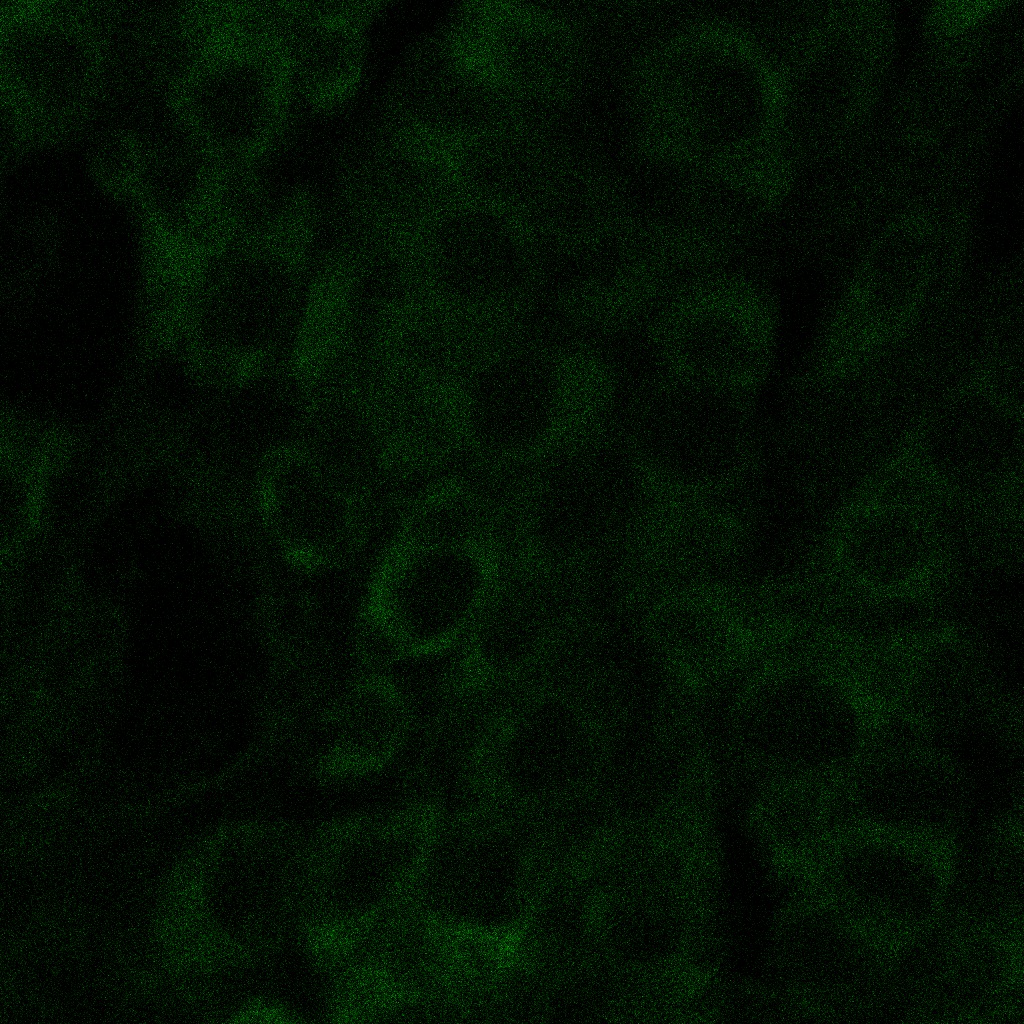

Supplement: Supplementary file 8 [file DataSheet_8.zip › ROW DATA Figure 5/Figure 5D/shCD27-AS1-208 MelanA.jpg]

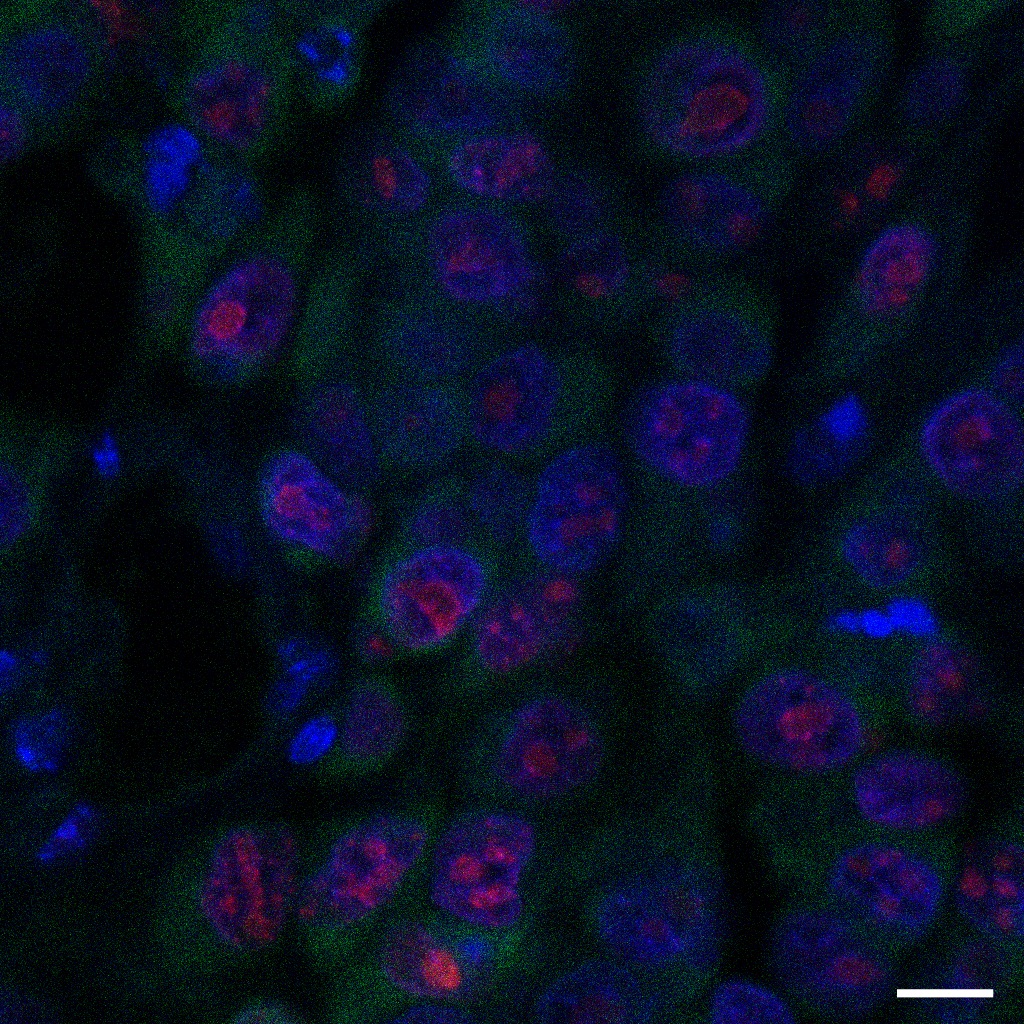

Supplement: Supplementary file 8 [file DataSheet_8.zip › ROW DATA Figure 5/Figure 5D/shCD27-AS1-208 Merge.jpg]

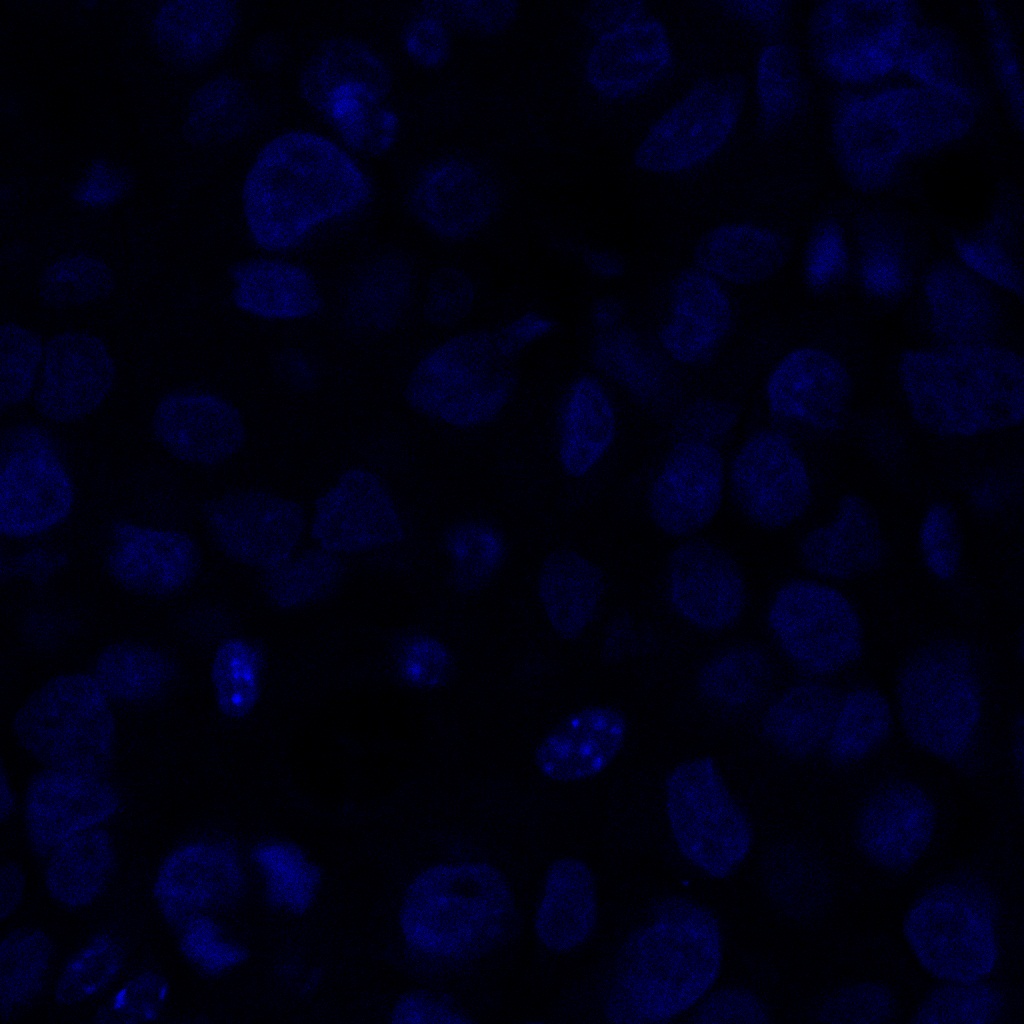

Supplement: Supplementary file 8 [file DataSheet_8.zip › ROW DATA Figure 5/Figure 5D/shNC DAPI.jpg]

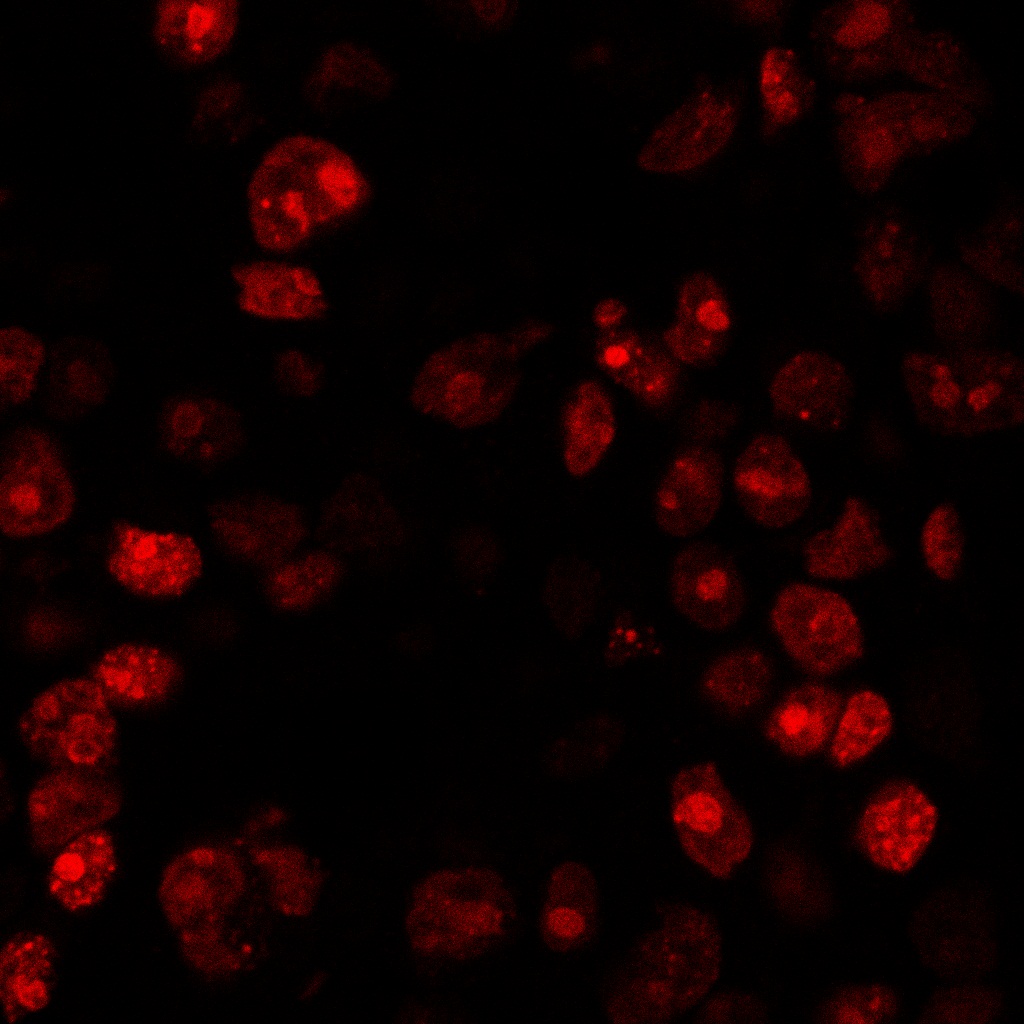

Supplement: Supplementary file 8 [file DataSheet_8.zip › ROW DATA Figure 5/Figure 5D/shNC Ki67.jpg]

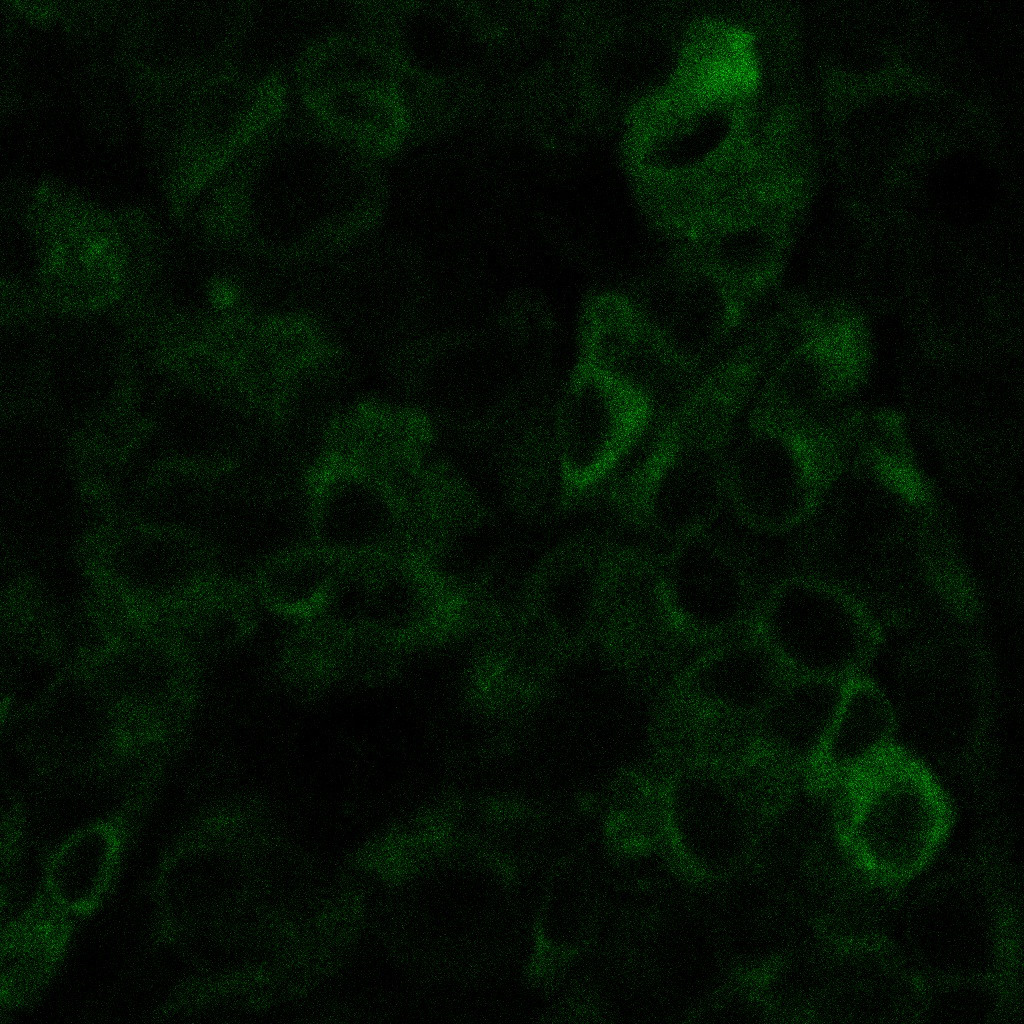

Supplement: Supplementary file 8 [file DataSheet_8.zip › ROW DATA Figure 5/Figure 5D/shNC MelanA.jpg]

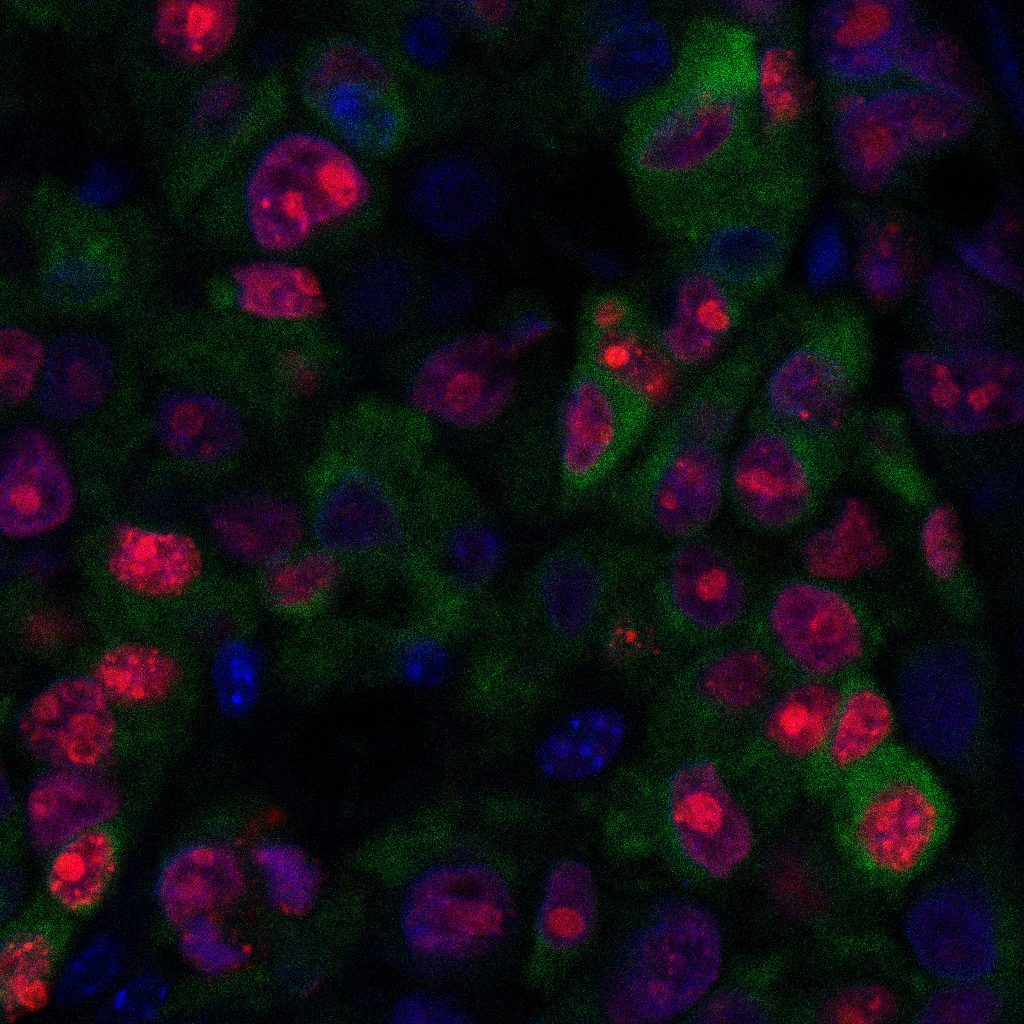

Supplement: Supplementary file 8 [file DataSheet_8.zip › ROW DATA Figure 5/Figure 5D/shNC Merge.jpg]

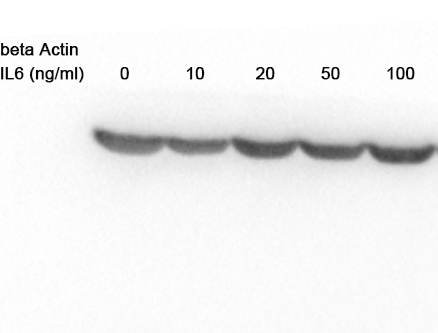

Supplement: Supplementary file 9 [file DataSheet_9.zip › ROW DATA Supplymetary Figures/Supplymentary Figure 10/A2058 beta Actin.tif]

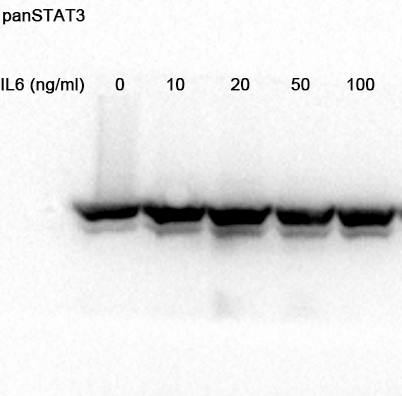

Supplement: Supplementary file 9 [file DataSheet_9.zip › ROW DATA Supplymetary Figures/Supplymentary Figure 10/A2058 panSTAT3.tif]

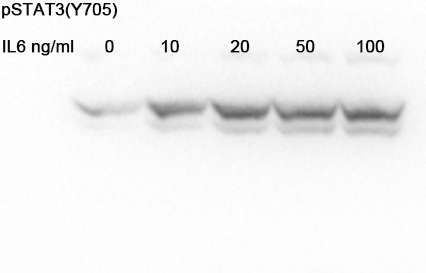

Supplement: Supplementary file 9 [file DataSheet_9.zip › ROW DATA Supplymetary Figures/Supplymentary Figure 10/A2058 pSTAT3 Y705.tif]

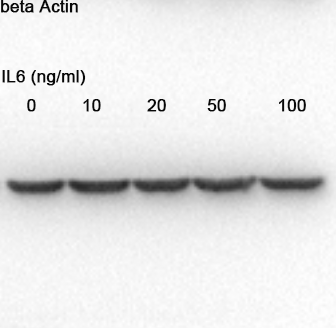

Supplement: Supplementary file 9 [file DataSheet_9.zip › ROW DATA Supplymetary Figures/Supplymentary Figure 10/A375 beta Actin.tif]

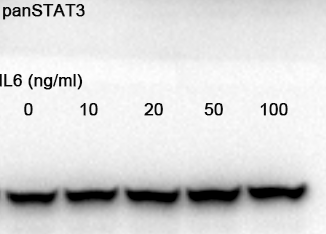

Supplement: Supplementary file 9 [file DataSheet_9.zip › ROW DATA Supplymetary Figures/Supplymentary Figure 10/A375 panSTAT3.tif]

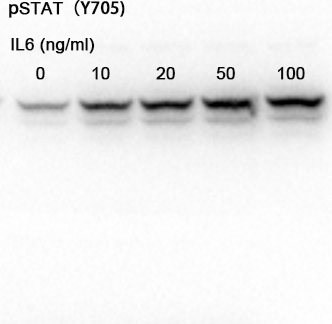

Supplement: Supplementary file 9 [file DataSheet_9.zip › ROW DATA Supplymetary Figures/Supplymentary Figure 10/A375 pSTAT3 Y705.tif]

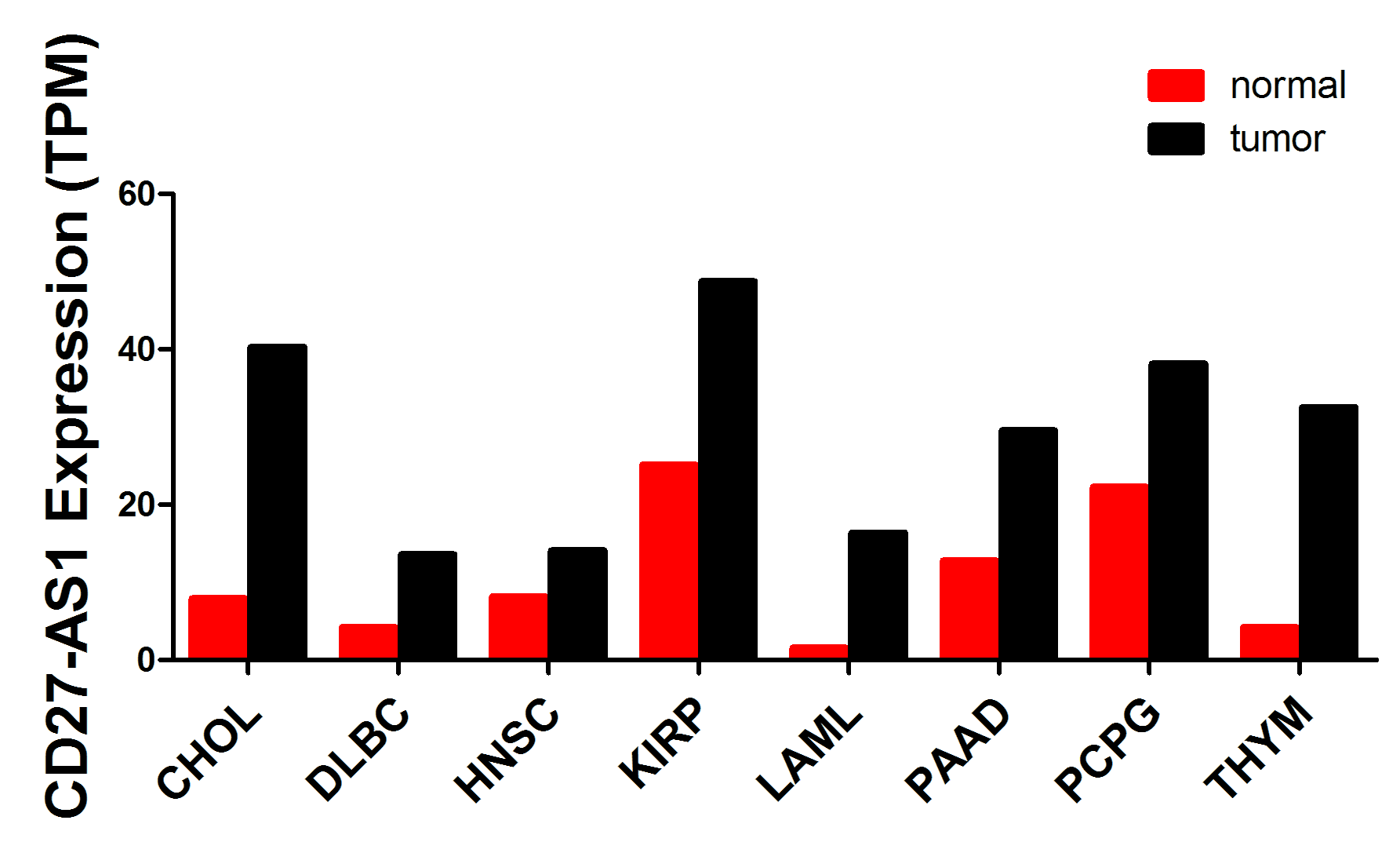

Supplement: Supplementary file 9 [file DataSheet_9.zip › ROW DATA Supplymetary Figures/Supplymentary Figure 3.tif]

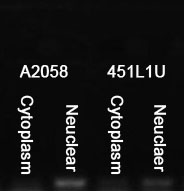

Supplement: Supplementary file 9 [file DataSheet_9.zip › ROW DATA Supplymetary Figures/Supplymentary Figure 4.jpg]

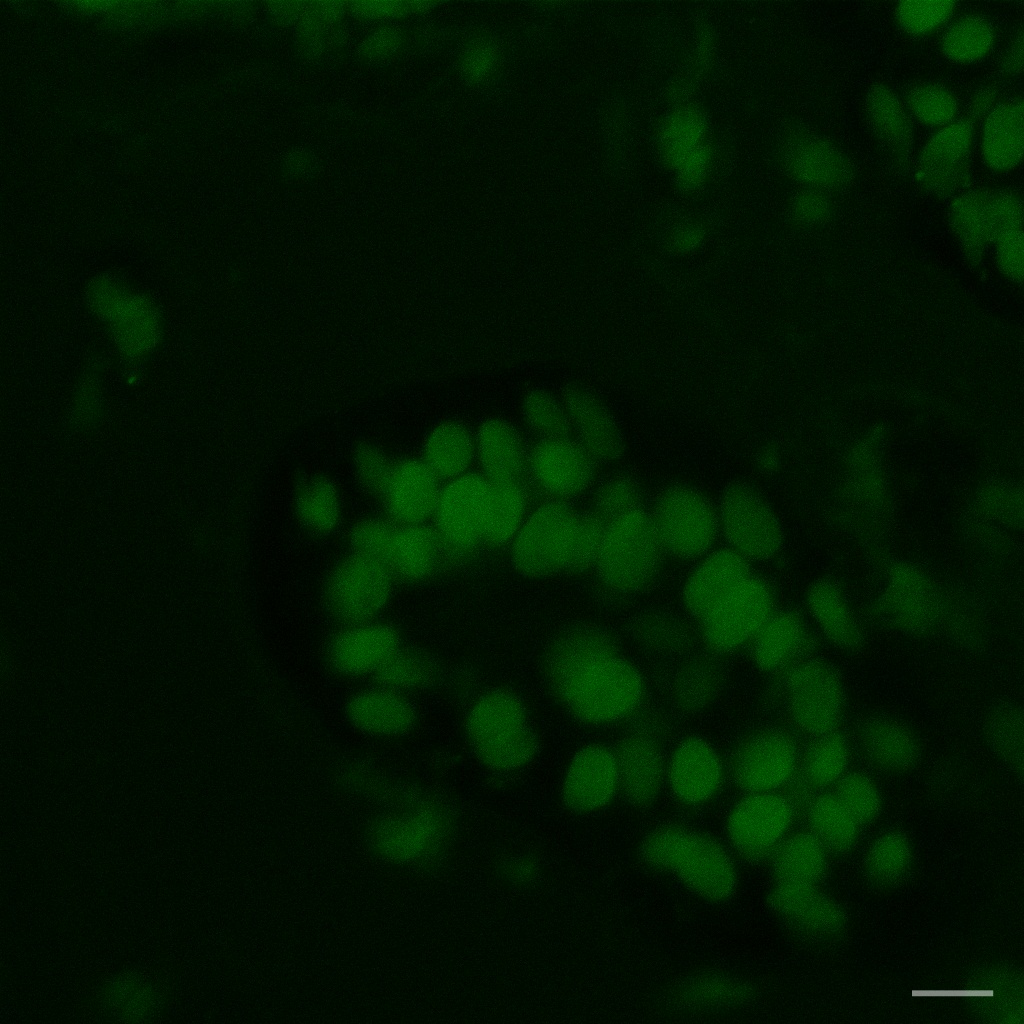

Supplement: Supplementary file 9 [file DataSheet_9.zip › ROW DATA Supplymetary Figures/Supplymentary Figure 5/Supplymentary Figure 5A/Supplymentary Figure 5A CD27-AS1-208.jpg]

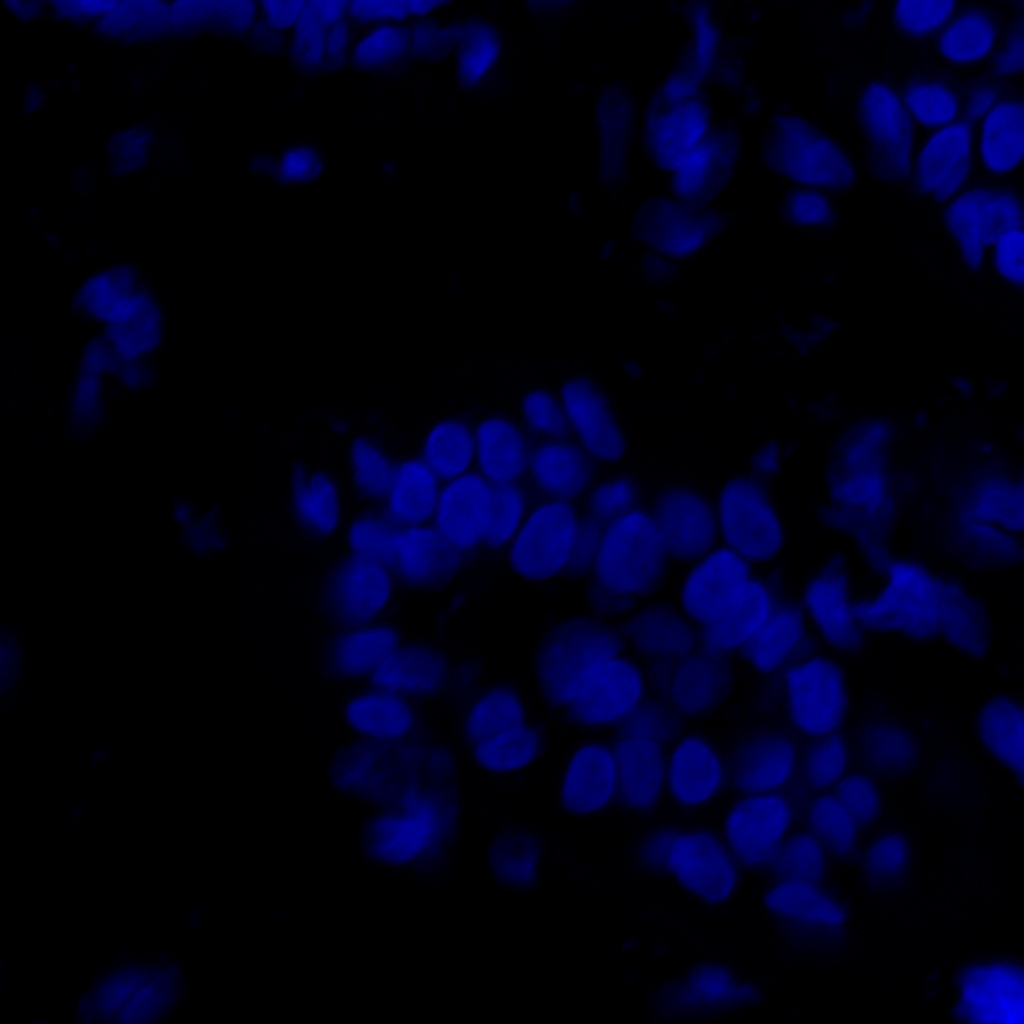

Supplement: Supplementary file 9 [file DataSheet_9.zip › ROW DATA Supplymetary Figures/Supplymentary Figure 5/Supplymentary Figure 5A/Supplymentary Figure 5A DAPI.jpg]

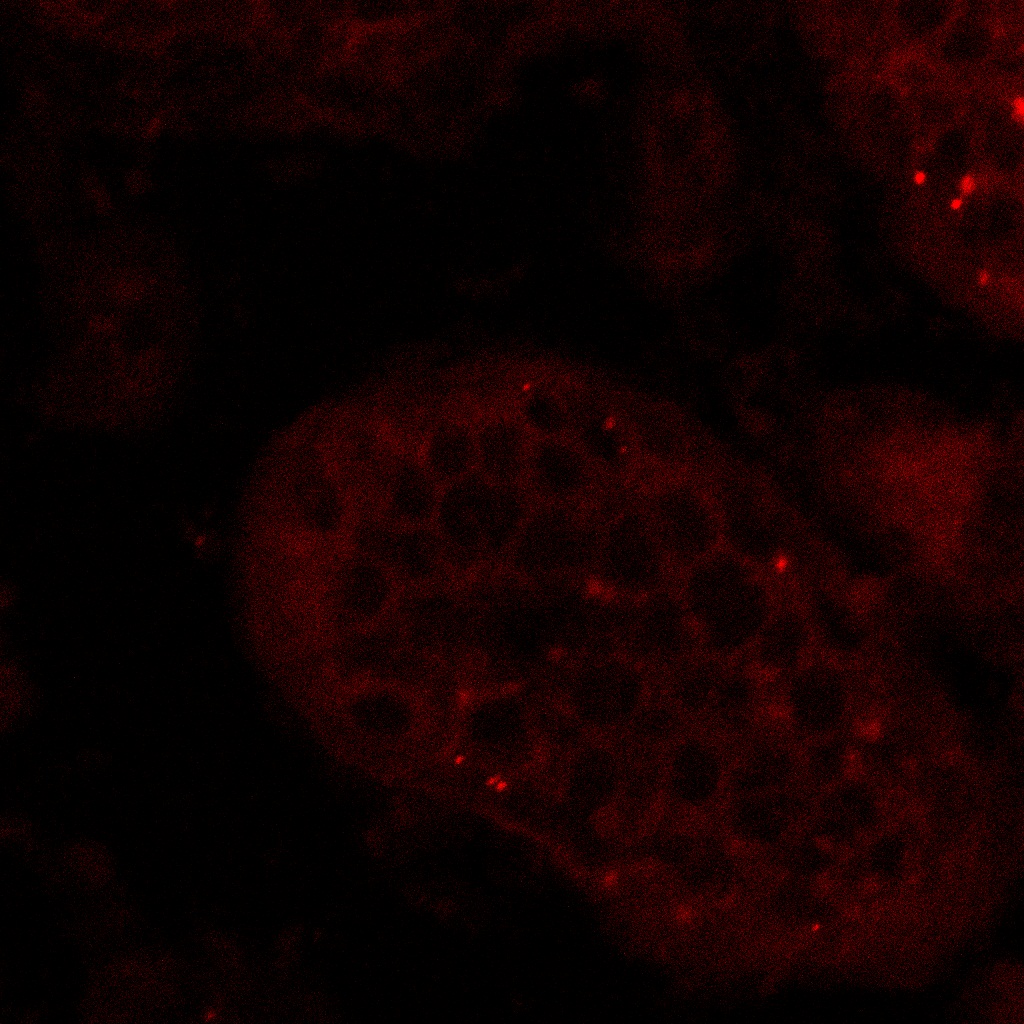

Supplement: Supplementary file 9 [file DataSheet_9.zip › ROW DATA Supplymetary Figures/Supplymentary Figure 5/Supplymentary Figure 5A/Supplymentary Figure 5A Melan A.jpg]

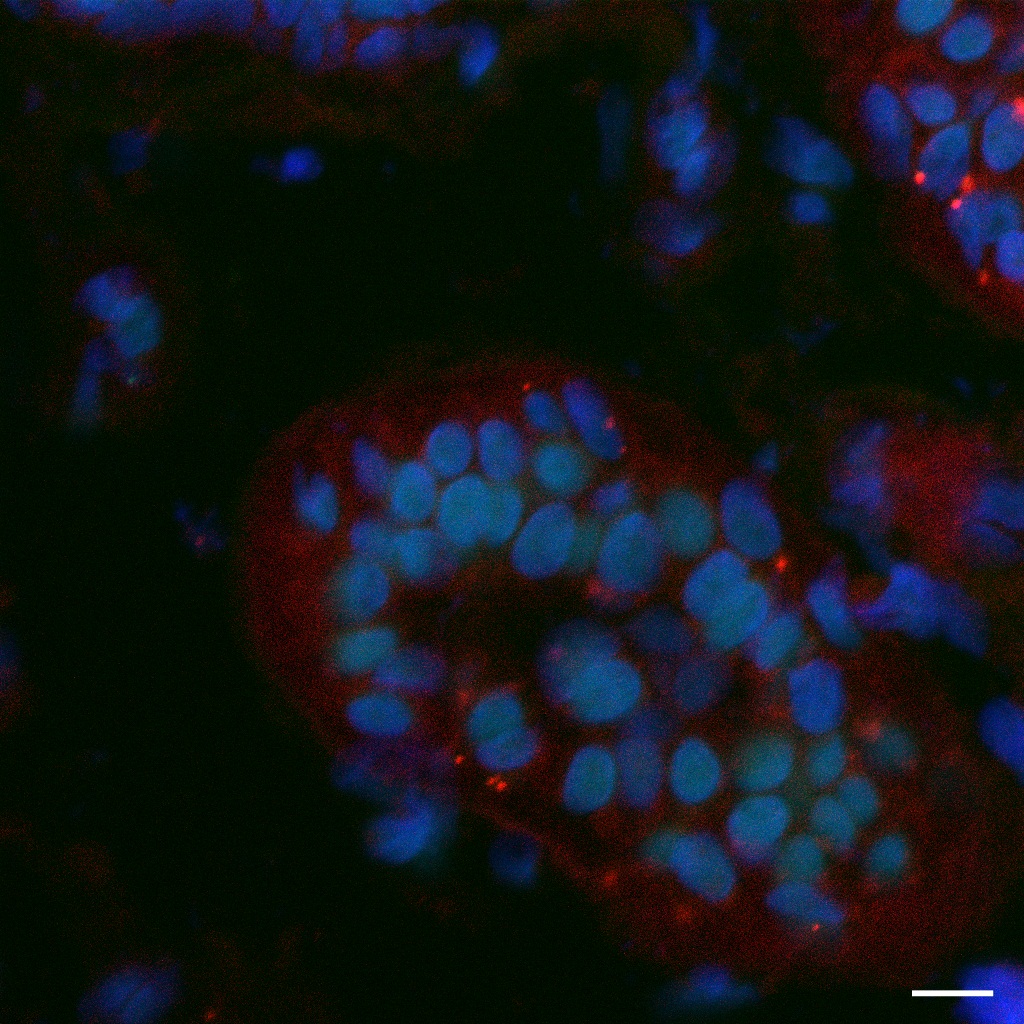

Supplement: Supplementary file 9 [file DataSheet_9.zip › ROW DATA Supplymetary Figures/Supplymentary Figure 5/Supplymentary Figure 5A/Supplymentary Figure 5A Merge.jpg]

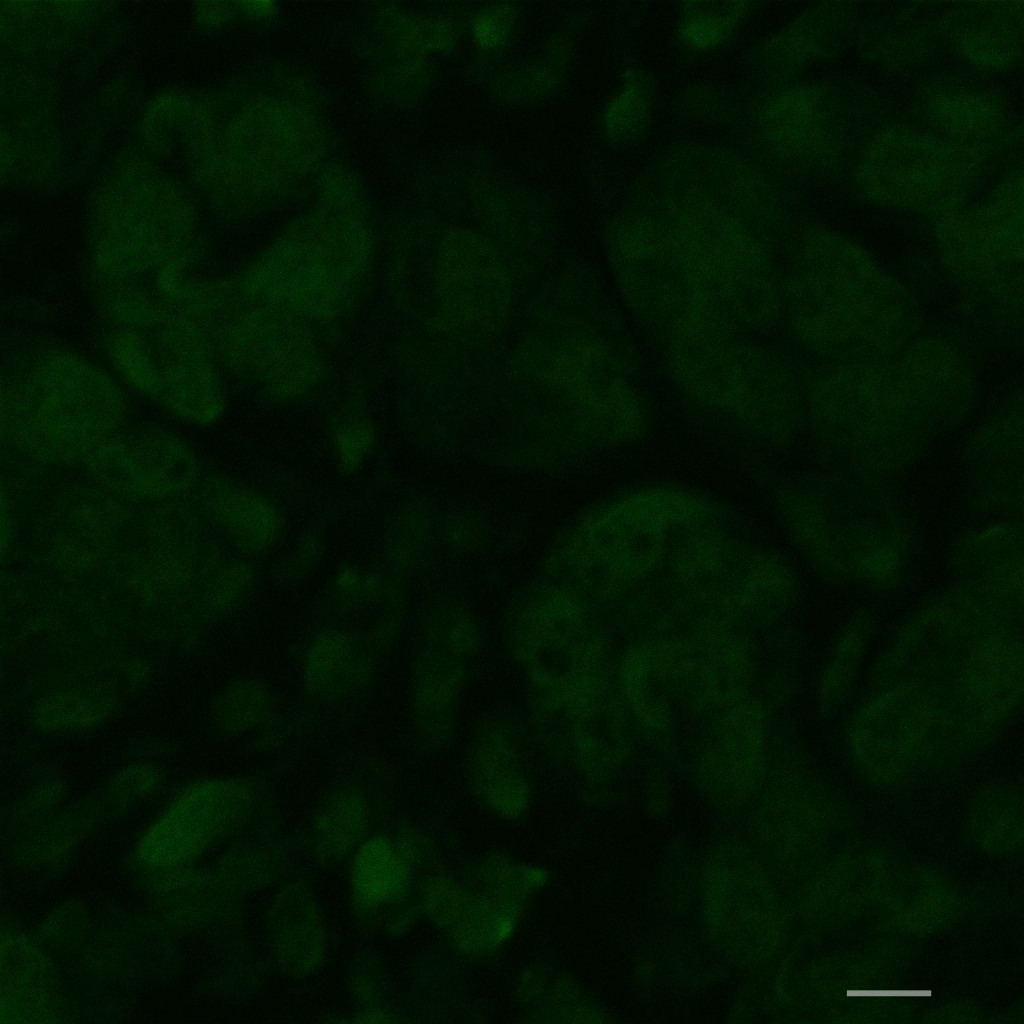

Supplement: Supplementary file 9 [file DataSheet_9.zip › ROW DATA Supplymetary Figures/Supplymentary Figure 5/Supplymentary Figure 5B/Supplymentary Figure 5B CD27-AS1-208.jpg]

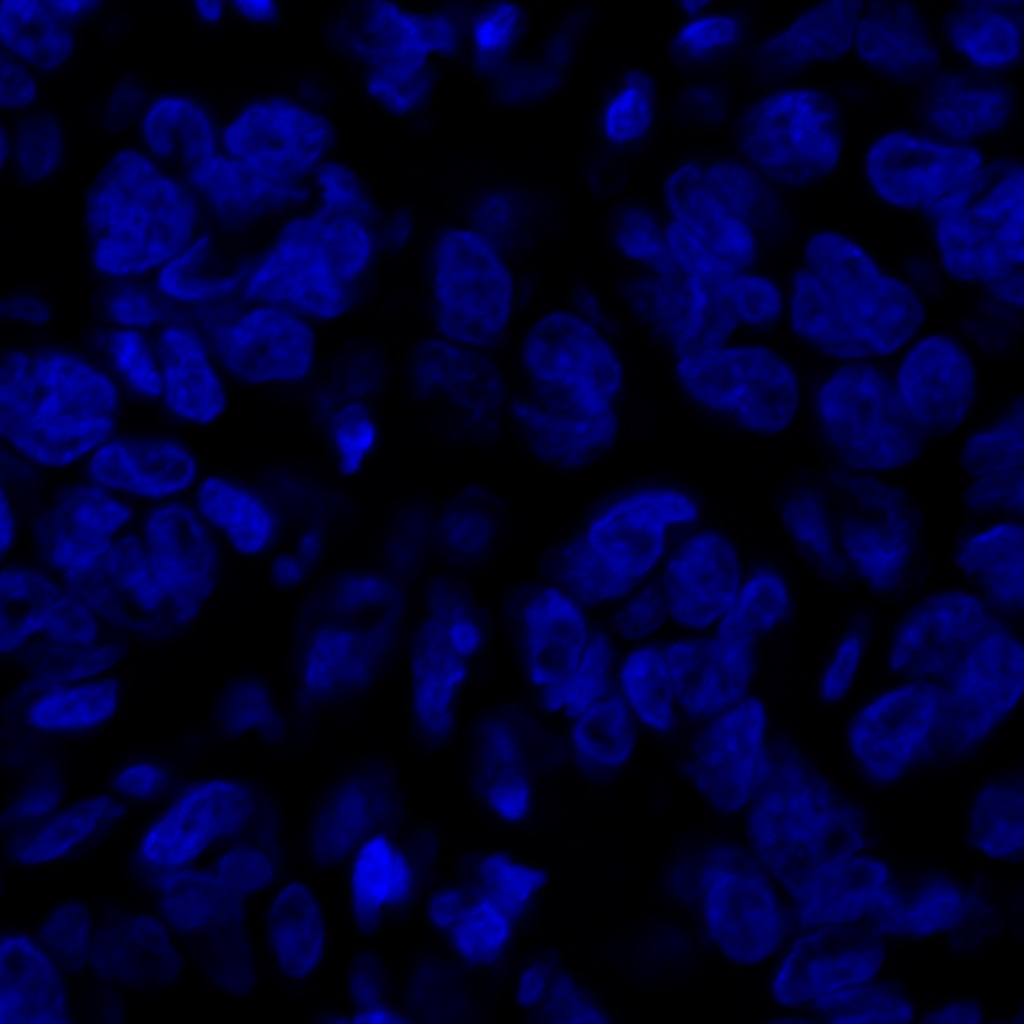

Supplement: Supplementary file 9 [file DataSheet_9.zip › ROW DATA Supplymetary Figures/Supplymentary Figure 5/Supplymentary Figure 5B/Supplymentary Figure 5B DAPI.jpg]

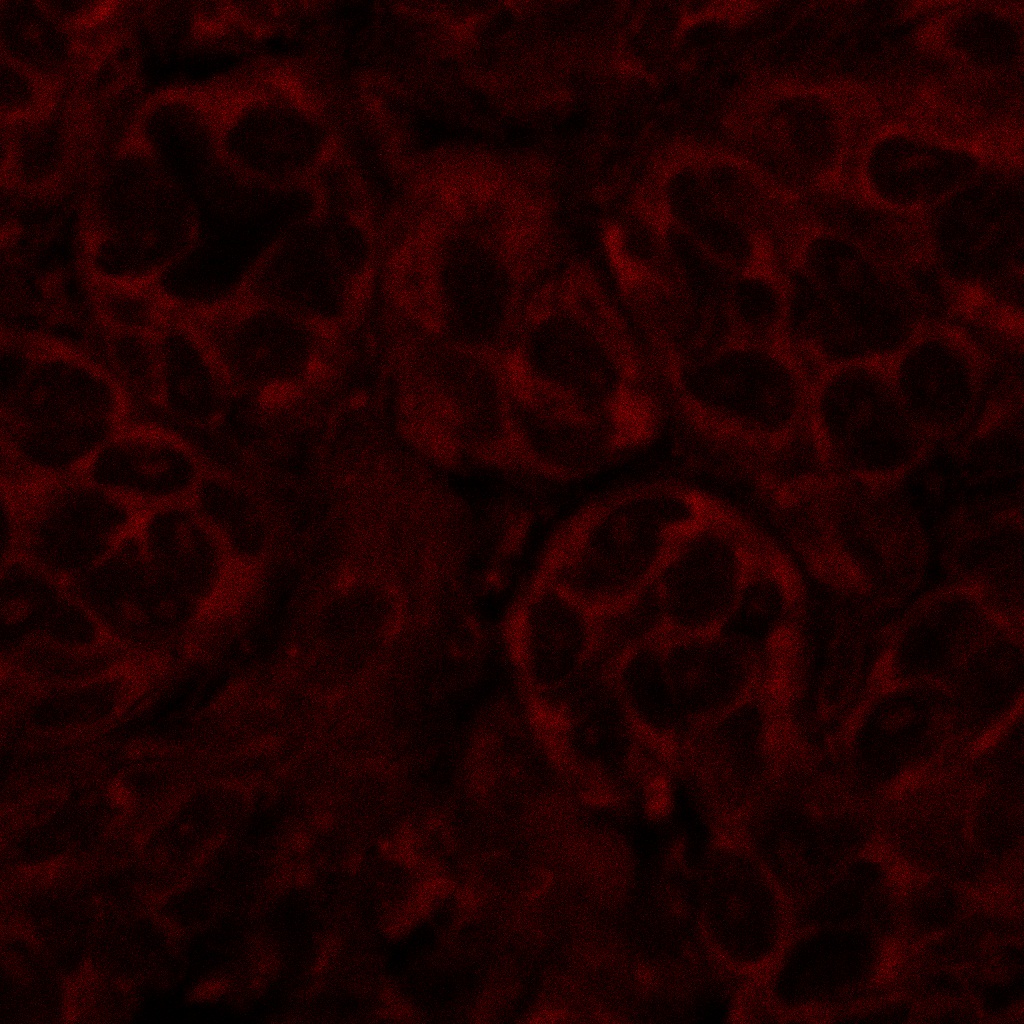

Supplement: Supplementary file 9 [file DataSheet_9.zip › ROW DATA Supplymetary Figures/Supplymentary Figure 5/Supplymentary Figure 5B/Supplymentary Figure 5B MelanA.jpg]

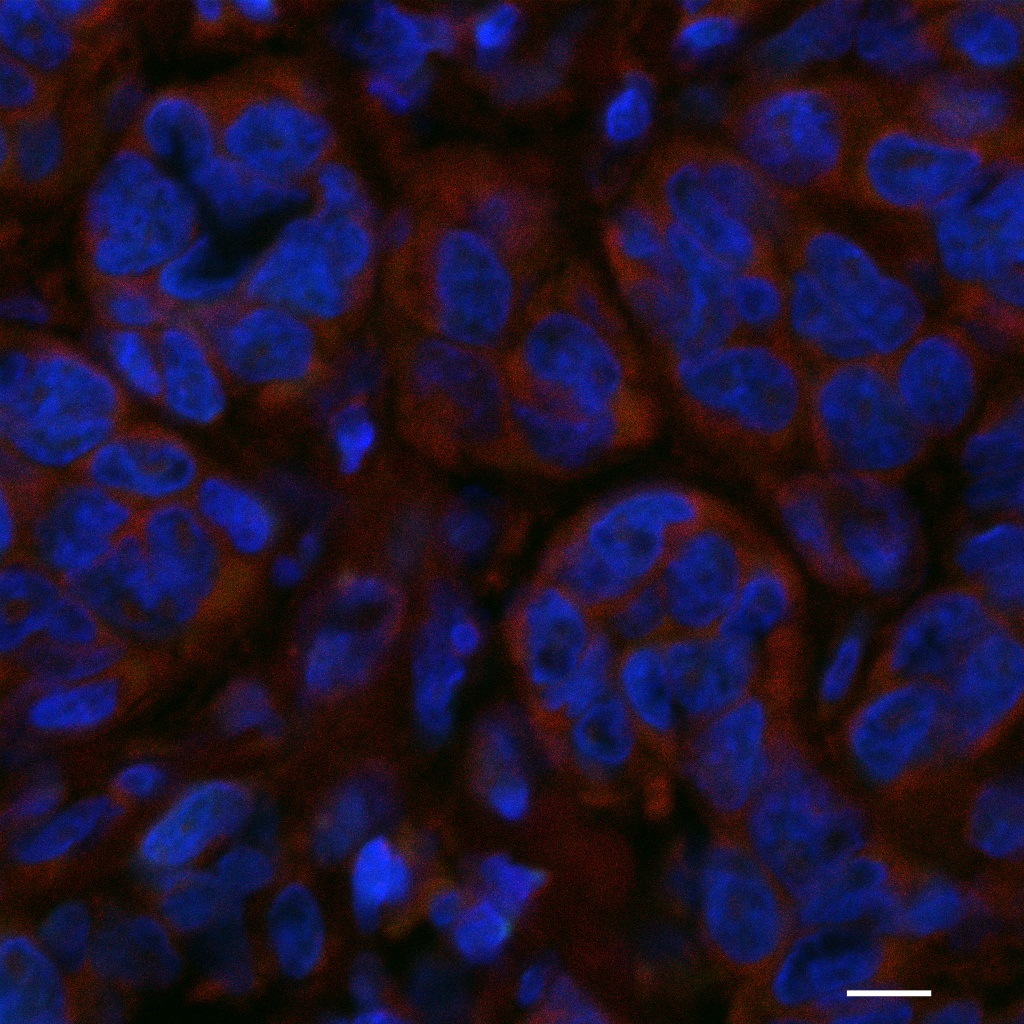

Supplement: Supplementary file 9 [file DataSheet_9.zip › ROW DATA Supplymetary Figures/Supplymentary Figure 5/Supplymentary Figure 5B/Supplymentary Figure 5B Merge.jpg]

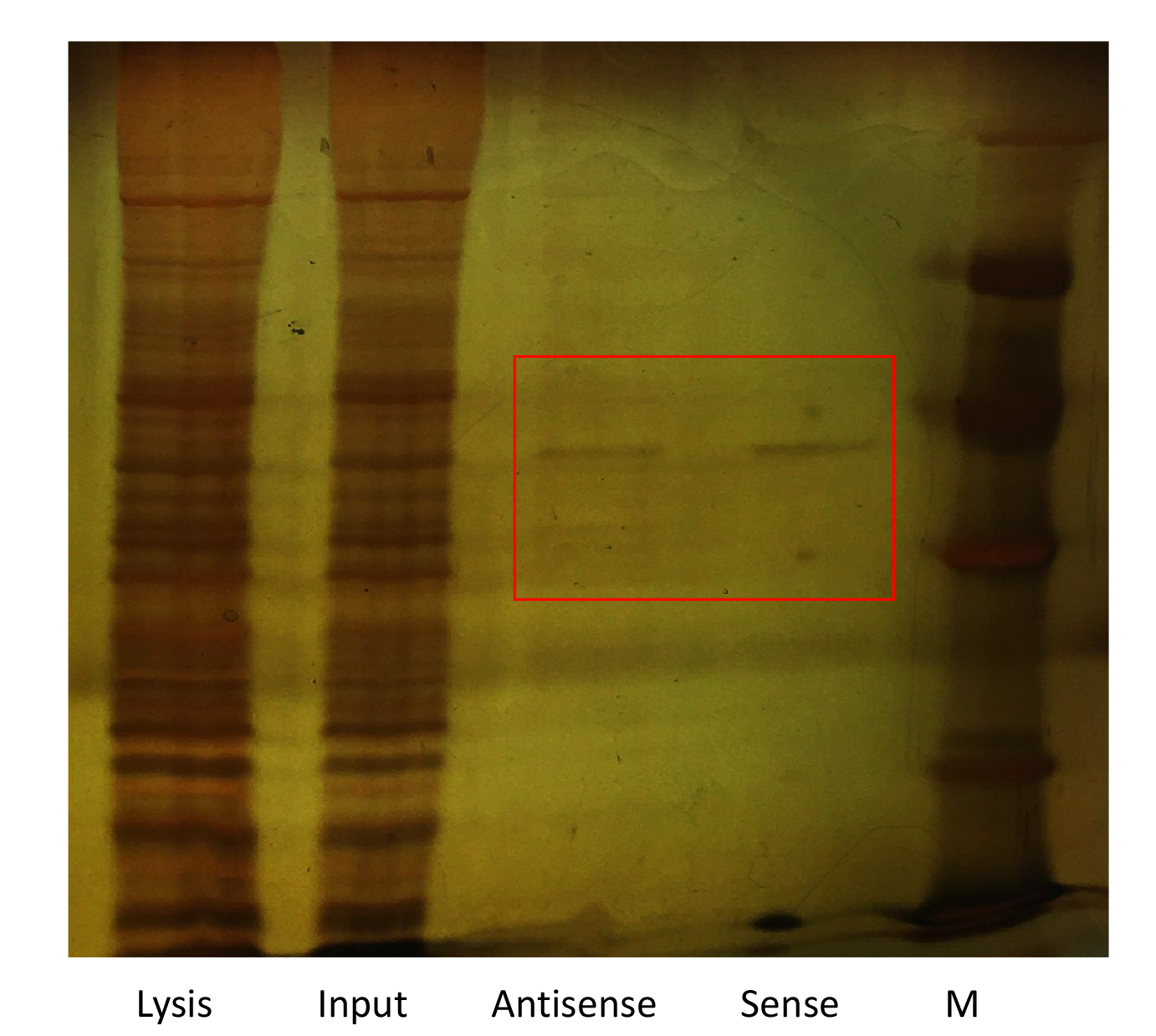

Supplement: Supplementary file 9 [file DataSheet_9.zip › ROW DATA Supplymetary Figures/Supplymentary Figure 8/Supplymentary Figure 8A.tif]

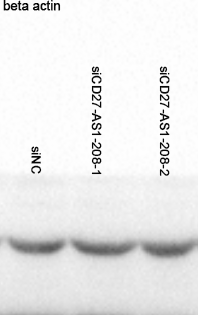

Supplement: Supplementary file 9 [file DataSheet_9.zip › ROW DATA Supplymetary Figures/Supplymentary Figure 8/Supplymentary Figure 8D/beta actin.tif]

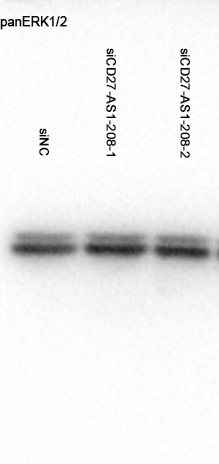

Supplement: Supplementary file 9 [file DataSheet_9.zip › ROW DATA Supplymetary Figures/Supplymentary Figure 8/Supplymentary Figure 8D/panERK 1 and 2.tif]

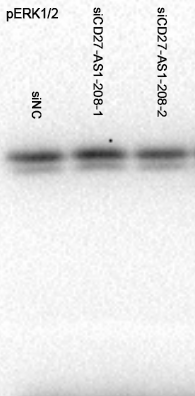

Supplement: Supplementary file 9 [file DataSheet_9.zip › ROW DATA Supplymetary Figures/Supplymentary Figure 8/Supplymentary Figure 8D/pERK 1 and 2.tif]

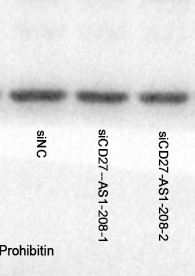

Supplement: Supplementary file 9 [file DataSheet_9.zip › ROW DATA Supplymetary Figures/Supplymentary Figure 8/Supplymentary Figure 8D/Prohibitin.tif]

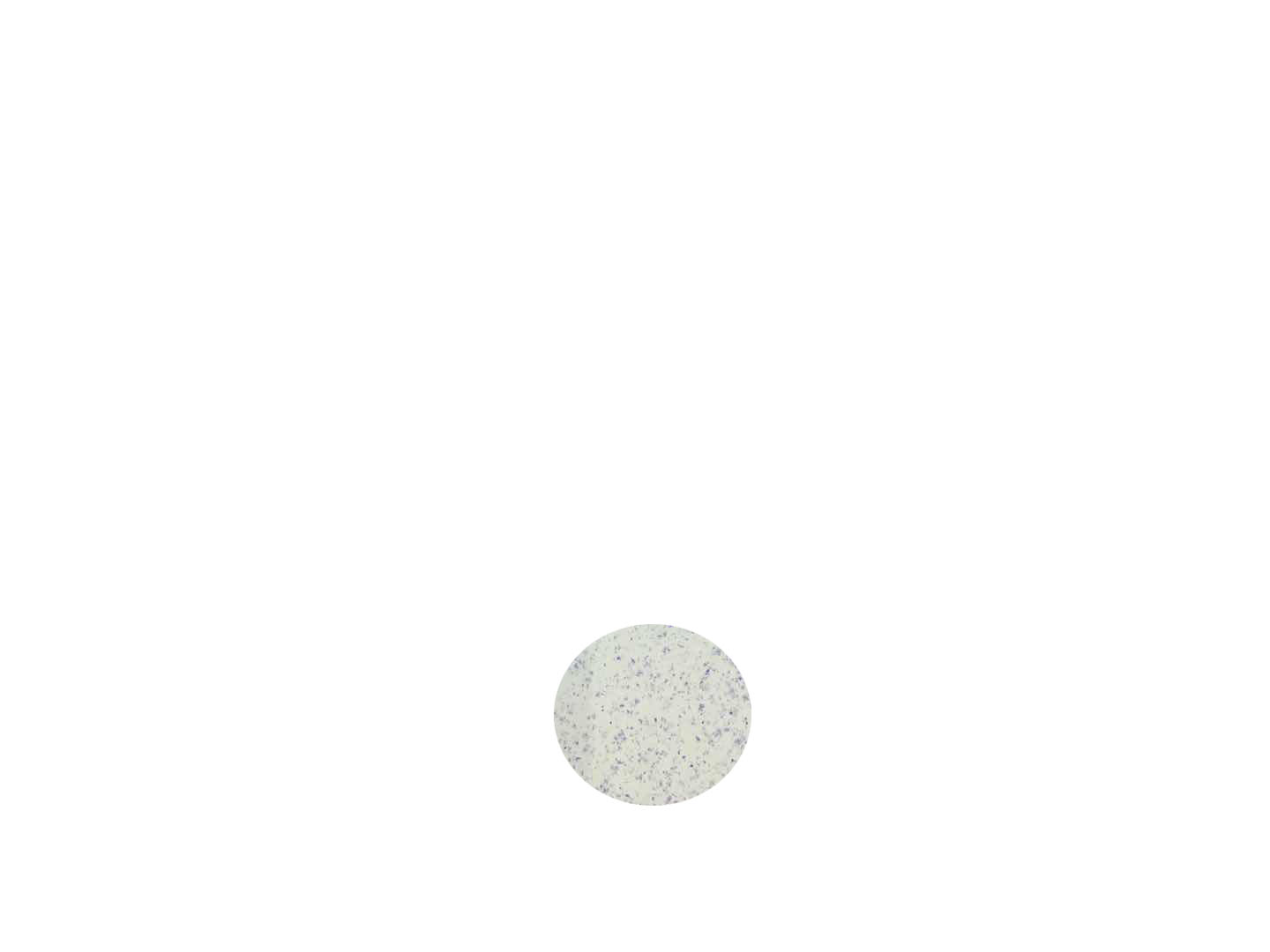

Supplement: Supplementary file 9 [file DataSheet_9.zip › ROW DATA Supplymetary Figures/Supplymentary Figure 9/Supplymentary Figure 9C/A2058-siCD27-AS1-208+OA.jpg]

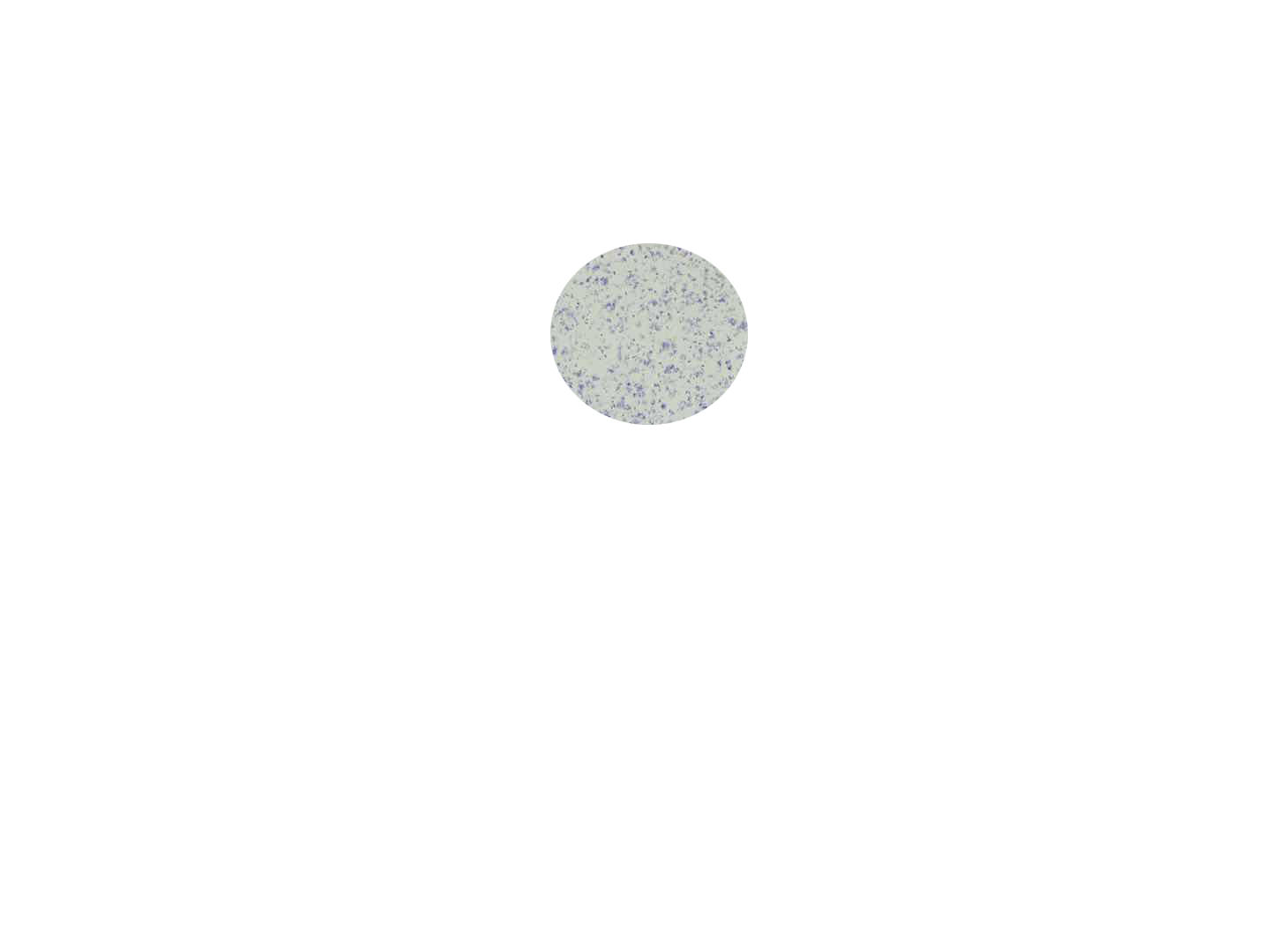

Supplement: Supplementary file 9 [file DataSheet_9.zip › ROW DATA Supplymetary Figures/Supplymentary Figure 9/Supplymentary Figure 9C/A2058-siCD27-AS1-208.jpg]

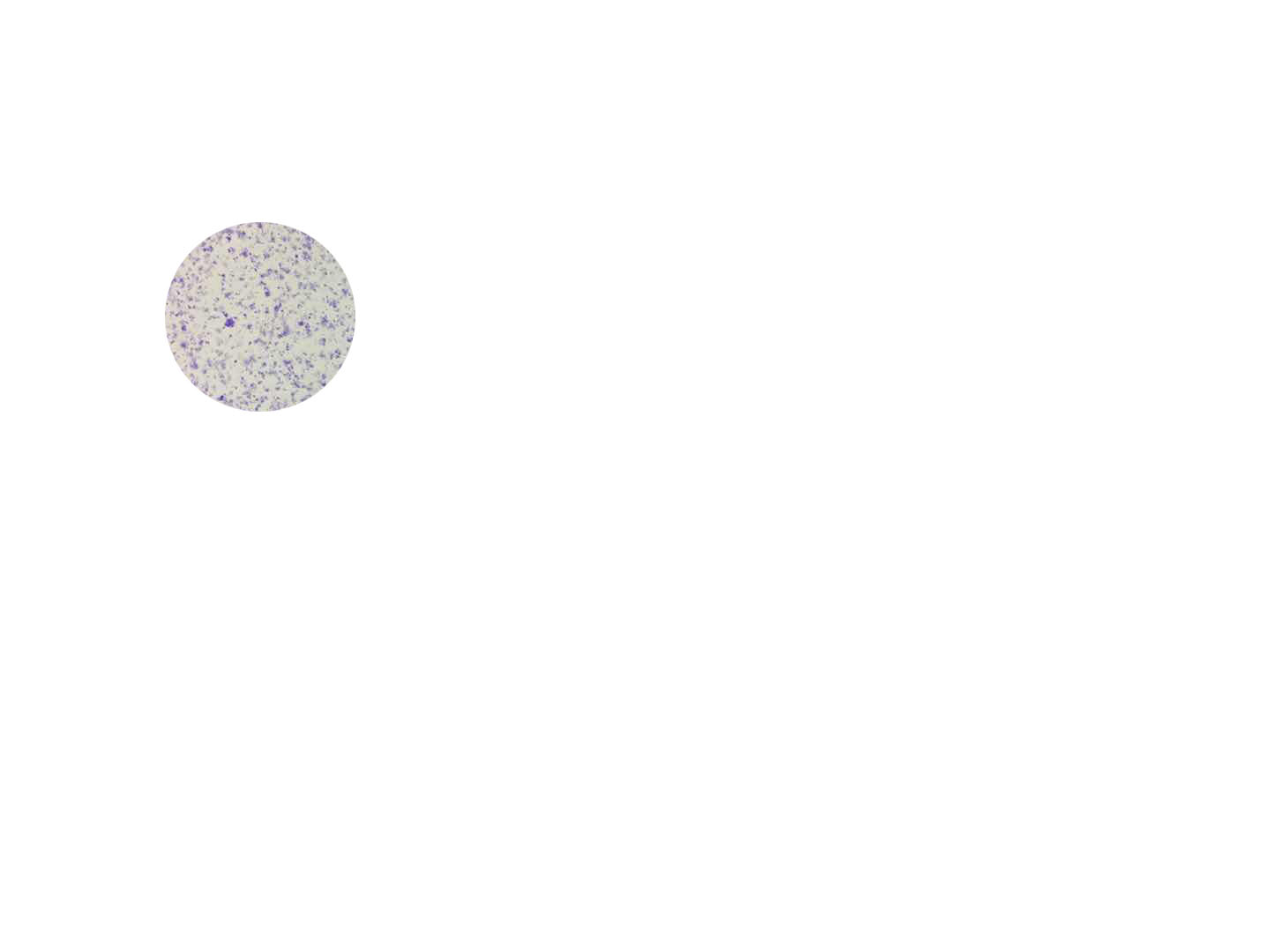

Supplement: Supplementary file 9 [file DataSheet_9.zip › ROW DATA Supplymetary Figures/Supplymentary Figure 9/Supplymentary Figure 9C/A2058-siNC.jpg]

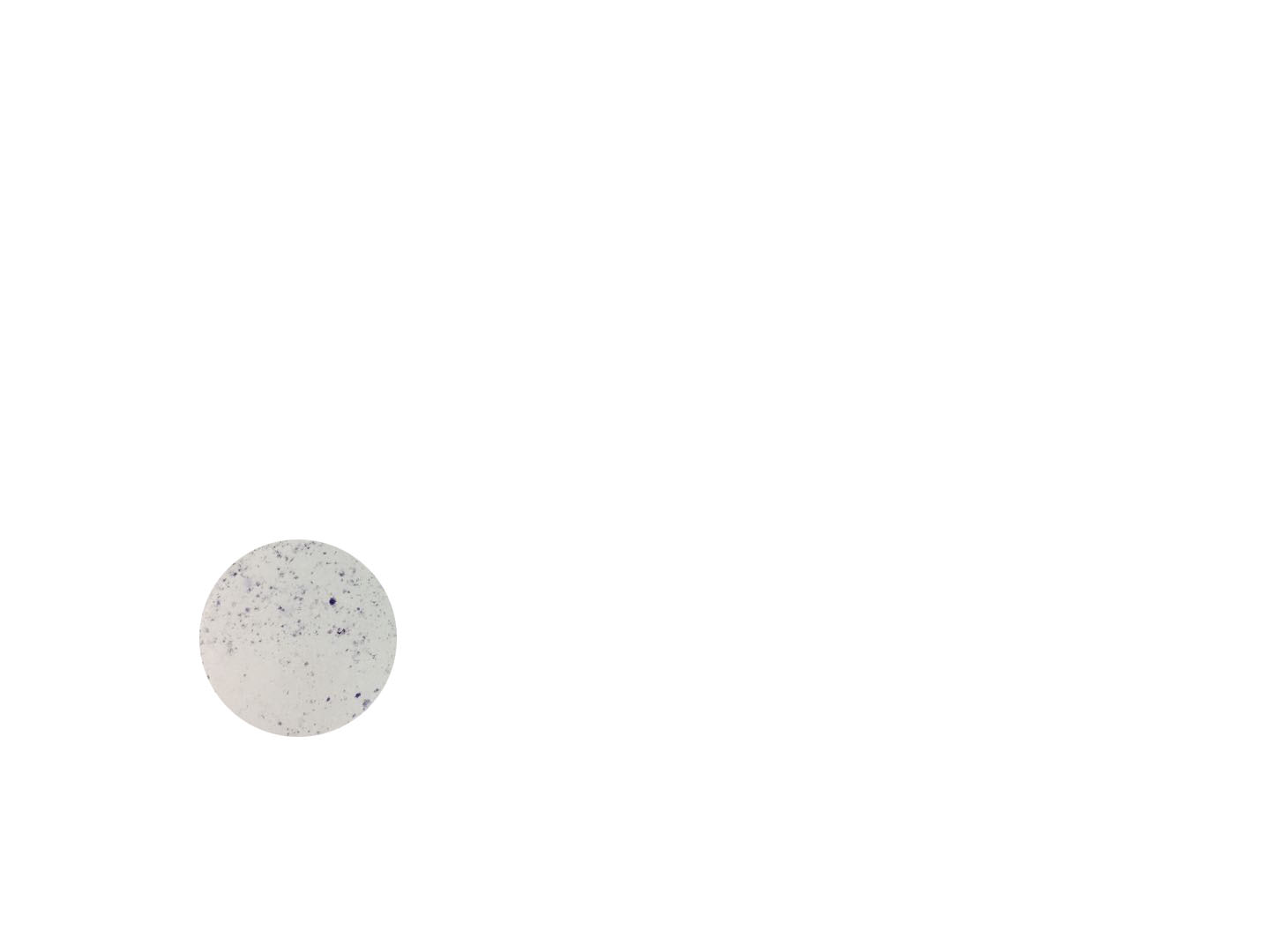

Supplement: Supplementary file 9 [file DataSheet_9.zip › ROW DATA Supplymetary Figures/Supplymentary Figure 9/Supplymentary Figure 9C/A375 siCD27-AS1-208+OA.jpg]

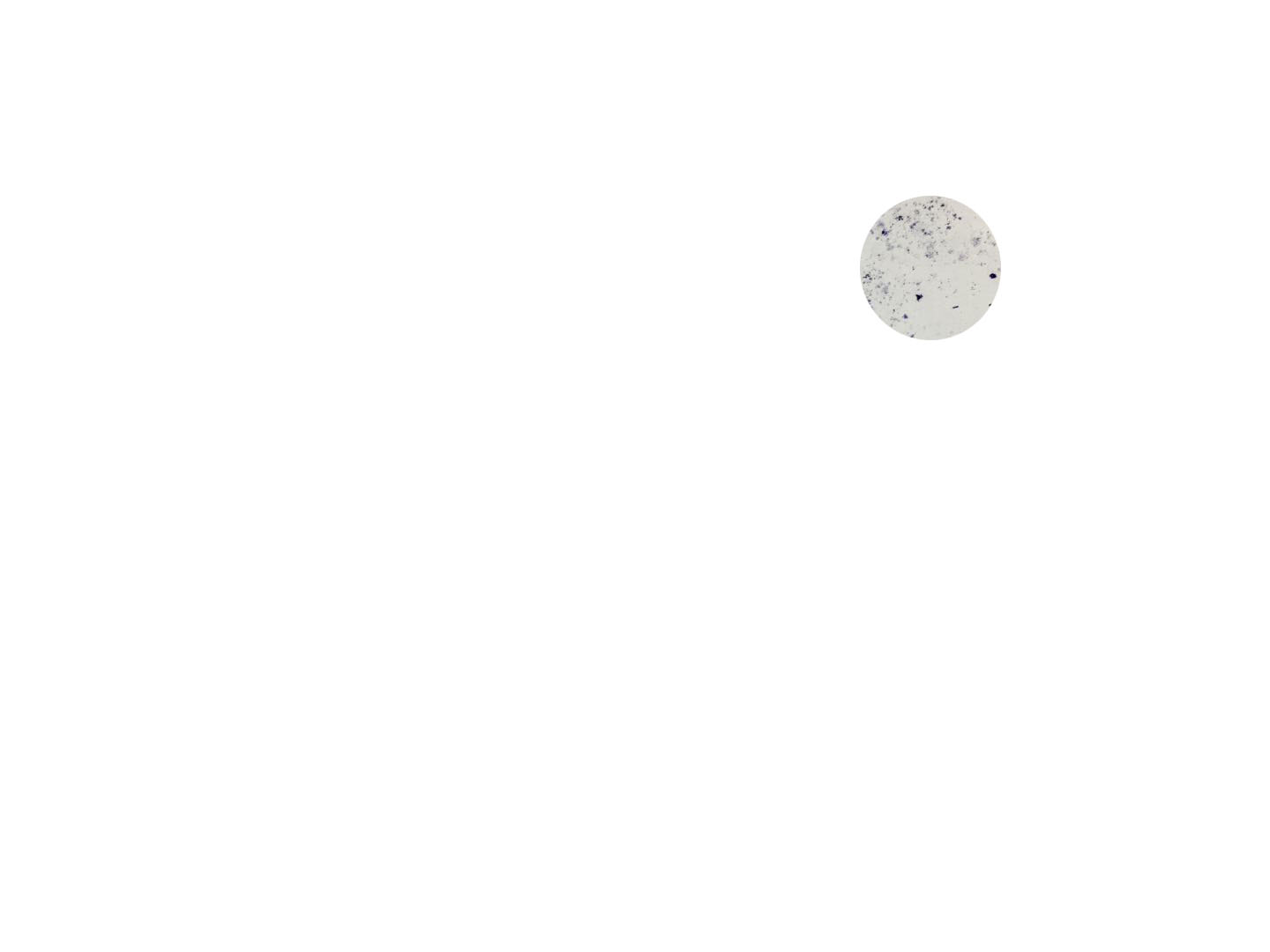

Supplement: Supplementary file 9 [file DataSheet_9.zip › ROW DATA Supplymetary Figures/Supplymentary Figure 9/Supplymentary Figure 9C/A375 siCD27-AS1-208.jpg]

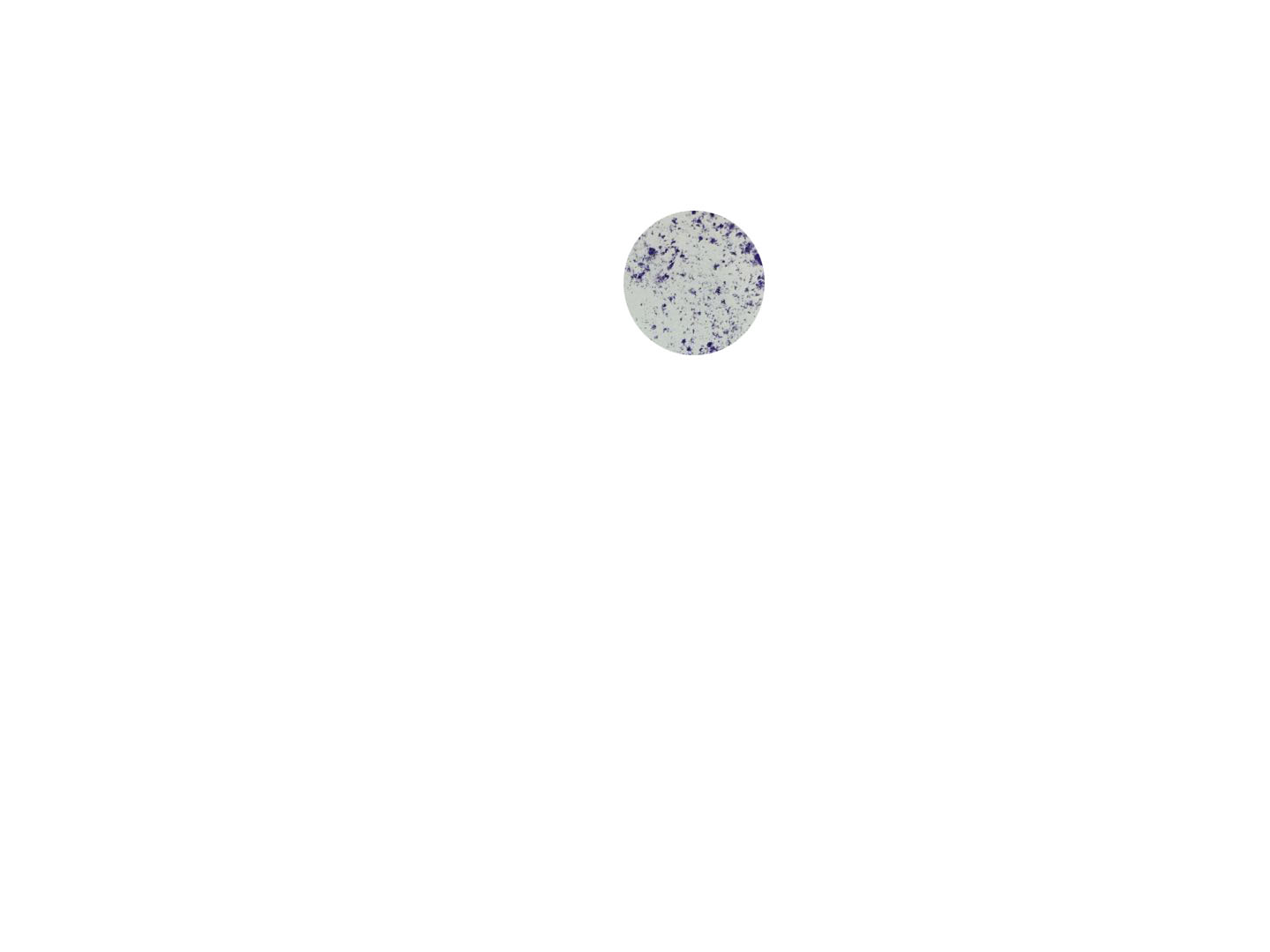

Supplement: Supplementary file 9 [file DataSheet_9.zip › ROW DATA Supplymetary Figures/Supplymentary Figure 9/Supplymentary Figure 9C/A375 siNC.jpg]

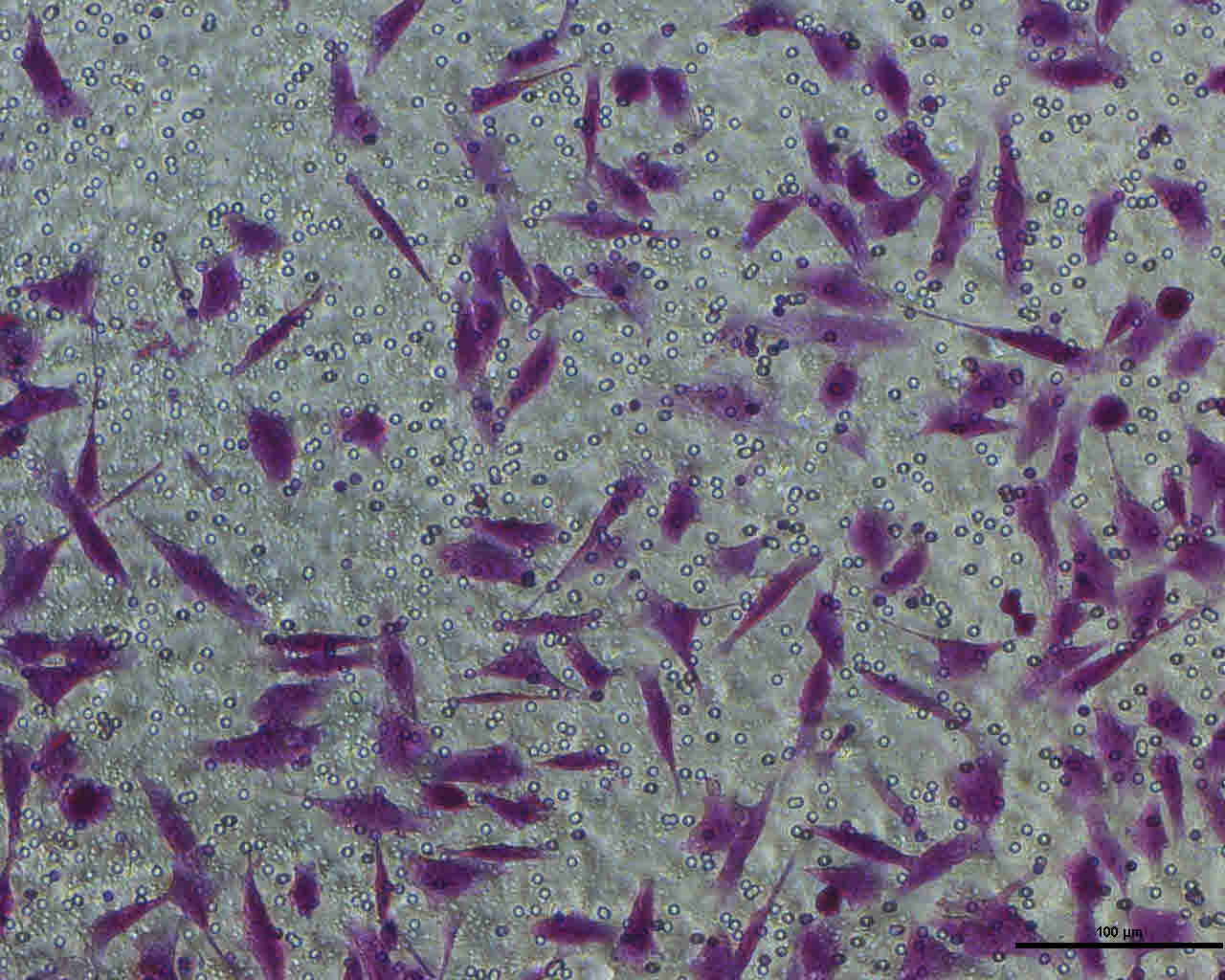

Supplement: Supplementary file 9 [file DataSheet_9.zip › ROW DATA Supplymetary Figures/Supplymentary Figure 9/Supplymentary Figure 9D/A2058 siCD27-AS1-208+OA.jpg]

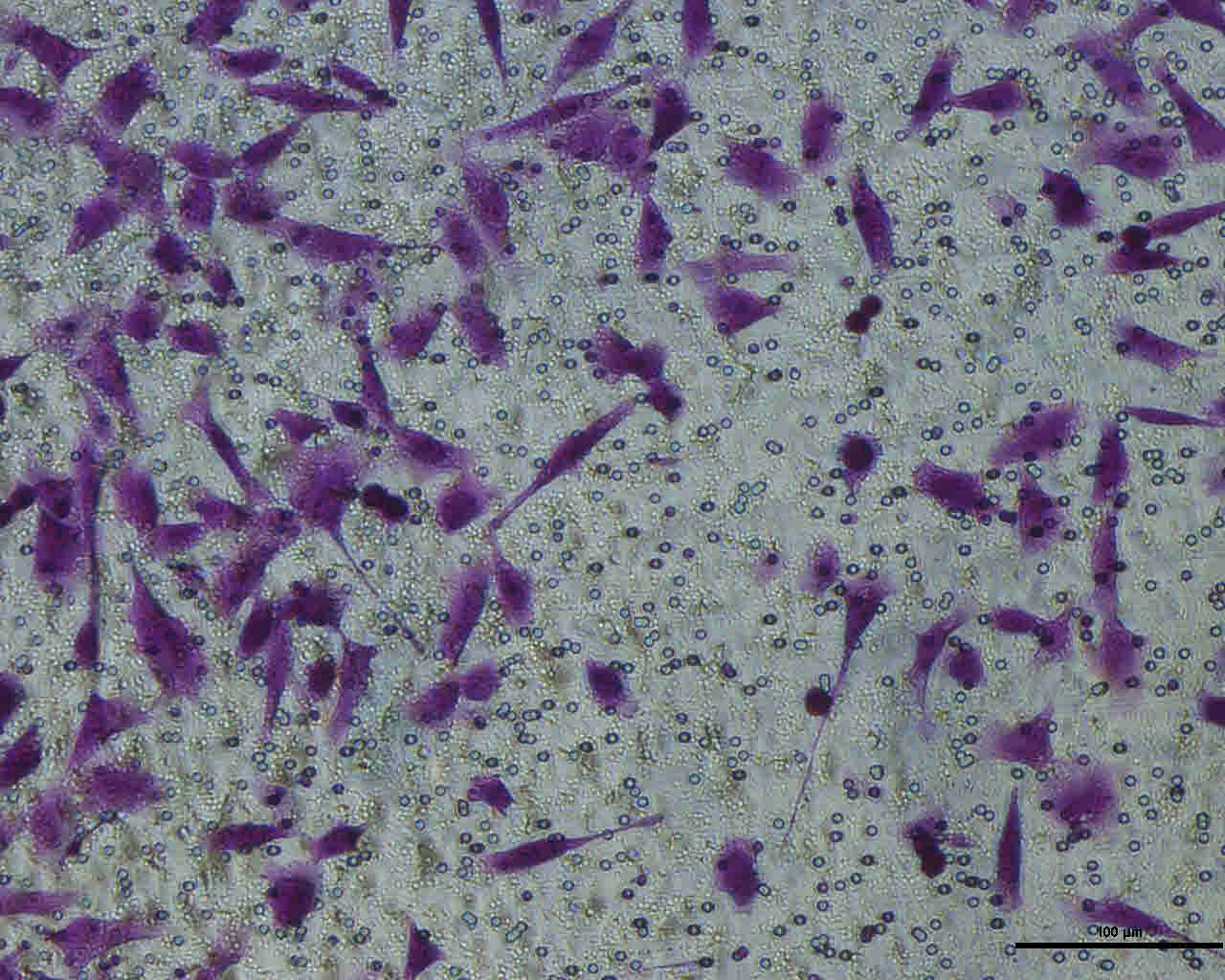

Supplement: Supplementary file 9 [file DataSheet_9.zip › ROW DATA Supplymetary Figures/Supplymentary Figure 9/Supplymentary Figure 9D/A2058 siCD27-AS1-208.jpg]

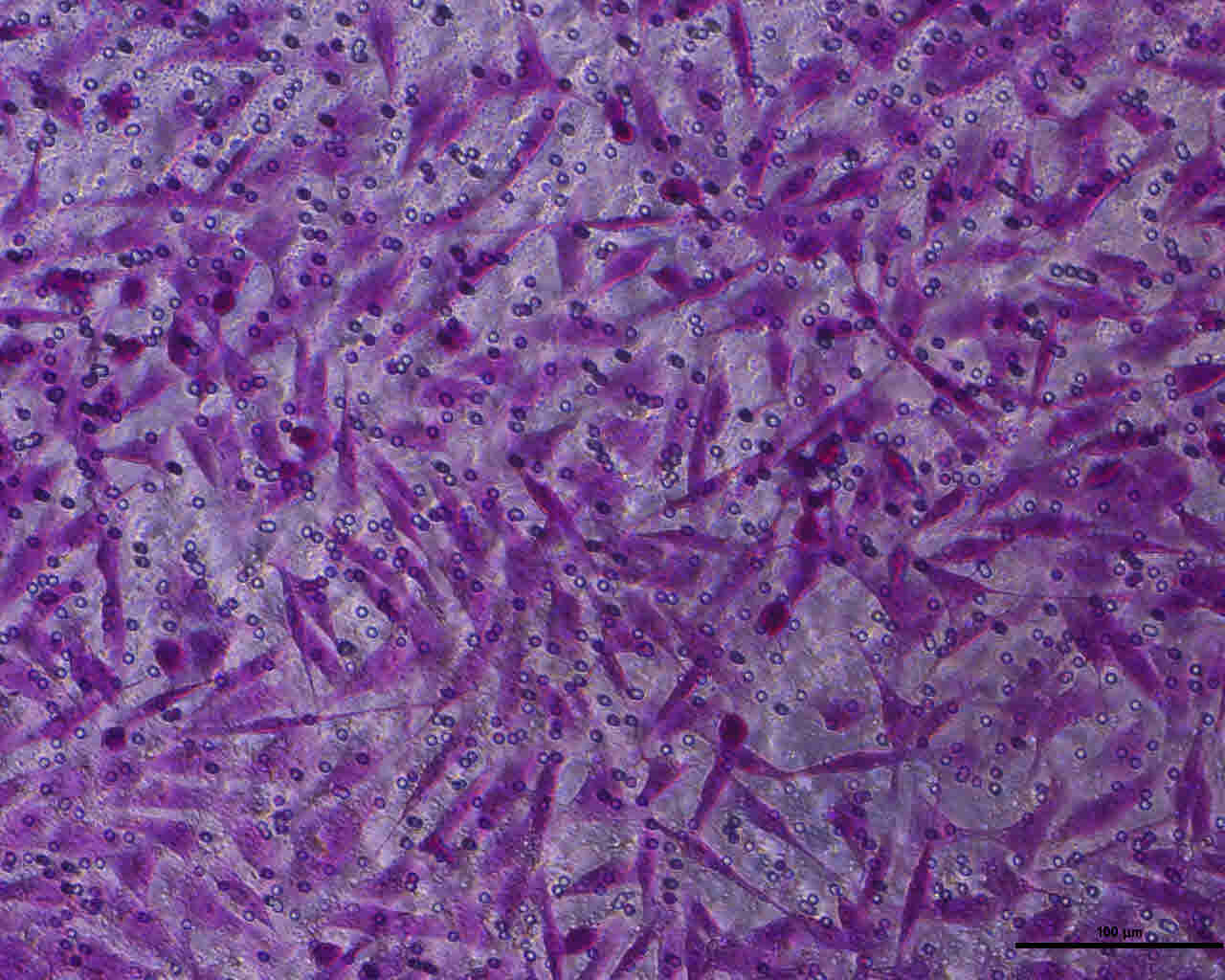

Supplement: Supplementary file 9 [file DataSheet_9.zip › ROW DATA Supplymetary Figures/Supplymentary Figure 9/Supplymentary Figure 9D/A2058 siNC.jpg]

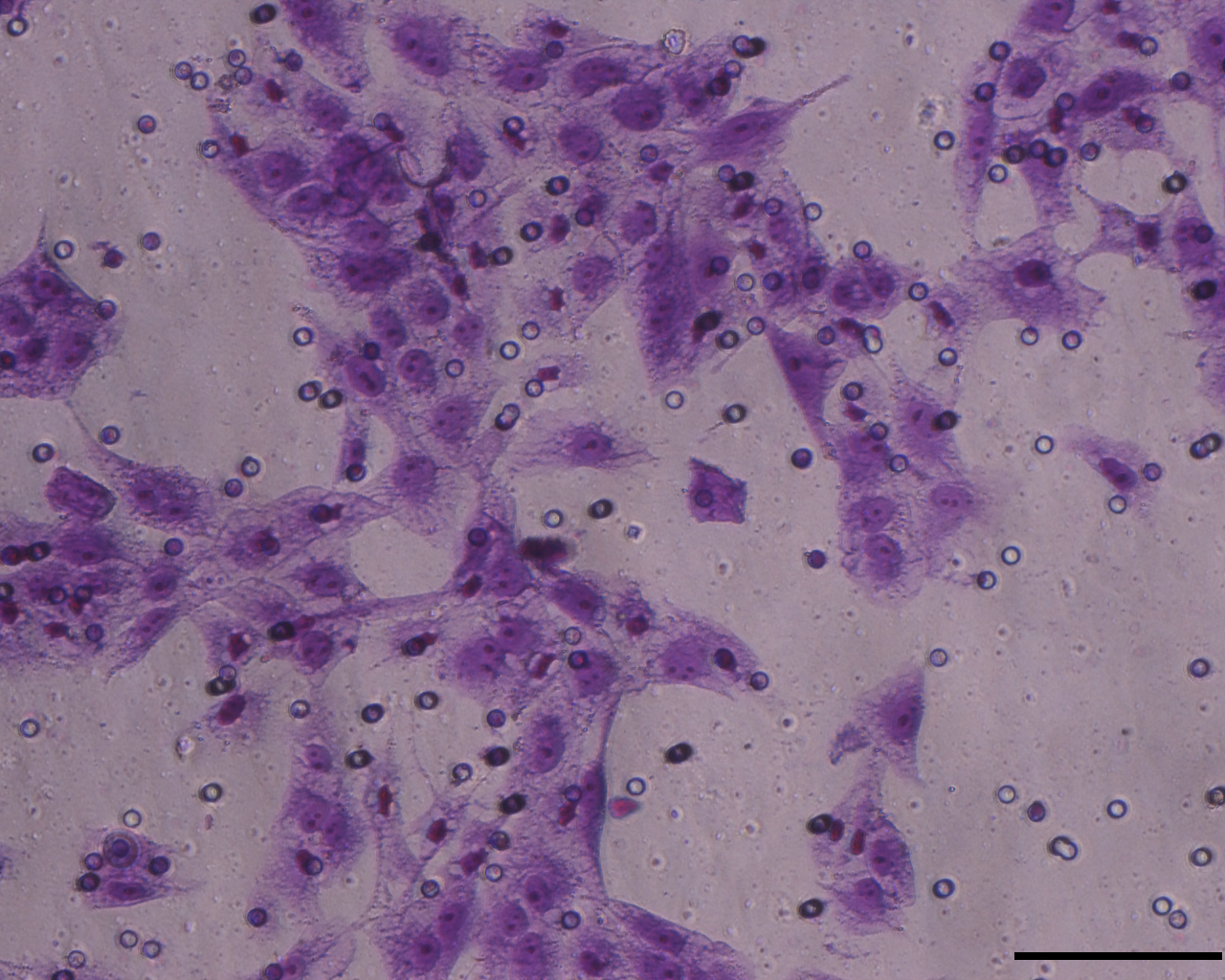

Supplement: Supplementary file 9 [file DataSheet_9.zip › ROW DATA Supplymetary Figures/Supplymentary Figure 9/Supplymentary Figure 9D/A375 siCD27-AS1-208+OA.tif]

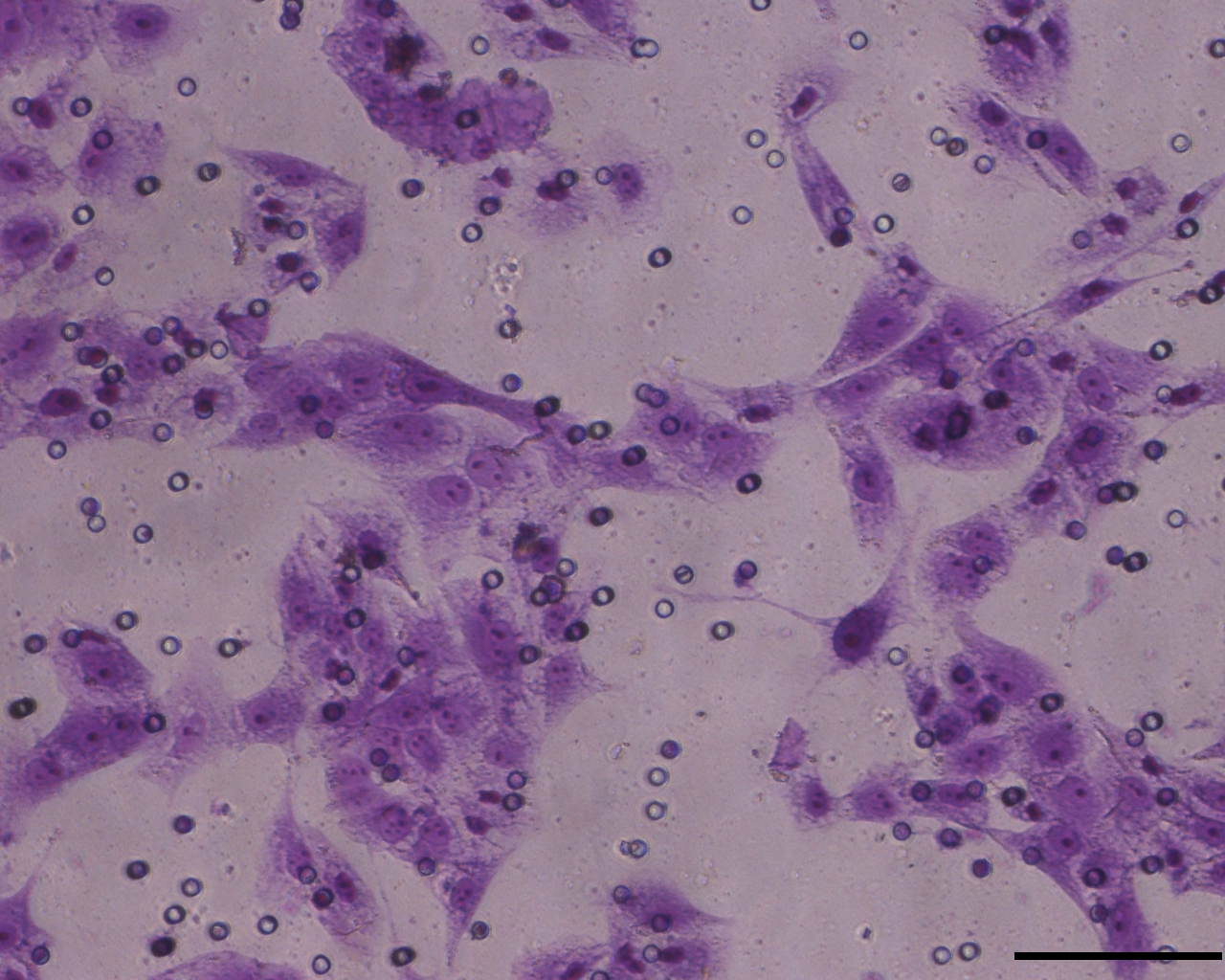

Supplement: Supplementary file 9 [file DataSheet_9.zip › ROW DATA Supplymetary Figures/Supplymentary Figure 9/Supplymentary Figure 9D/A375 siCD27-AS1-208.tif]

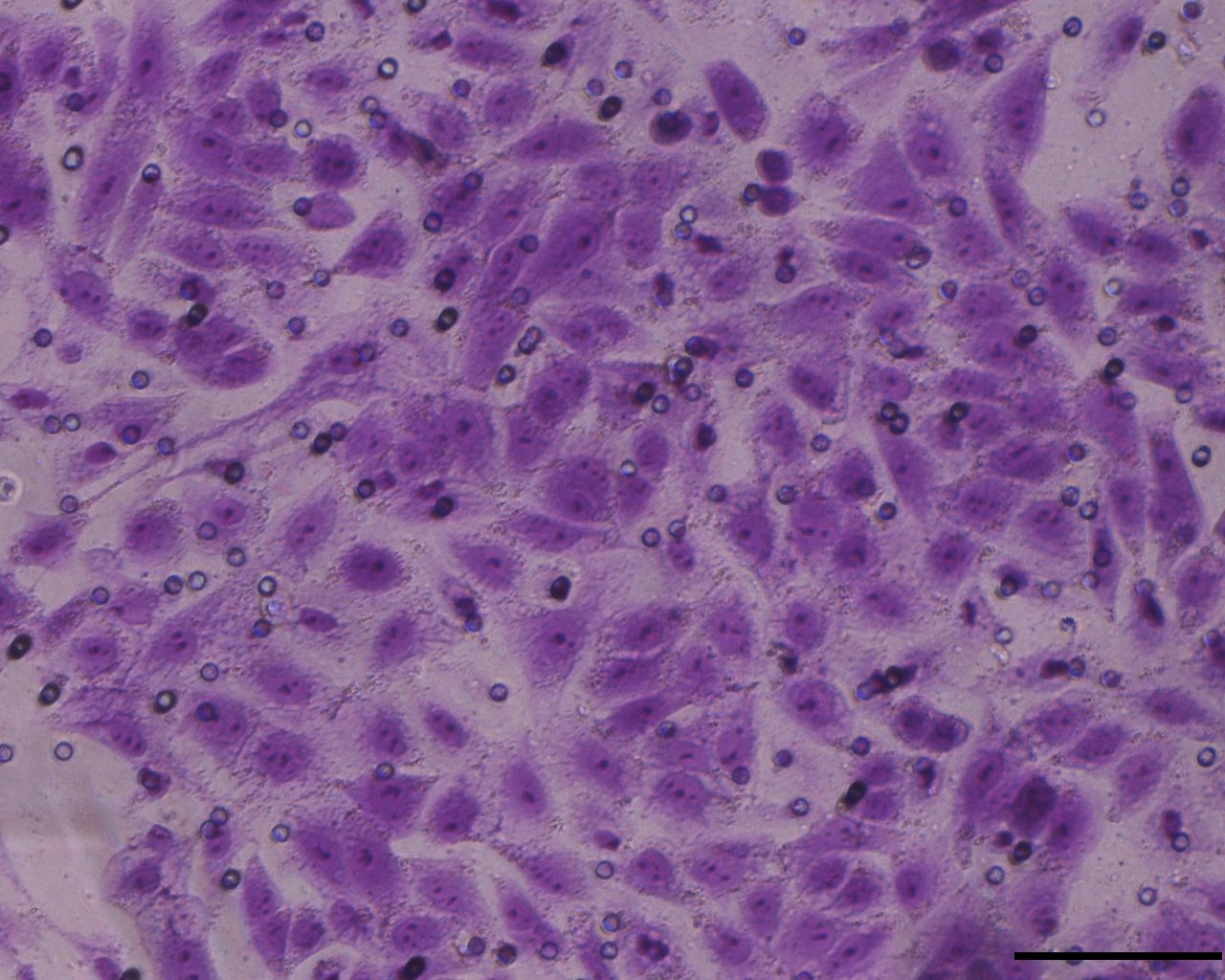

Supplement: Supplementary file 9 [file DataSheet_9.zip › ROW DATA Supplymetary Figures/Supplymentary Figure 9/Supplymentary Figure 9D/A375 siNC.tif]

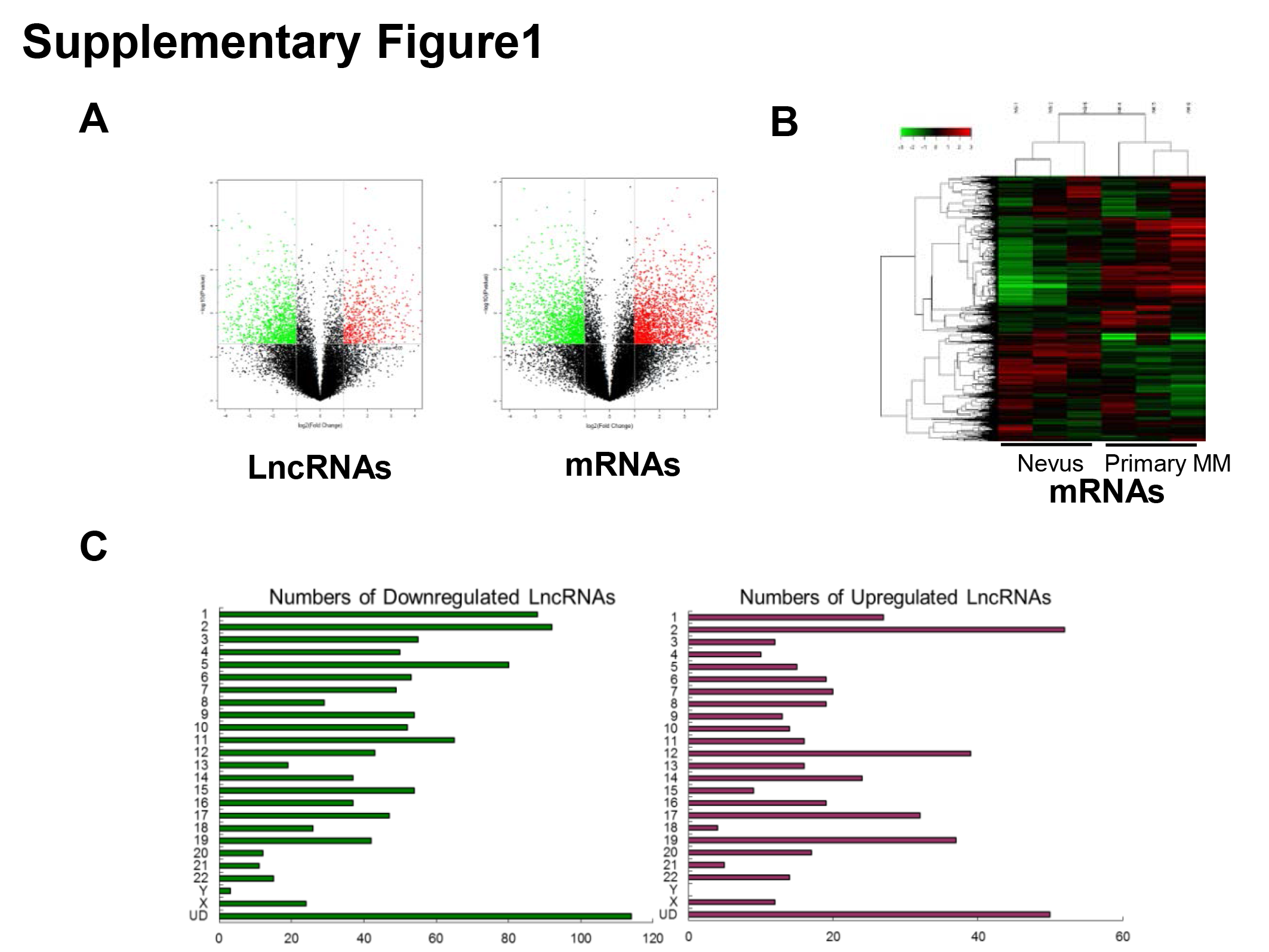

Supplement: Supplementary file 10 [file Image_1.tif]
