# Supplementary figures and images for: Long Non-Coding RNA CD27-AS1-208 Facilitates Melanoma Progression by Activating STAT3 Pathway (part 2 of 2)
Source: Front Oncol. 2022 Jan 13;11:818178. doi: 10.3389/fonc.2021.818178 (PMC8791859; doi:10.3389/fonc.2021.818178)

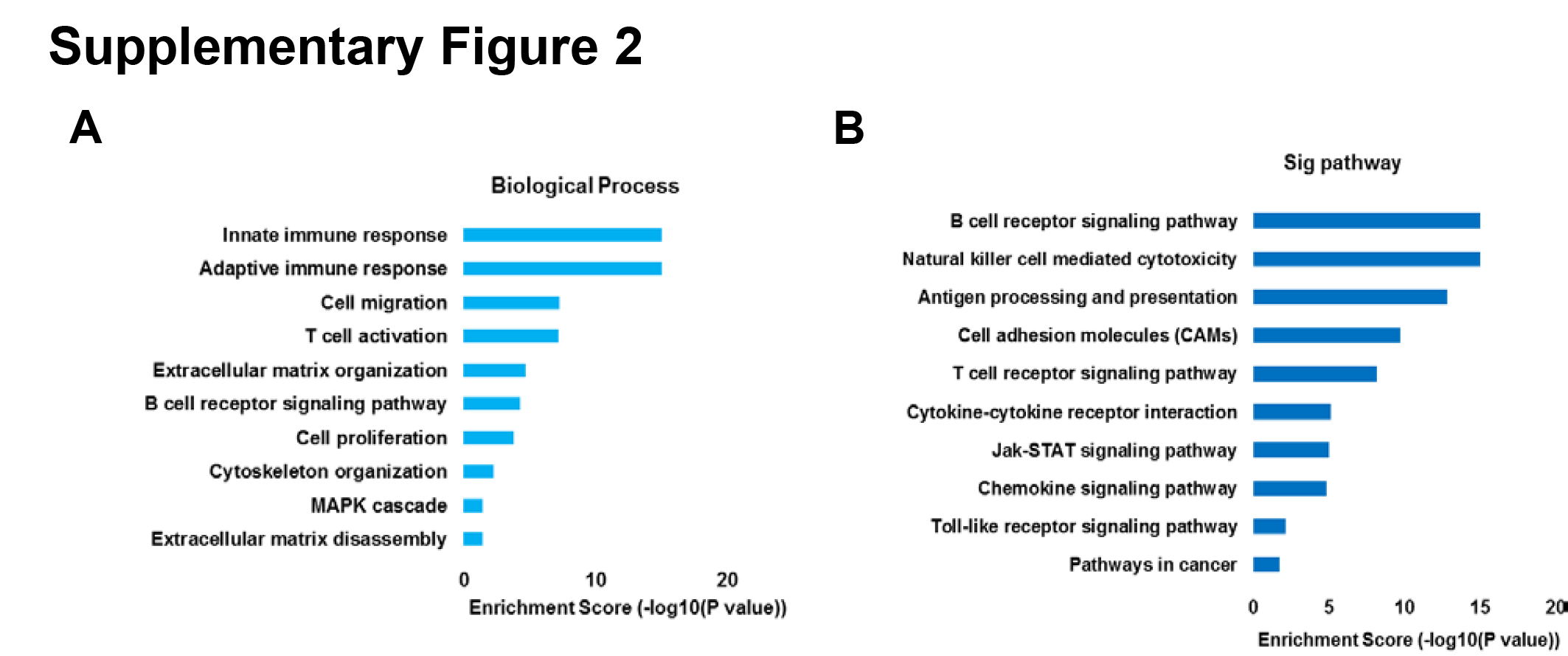

Supplement: Supplementary file 11 [file Image_2.tif]

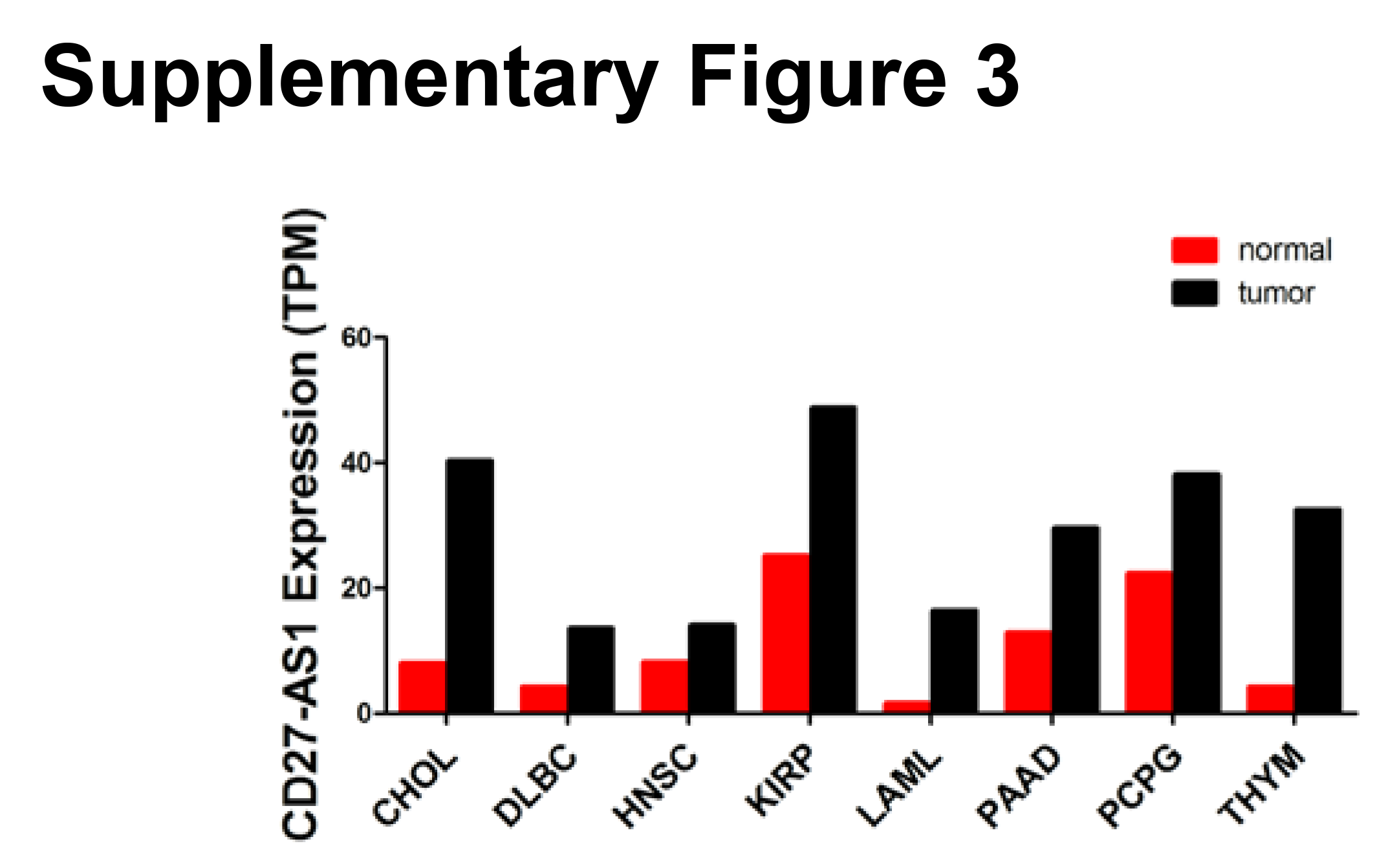

Supplement: Supplementary file 12 [file Image_3.tif]

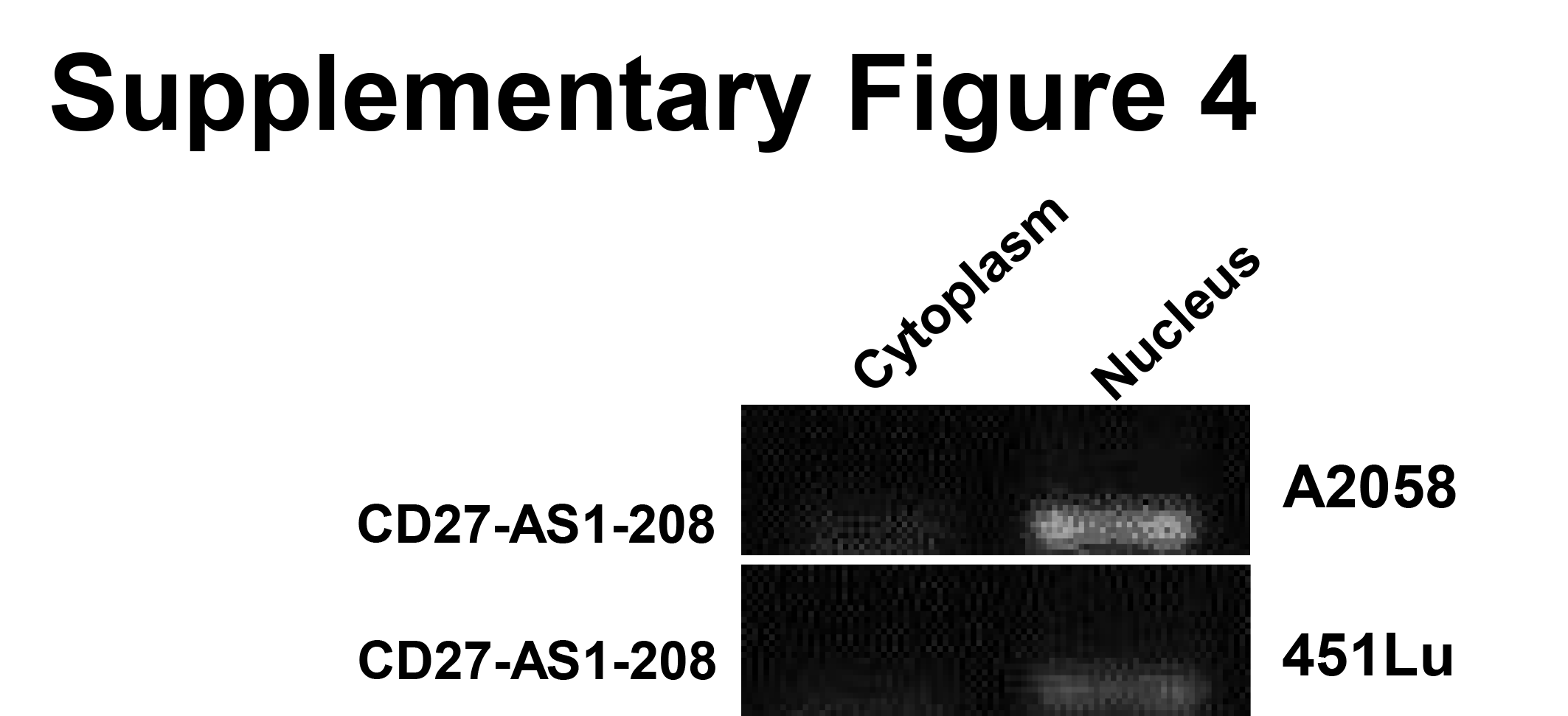

Supplement: Supplementary file 13 [file Image_4.tif]

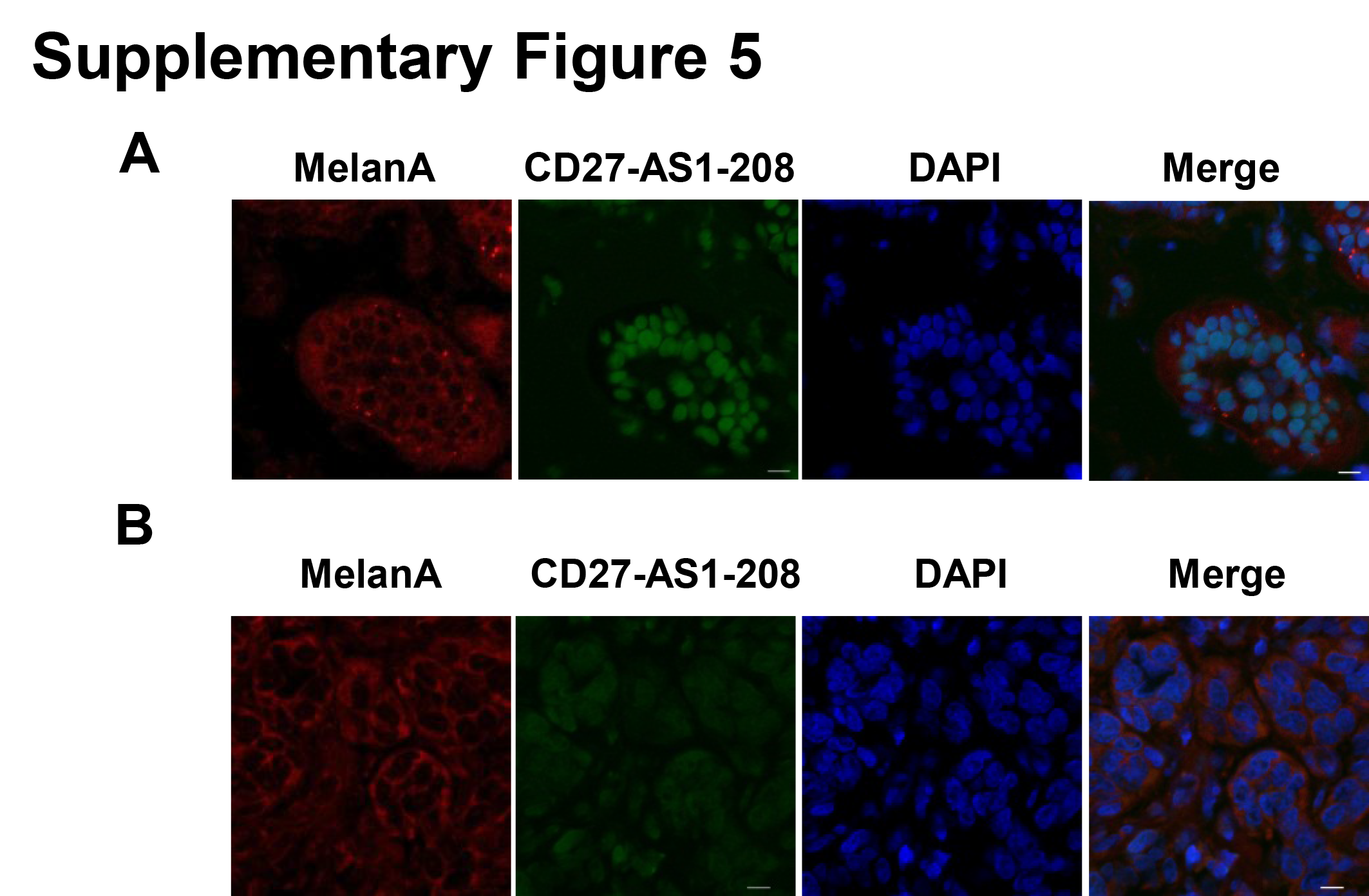

Supplement: Supplementary file 14 [file Image_5.tif]

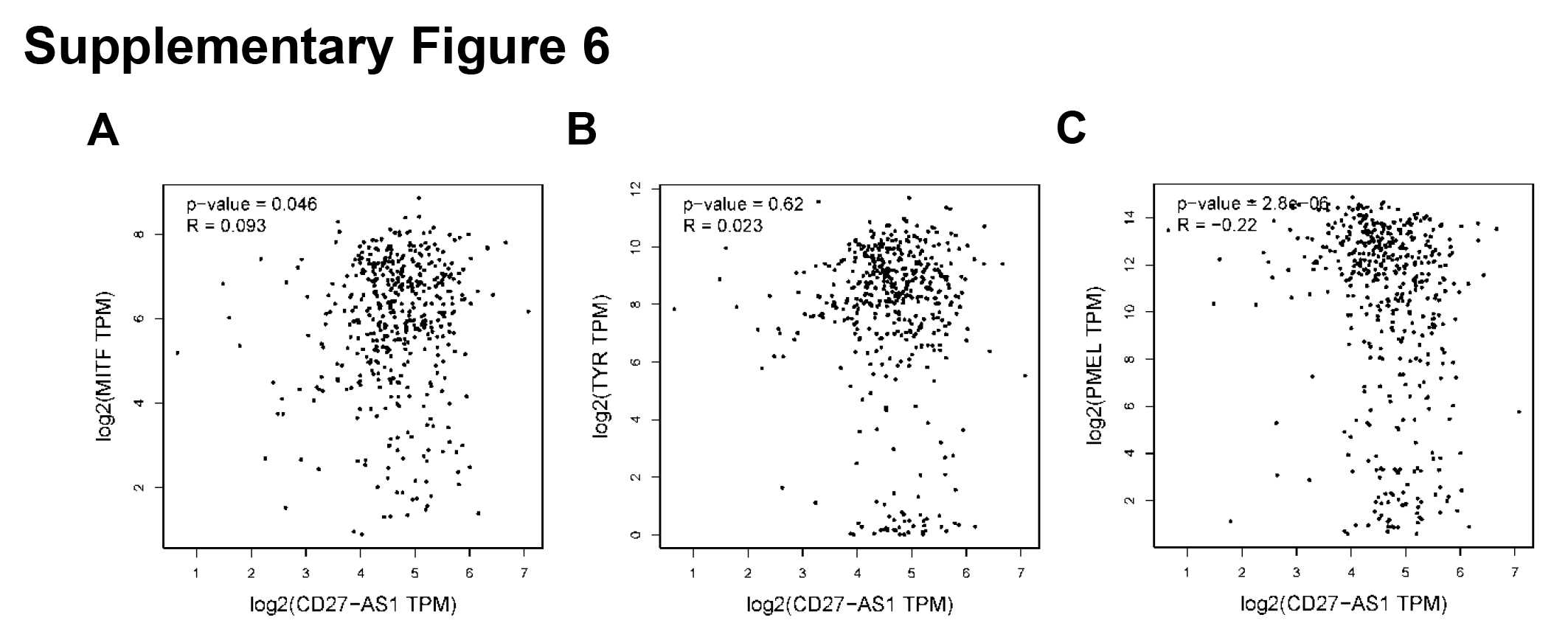

Supplement: Supplementary file 15 [file Image_6.tif]

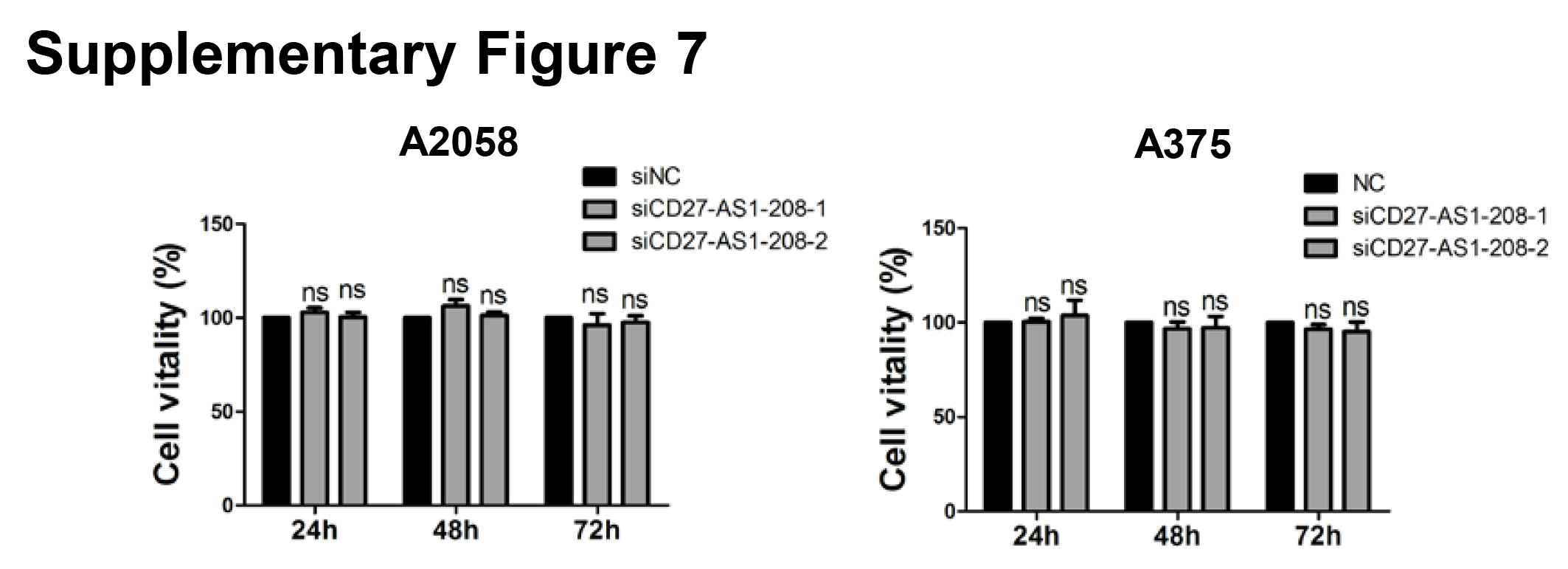

Supplement: Supplementary file 16 [file Image_7.tif]

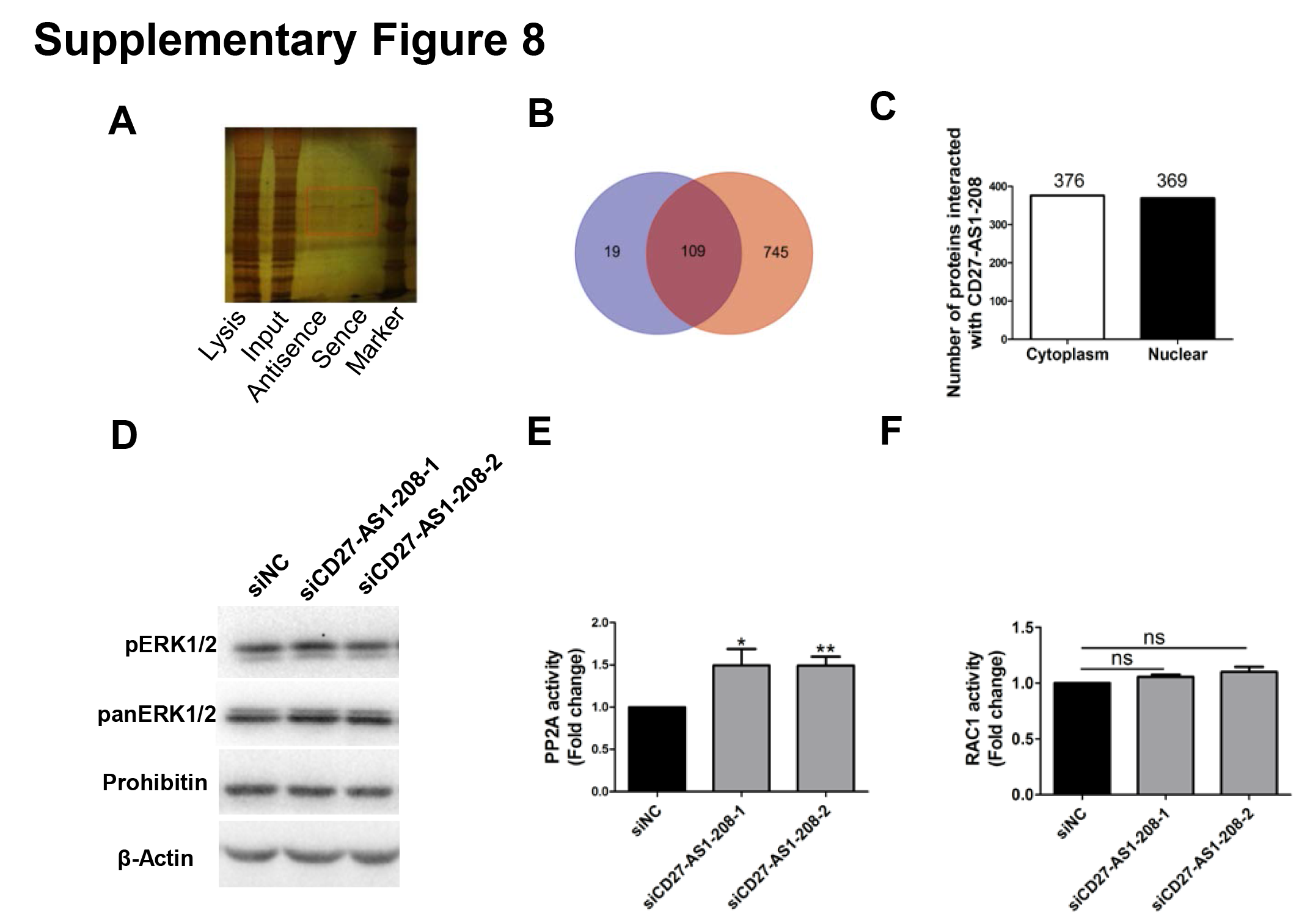

Supplement: Supplementary file 17 [file Image_8.tif]

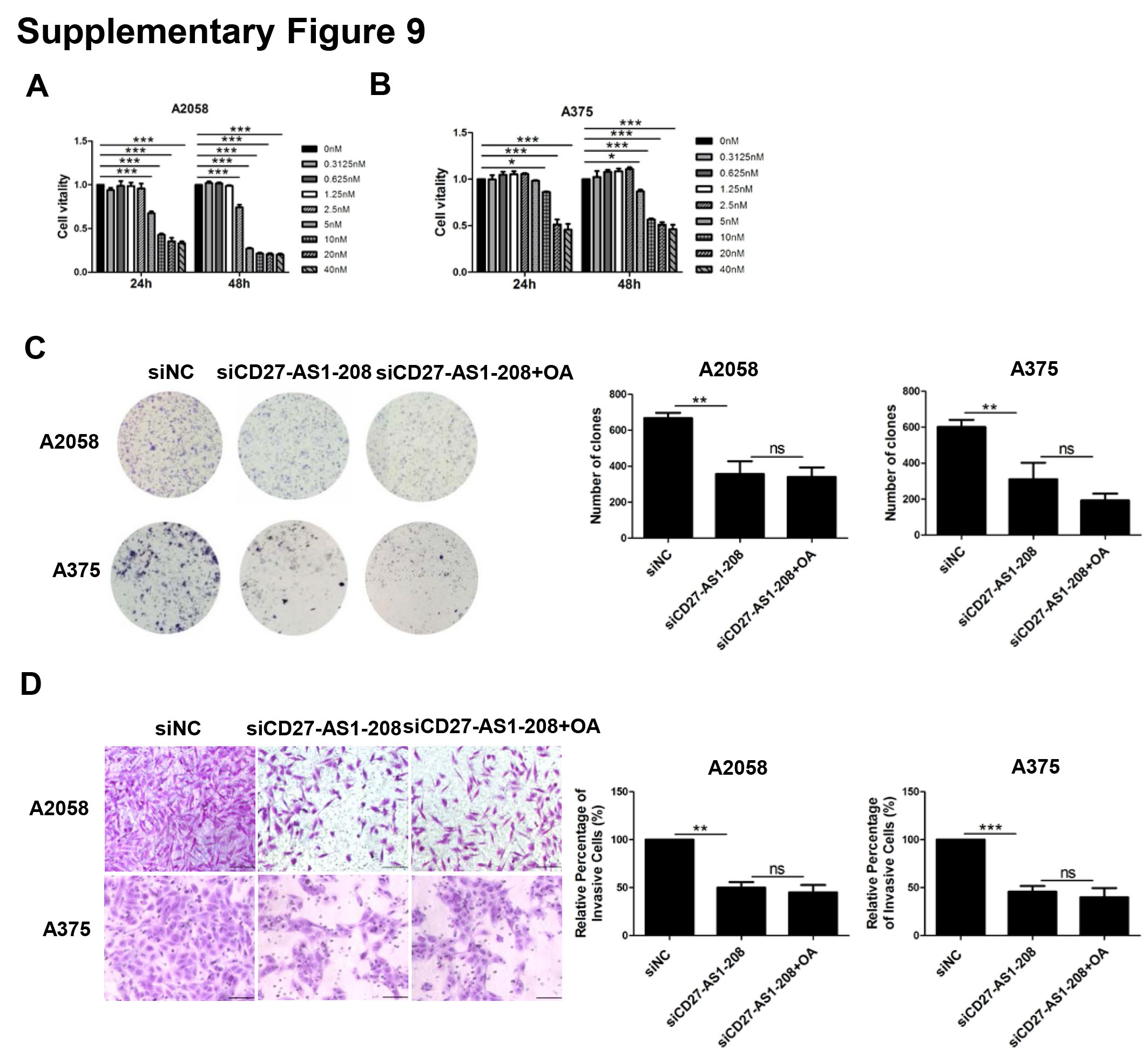

Supplement: Supplementary file 18 [file Image_9.jpg]

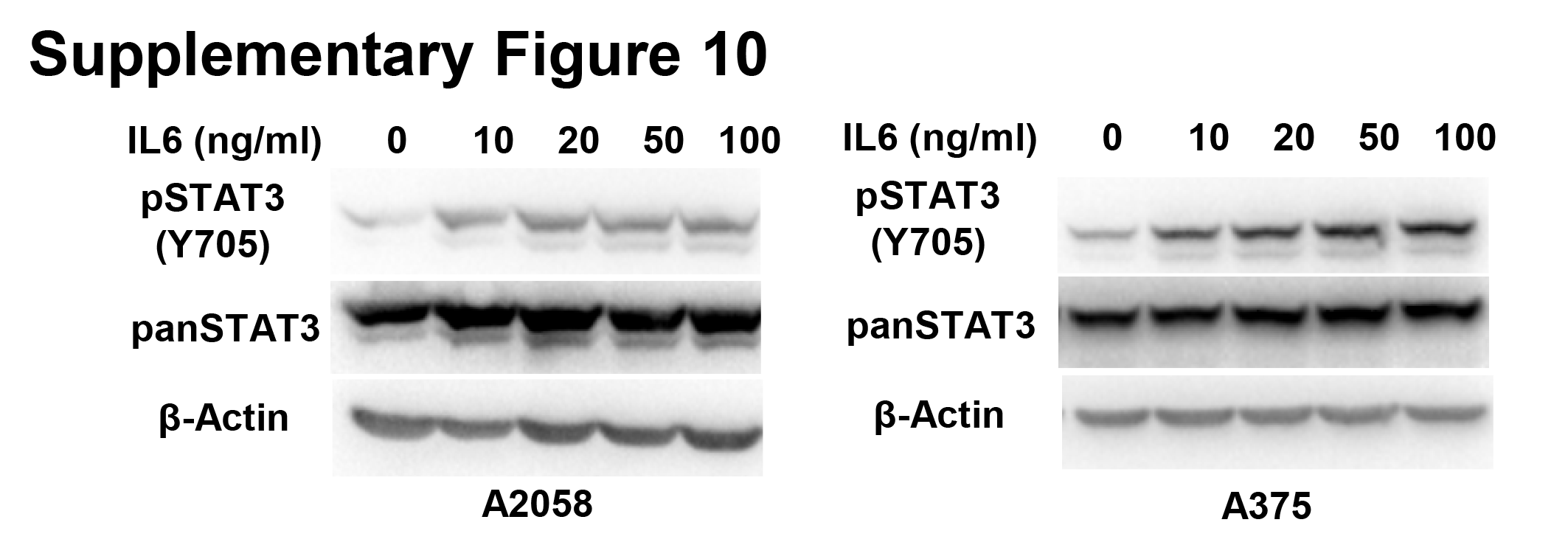

Supplement: Supplementary file 19 [file Image_10.tif]
